# De-implementing inappropriate benzodiazepine prescribing in primary care: an overview of systematic reviews informed by behavioral frameworks

**DOI:** 10.1186/s43058-026-00879-1

**Published:** 2026-02-21

**Authors:** Giuliano Duarte-Anselmi, Modesta Pousada Fernández, Beni Gómez-Zúñiga, Manuel Armayones Ruiz

**Affiliations:** 1 Facultad de Ciencias Médicas, Universidad de Santiago de Chile, Av. Libertador Bernardo O’Higgins 3363, Estación Central, Santiago, Chile; 2 Psychology and Educational Sciences Studies, eHealth Center, Universitat Oberta de Catalunya, Barcelona, Spain; 3 eHealth Center, Universitat Oberta de Catalunya (UOC), Barcelona, Spain

**Keywords:** Deprescribing, Low-value care, Benzodiazepines, Implementation, Primary care, Behavioral frameworks

## Abstract

**Background:**

The long-term use of benzodiazepines (BZDs) poses significant health risks, including cognitive impairment, falls, and dependency, despite guidelines recommending against prolonged use. Effective deprescribing interventions are essential, but evidence on optimal strategies in primary healthcare remains fragmented.

**Objective:**

This overview synthesizes evidence from systematic reviews of interventions aimed at reducing inappropriate BZD prescribing in primary care. It identifies barriers and facilitators, assesses the use of behavioral theories, evaluates intervention effectiveness, and appraises methodological quality.

**Methods:**

A comprehensive literature search was conducted across four electronic databases (MEDLINE via PubMed, Cochrane Database of Systematic Reviews, Epistemonikos, and PsycINFO) up to September 15, 2024. Systematic reviews evaluating deprescription strategies targeting healthcare professionals in primary care settings were included. Two independent reviewers screened studies and extracted data on intervention characteristics, behavior change techniques, and outcomes. The AMSTAR-2 tool was used to assess methodological quality.

**Results:**

From 2,577 records identified, 14 systematic reviews met inclusion criteria, comprising 279 primary studies with minimal overlap (Corrected Covered Area = 1.24%). The majority of interventions were randomized controlled trials from high-income countries, with only one review including a lower-middle-income country. Common deprescribing strategies were gradual dose reduction (71%), patient education (50%), cognitive-behavioral therapy (43%), and pharmacist-led interventions (36%). Only one review explicitly used a behavioral theory. Key barriers included patient dependency, fear of withdrawal, provider resistance, insufficient training, low self-efficacy, and limited healthcare resources. Facilitators were structured education, shared decision-making, pharmacist involvement, and goal-setting. Multifaceted interventions that integrated behavioral components, especially those involving pharmacists and structured patient education, consistently demonstrated greater and more sustained effectiveness.

**Conclusions:**

This overview demonstrates that multifaceted interventions—particularly those integrating patient education, audit-and-feedback, and pharmacist involvement—are the most effective for reducing inappropriate benzodiazepine use in primary care. Evidence also suggests that even brief, theory-informed interventions can achieve moderate effectiveness. Despite this progress, most studies lack explicit theoretical frameworks, underscoring the need for theory-driven approaches to enhance intervention design, implementation, and sustainability. Future research should focus on patient-centered strategies, long-term adherence, and broader representation from diverse socioeconomic settings. Registered in PROSPERO (CRD42024548653).

**Supplementary Information:**

The online version contains supplementary material available at 10.1186/s43058-026-00879-1.

Contributions to the literature

- This is the first overview of systematic reviews to examine behavioral and implementation strategies targeting the de-implementation of inappropriate benzodiazepine prescribing in primary care.
- It assesses the extent to which behavioral frameworks such as COM-B, the Theoretical Domains Framework (TDF), and behavior change techniques (BCTs) are explicitly used in deprescribing interventions.
- The review reveals key gaps in theory-informed intervention design and evaluation.
- It provides practical guidance for integrating behavioral science more effectively into implementation strategies to reduce low-value care.

## Introduction

Benzodiazepines (BZDs) remain among the most frequently prescribed psychotropic medications worldwide and represent a persistent public health concern. Although effective for short-term management of anxiety and insomnia, prolonged use is associated with dependence, cognitive decline, falls, and increased healthcare utilisation, especially among older adults [1, 2]. Major international guidelines recommend limiting their use to four weeks or less, yet chronic prescribing remains common across healthcare systems [3, 4].

Recent global data further highlight the scale of the problem. A cross-national analysis across 67 countries, showed that benzodiazepine and Z-drug consumption has remained consistently high over the past decade [5]. Use increases sharply with age: over 30% of community-dwelling older adults in several European nations are prescribed BZDs, with even higher rates in hospital and long-term care settings [6–8]. In the United States, benzodiazepine receptor agonists were the most frequently prescribed potentially inappropriate medications among older adults in 2020 [9]. These trends underscore the need for effective, scalable deprescribing strategies within primary care, where most BZD prescriptions originate and can be addressed.

Reducing inappropriate prescribing is a form of de-implementation—the systematic discontinuation of low-value or harmful healthcare practices [10–12]. Implementation science frameworks such as the COM-B model (Capability, Opportunity, Motivation–Behaviour), the Behaviour Change Wheel (BCW), and the Theoretical Domains Framework (TDF) offer structured approaches to understanding and modifying prescriber behaviour [13–16]. These models can guide the design of interventions that not only reduce unnecessary prescribing but also enhance sustainability through behavioural and contextual alignment. Such efforts are essential to translate evidence-based recommendations into routine practice, addressing behavioural and contextual barriers that sustain inappropriate prescribing [17, 18].

To promote consistency and evaluation, de-implementation strategies are often described using standardized taxonomies such as ERIC and the Behaviour Change Technique (BCT) taxonomy [19–21]. Recent evidence has highlighted that, although taxonomies such as the Expert Recommendations for Implementing Change (ERIC) and the Behaviour Change Technique (BCT) taxonomy have improved the classification and consistency of implementation strategies, important gaps remain in how these strategies are operationalized and reported in practice, particularly regarding clinician accountability and structured communication processes [21, 22].

Previous initiatives have demonstrated that interventions combining multiple components—such as patient education, audit-and-feedback, and pharmacist involvement—tend to achieve greater reductions in inappropriate medication use than single strategies [1, 2, 22]. Nevertheless, the evidence base is fragmented, and implementation outcomes such as feasibility, fidelity, and sustainability are seldom assessed.

In the present manuscript, we use the term ‘de-prescribing interventions’ to refer to structured actions designed to discontinue or reduce inappropriate benzodiazepine use; ‘de-prescribing strategies’ to denote broader programmatic or policy approaches that support deprescribing at the system or practice level; and ‘intervention strategies’ to encompass specific techniques within interventions, such as tapering schedules, patient education, or audit-and-feedback. Evidence also indicates that even brief, theory-informed interventions may achieve moderate effects in supporting deprescribing within primary care [23]. Prior systematic reviews on benzodiazepine deprescribing have reported substantial variability in methodological rigor and scope, including inconsistent outcome measures, limited assessment of implementation outcomes, and absence of theoretical grounding in intervention design [24–26].

This overview of systematic reviews aims to synthesize the available evidence on strategies to reduce inappropriate benzodiazepine prescribing in primary care. Specifically, it seeks to (1) assess the effectiveness of deprescribing interventions, (2) identify methodological gaps and inconsistencies across reviews, and (3) examine the use of behavioral frameworks to inform future de-implementation research and practice.

## Methods

### Study design

This study is an overview of systematic reviews and adheres to the Cochrane Handbook for Systematic Reviews of Interventions [27] and the PRIOR (Preferred Reporting Items for Overviews of Reviews) statement [28]. The PRIOR checklist is reported in Supplementary Material Appendix 1.

### Protocol and registration

The protocol for this overview was prospectively registered in PROSPERO (CRD42024548653) on May 19, 2024 [29] (Supplementary Material Appendix 2). In writing and reporting this overview, we followed the Cochrane Handbook for Systematic Reviews of Interventions and the Preferred Reporting Items for Systematic Review and Meta-Analysis (PRISMA) guidelines [30]. The complete protocol can be downloaded from this link Open Science Framework (OSF).

### Eligibility criteria

The eligibility criteria were reported in detail in our protocol [29]. The inclusion criteria for this overview were based on the PICOS (population, intervention, comparison, outcome, study type) framework (Table 1):

**Table 1:** Eligibility criteria for elements of a comprehensive search strategy

Element	Inclusion and exclusion criteria
Population	**Included:** Studies that evaluated the reduction of BZD prescriptions in adults over eighteen years old, specifically those prescribed by physicians in primary healthcare settings, were included **Excluded:** Studies conducted in healthcare settings other than primary care, such as tertiary care, were excluded
Intervention	**Included:** Studies that evaluated the reduction of BZD prescribing in primary healthcare were included. Strategies aimed at behavior change to discontinue or reduce BZD prescribing by physicians and members of the primary healthcare team were considered **Excluded:** Studies that evaluated other healthcare settings, such as hospitalized patients (tertiary level of care), were excluded
Comparator	As this was an overview of systematic reviews, a comparator or control group was not required for inclusion in this study
Outcome	**Included:** The outcomes were not used as an inclusion criterion during the selection process. Any article that met all other inclusion criteria except for the outcome criterion was preliminarily included and evaluated in full text **Excluded:** Studies that did not include a primary or secondary outcome were excluded
Study design	**Included:** All systematic reviews (SRs)* that evaluated strategies to reduce BZD prescribing were included. Reviews with a broader scope than this summary were also considered, provided they presented separate data for the group of interest. The included reviews explicitly reported: (i) strategies or interventions to reduce BZD prescriptions; (ii) targeted physicians or teams of primary healthcare professionals; and (iii) focused on evaluating the suspension or reduction of BZD prescriptions as the main behavioral objective. Systematic reviews were considered as any secondary research that included only primary studies, followed an explicit methodology, and included a search performed in at least two databases. Both systematic reviews of randomized controlled trials and non-randomized studies were included **Excluded:** Studies that were not systematic reviews (e.g., primary studies, commentary articles, and conference proceedings) were excluded

^*^All systematic reviews (SRs) were included, operationally defined as any secondary research that included only primary clinical studies, with an explicit search strategy in at least two databases [27, 31]

### Information sources

This overview employed a comprehensive search strategy across MEDLINE (via PubMed), the Cochrane Database of Systematic Reviews (CDSR), Epistemonikos, and PsycINFO. To ensure completeness, reference lists of included reviews were screened, and supplementary searches were conducted, including manual reference checks, citation searching (*n* = 274), and additional records from websites (*n* = 6) and organizations (*n* = 9). All sources were critically evaluated, with search details documented for transparency, and search strategies are available in Supplementary Material Appendix 3.

### Search strategy

The electronic search strategy (Supplementary Material Appendix 4–5) was conducted from database inception to September 15, 2024, with no restrictions. Additional searches included manual bibliography screening and outreach to authors and experts to identify further eligible studies. Eligibility criteria are detailed in Table 1.

### Study selection process

After deduplication and pilot-testing, two independent reviewers (BG, MP) screened all titles, abstracts, and full-text articles for eligibility using the Rayyan ™ software [32]. Discrepancies were resolved through discussion, and a third reviewer (GD) was consulted when necessary. The list of studies excluded after full-text review, along with reasons for exclusion, is detailed in Supplementary Material Appendix 6.

### Data collection process

A data extraction tool was developed in Microsoft Excel based on Cochrane recommendations [33, 34]. One author (GD) initially extracted data from a systematic review, followed by independent extractions by two authors (BG, MP) from three additional reviews. Study selection and data extraction were independently conducted by BG and MP, with GD serving as a third reviewer to resolve discrepancies and ensure consistency. All authors (BG, MP, MA, GD) reviewed and refined the extracted data to ensure completeness and clarity (Supplementary Material Appendix 7). GD, who has advanced expertise in behavioral science and evidence synthesis, supervised the data extraction and ensured methodological consistency across all stages. All discrepancies during data extraction were discussed and resolved by consensus among the review team.

### Data items

Data items included systematic review characteristics, PICOS criteria, and variables related to digital behavior change intervention (Table 2).

**Table 2:** Data items in this overview of systematic reviews

Data items: Bibliographic Information (Author and Year, year of publication, Title, Aim of the SR) **Study Characteristics** • Total Number of Studies Included in the Review • Number of Randomized Controlled Trials Included • Type of Studies (Only RCTs, only non-RCTs -including experimental non-randomized and observational studies-, both RCTs and non-RCTs) • Year of the Most Recent Study Included • Period or Specific Date Range of the Literature Search **Population Characteristics** • Relatives (Support) (Whether family involvement in deprescribing is mentioned) • Health Professionals Other than Primary Care Physicians (Pharmacists, nurses, specialists, etc.) • Age Groups (Young adults, adults, older adults) • Country of Origin of the Studies (Geographic locations of included studies) **Intervention Details** • Discontinue or Reduce Prescribing (Whether the intervention focused on complete cessation or reduction of benzodiazepine prescribing) • Theoretical Foundation of the Intervention (Explicit mention of theoretical frameworks) • Analyzed Barriers to De-adoption (Challenges related to deprescribing) • Analyzed Facilitators to De-adoption (Factors that help in successful deprescribing) • Intervention Strategy for De-implementation (Key methods used to reduce BZD prescribing) **Effectiveness and Adherence** • Intervention Effectiveness (Results related to the effectiveness of deprescribing strategies) • Adherence to the De-implementation Strategy (Extent to which deprescribing recommendations were followed) **Quality Assessment and Risk of Bias** • Risk of Bias Tool Used (Cochrane, CONSORT, COREQ, etc.) • What is the Risk? (Summary of the risk of bias assessment) • Meta-analysis Conducted? (Yes/No) • Certainty of Evidence (GRADE) (High, Moderate, Low, Very Low) **Outcomes Assessed** • Medication Outcomes (Impact on benzodiazepine prescribing rates) • Clinical Outcomes (Effects on patient symptoms such as anxiety, withdrawal effects, and sleep quality) • System Outcomes (Changes in prescribing trends in healthcare settings) • Implementation Outcomes (Feasibility, adherence, patient and provider acceptability) • Unexpected Outcomes (Unintended positive or negative consequences) **Limitations and Gaps** • Limitations Reported by the Authors (Small sample sizes, methodological issues, language bias, etc.)	

### Quality appraisal of the included systematic reviews

Systematic reviews were assessed using AMSTAR-2 [35], evaluating aspects such as study selection, data extraction, risk of bias, and publication bias. Reviews were rated as high, moderate, low, or critically low confidence based on identified weaknesses. One author (GD) conducted the assessments, with consensus reached through discussion (Supplementary Material Appendix 8).

### Overlap in primary studies included in reviews

To ensure the accuracy of primary study outcome data and prevent double counting, we first evaluated whether the included systematic reviews shared overlapping primary studies. This assessment was conducted by creating a citation matrix and calculating the overall corrected covered area (CCA) using the GROOVE (Graphical Representation of Overlap for Overviews) tool [36]. The GROOVE tool provides a method for interpreting the extent of overlap, where a CCA of 0% to 5% indicates a slight overlap, 6% to 10% a moderate overlap, 11% to 15% a high overlap, and greater than 15% a very high overlap in primary studies cited in multiple systematic reviews.

### Data synthesis methods

The results of the included systematic reviews were synthesized narratively. A summary table was created to present key characteristics, intervention strategies, barriers, facilitators, and outcomes of each review (Table 4). The synthesis categorized interventions based on their approach, including gradual dose reduction, pharmacist-led interventions, behavioral interventions, and pharmacological support. Outcomes were analyzed across medication-related, clinical, economic, and implementation domains. Findings were mapped to identify common themes, highlight evidence gaps, and assess the use of behavior change frameworks. Detailed data and methodological specifics are available in (Supplementary Material Appendix 9).

### Deviations from the protocol

As a post-hoc addition to enrich our behavioral analysis, we conducted a two-stage inferential mapping process using the Theoretical Domains Framework (TDF) by Cane et al. [16] and the Behavior Change Technique Taxonomy version 1 (BCTTv1) [19, 20], consistent with methodological procedures previously applied in recent overviews of behavior change interventions [49]. First, we assessed the presence of TDF domains by reviewing the descriptions of intervention strategies and contextual factors in each systematic review. While only one review (Lynch et al., 2020) explicitly applied the TDF, additional domains were inferred based on narrative descriptions. Second, we extracted or inferred behavior change techniques (BCTs) by identifying references—explicit or implicit—to the 93 techniques in the BCTTv1 [19]. When descriptions were too general to map specific BCTs, we recorded behavioral strategies and TDF domains separately in Table 5 and Appendix 10, but excluded them from Table 6. Both mappings were conducted independently by two reviewers trained in behavioral science, with discrepancies resolved through consensus. This enriched behavioral coding enabled a more structured understanding of the mechanisms of action underpinning BZD deprescribing interventions. Results of this mapping are summarized in Tables 5, and 6, Supplementary Material Appendix 10.

## Results

**Fig. 1:**
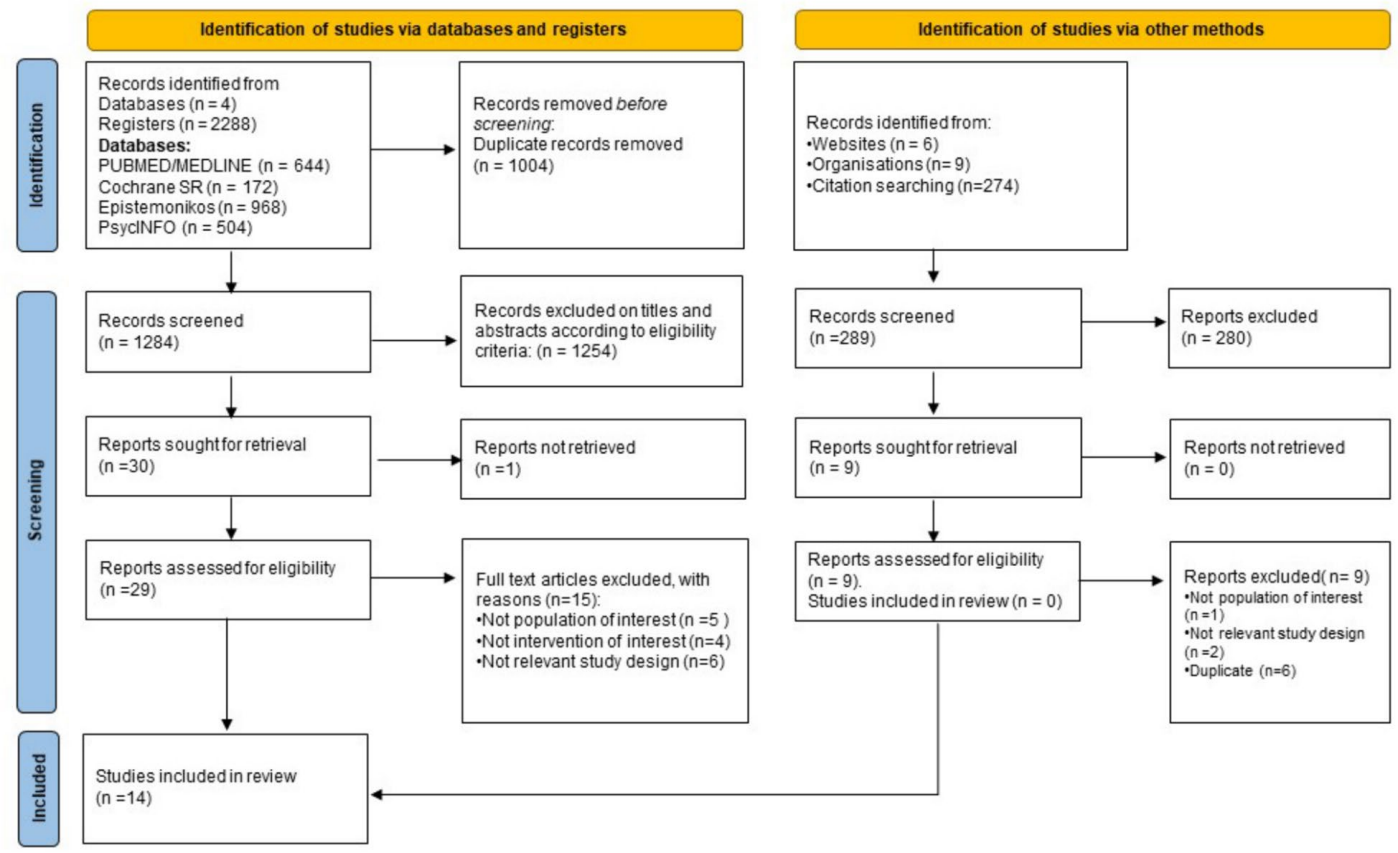
Flow diagram of study selection based on PRIOR (Preferred Reporting Items for Overviews of Reviews) guidelines

A total of 2,577 records were identified, including 2,288 from electronic databases and 289 from additional bibliographic sources. After removing duplicates and screening titles and abstracts, 29 full-text articles were assessed for eligibility. Of these, 15 were excluded based on predefined criteria, resulting in the inclusion of 14 systematic reviews in this overview [1, 2, 37–48] (Fig. 1). To enhance the comprehensiveness of the search, supplementary strategies were applied, such as citation tracking and targeted website searches (Supplementary Material Appendix 3).

### Characteristics of systematic reviews

The 14 systematic reviews included in this overview were published between 2014 and 2024 and collectively encompassed 352 primary studies. More than half (*n* = 8) were published within the past four years (2020–2024). The number of studies included per review varied substantially, from a minimum of 3 (Mugunthan et al., [42]) to a maximum of 113 (Brandt et al., [39]), the latter also incorporating clinical practice guidelines. Most reviews predominantly included randomized controlled trials (RCTs), either exclusively—as in Baandrup et al., 2018 (*n* = 35) [38] and Parr et al., [45] (*n* = 32) – or in combination with non-randomized designs, including observational and qualitative studies (Supplementary Material Appendix 9).

According to the World Bank classifications [50], all reviews primarily focused on high-income countries (*n* = 14), with only one review [37] reporting a study conducted in a lower-middle-income country (India). No reviews identified studies from upper-middle-income or South American countries. Detailed characteristics of the individual systematic reviews are presented in Supplementary Material Appendix 9. The synthesis of study characteristics in all 14 systematic reviews is shown in Table 3.

**Table 3:** Main characteristics of Systematic Reviews (SRs)

Characteristics of systematic reviews (*n* = 14**)		Results
**Systematic review design**	Number of studies included	352*
	Range of years of the study included	(2009–2023)
	Place (country -geographic location)	High-income countries (*n* = 14); Lower-middle-income countries (*n* = 1-India); Upper-middle-income countries (*n* = 0)
	Only RCT	7 (50%)
	Only non-RCT (includes non-randomized experimental and observational studies)	5 (36%)
	Mixed methods	1 (7%)
	Qualitative	1 (7%)
**Population type**	Older adults (≥ 60 years)	10 (71%)
	Adults aged ≥ 18 (General adult population)	4 (29%)
	Healthcare professionals other than general practitioners	7 (50%)
	Family or caregiver support	1 (7%)
**Intervention strategies**	Gradual dose reduction (GDR)	10 (71%)
	Educational or informational interventions (letters, brochures)	7 (50%)
	Cognitive Behavioral Therapy (CBT)	6 (43%)
	Pharmacological supportive	5 (36%)
	Brief interventions	3 (21%)
	Pharmacist-led interventions	5 (36%)
	GDR combined with CBT	5 (36%)
**Type of outcomes**	Medication outcomes	13 (93%)
	Clinical outcomes	6 (43%)
	Economic outcomes	1 (7%)
	Implementation outcomes	1 (7%)
**Other descriptions**	Barriers and facilitators were analyzed	2 (14%)
	Interventions based on behavioral theories	1 (7%)
**Meta-analysis and** **certainty of evidence**	Meta-analysis reported	7 (50%)
	Certainty of Evidence (GRADE) reported	3 (21%)

^*^Total number of primary studies included in the systematic reviews of this overview

^**^Total number of systematic reviews included in this overview

### Characteristics and behavioral components of the interventions

Interventions analyzed within the reviews varied considerably in their behavioral components, target populations, and complexity (Table 4). The most common strategies were gradual dose reduction (GDR, 71%), patient education through informational materials (50%), cognitive-behavioral therapy (CBT, 43%), pharmacist-led interventions (36%), and brief interventions such as motivational letters (21%) (Table 5). Strategies combining GDR with CBT or educational interventions were frequently recommended or implemented.

**Table 4:**
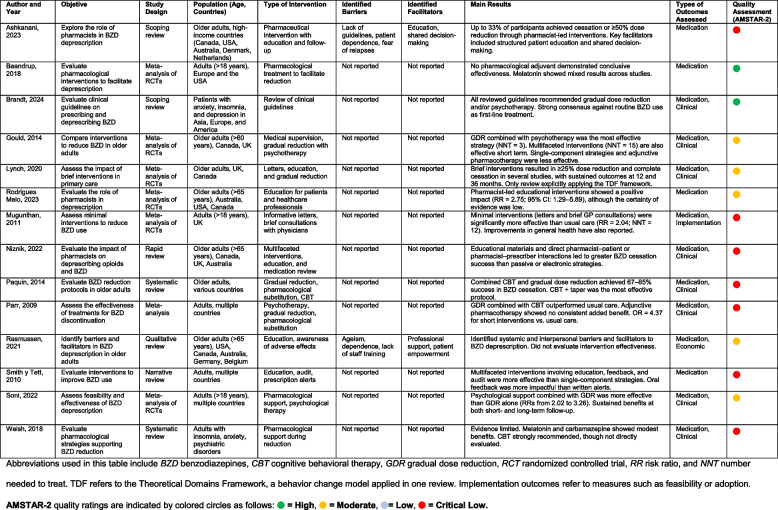
Summary of systematic reviews on benzodiazepine deprescribing: study characteristics, interventions, barriers, facilitators, and outcomes [2, 37–48]

**Table 5:** Behavior change strategies and theoretical domains framework (tdf) domains identified in systematic reviews on benzodiazepine deprescribing

Authors	Intervention Strategy											Theoretical Domains Framework (TDF)	BCT codification level
	**Motivational conversation**	**Education**	**Counseling**	**Shared decision making**	**Cognitive-behavioral therapy**	**Psycotherapy**	**Encouragement**	**Support and follow-up**	**Self-help information**	**Behavioral interventions**	**Providing with advise**		
Ashkanani 2023 [37]	**x**	**x**	**x**	**x**	**x**							Knowledge; Beliefs about consequences; Environmental context and resources; Social influences; Behavioral regulation	Explicit/inferable
Brandt 2024 [39]				**x**	**x**							Knowledge; Beliefs about consequences; Environmental context and resources	Limited – general strategies only
Gould 2014 [2]		**x**				**x**						Knowledge; Skills; Beliefs about capabilities; Social influences; Behavioral regulation	Limited – general strategies only
Lynch 2020 (*) [40]		**x**					**x**	**x**	**x**			Knowledge; Emotion; Beliefs about consequences; Beliefs about capabilities; Reinforcement; Environmental context and resources; Social influences; Memory, attention and decision processes; Behavioral regulation	Explicit/inferable
Melo et al. 2023 [41]		**x**										Knowledge; Environmental context and resources; Social influences	Limited – general strategies only
Mugunthan 2011 [42]									**x**			Knowledge; Memory, attention and decision processes	Explicit/inferable
Niznik 2022 [43]		**x**								**x**		Knowledge; Social/professional role and identity; Social influences; Environmental context and resources	Explicit/inferable
Paquin 2014 [44]				**x**	**x**			**x**				Knowledge; Beliefs about consequences; Goals; Behavioral regulation	Explicit/inferable
Parr 2009 [45]										**x**	**x**	Knowledge; Beliefs about consequences; Social influences	Explicit/inferable
Smith and Tett 2010 [47]		**x**										Knowledge; Social influences; Environmental context and resources; Behavioral regulation	Explicit/inferable
Soni et al. [1]		**x**			**x**					**x**		Knowledge; Beliefs about consequences; Skills; Behavioral regulation	Explicit/inferable
Baandrup 2018 [38]												Not applicable	Not applicable
Rasmussen 2021 [46]												Not applicable	Not applicable
Welsh 2018 [48]						**x**						Knowledge; Beliefs about consequences	Explicit/inferable

This table summarizes the presence of behaviorally oriented strategies across the 14 systematic reviews included in this overview. An "x" indicates that the strategy was either implemented, tested, or explicitly reported as a core component of the interventions evaluated. Strategies include motivational and cognitive approaches (e.g., counseling, CBT), educational tools, provider-patient communication processes (e.g., shared decision-making), and structured follow-up. Reviews that focused on contextual factors (e.g., Rasmussen et al., 2021), pharmacological support (e.g., Baandrup et al., 2018), or adjunctive therapies (e.g., Welsh, 2018) are included for completeness, although they did not assess behavioral interventions directly. The last column indicates the theoretical domains from the Theoretical Domains Framework (TDF) identified in each review. Domains were inferred by two independent reviewers based on intervention descriptions, except for Lynch et al. (2020), which explicitly reported the TDF in its analysis (*). The final column indicates whether BCTs could be reliably identified. Some reviews included general behavioral strategies (e.g., education or shared decision-making), but lacked sufficient detail for BCT coding and were thus excluded from Table 6.

Behavioral elements such as motivational conversations, counseling, shared decision-making, encouragement, structured follow-up, and self-help information varied significantly across reviews. Interventions often engaged pharmacists or primary care physicians, while the involvement of other healthcare providers (e.g., psychologists, social workers) was rarely mentioned (Table 5). Two reviews, Baandrup et al. [38] and Rasmussen et al. [46], did not detail specific behavioral interventions. Baandrup exclusively assessed pharmacological treatments, whereas Rasmussen focused on qualitatively identifying barriers and facilitators without evaluating specific intervention effectiveness (Supplementary Material Appendix 9).

### Effectiveness of interventions

Overall, multifaceted and behaviorally informed interventions were consistently more effective than single-component approaches. Pharmacist-led interventions showed promising results, with significant reductions in benzodiazepine (BZD) use or dosage. For instance, Ashkanani et al. [37] reported that up to 33% of participants achieved cessation or a ≥ 50% dose reduction, and Rodrigues Melo et al. [41] observed a statistically significant improvement (RR = 2.75, 95% CI: 1.29–5.89), though evidence certainty was low.

Psychological and behavioral strategies, particularly CBT combined with GDR, consistently demonstrated high effectiveness. Gould et al. [2] indicated that supervised withdrawal with psychotherapy yielded an NNT of 3. Similarly, Soni et al. [1] reported superior effectiveness of GDR combined with psychological support compared to GDR alone (RRs ranging from 2.02 to 3.26), while Paquin et al. [44] highlighted success rates of 67–85% for CBT plus tapering protocols.

Brief interventions also showed effectiveness. Lynch et al. [40] and Mugunthan et al. [42] reported moderate effects, with Mugunthan et al. presenting a combined RR of 2.04 (95% CI: 1.5–2.8, NNT = 12). These outcomes were sustained for up to 36 months in some cases. Conversely, pharmacological support alone, evaluated by Baandrup et al. [38] and Welsh [48], demonstrated limited or inconsistent evidence of effectiveness. Agents such as melatonin and carbamazepine showed modest benefits that were not consistently superior to placebo or GDR alone (Table 4).

### Behavioral foundations, barriers, and facilitators

Despite the behavioral orientation of most interventions, only Lynch et al. [40] explicitly utilized a behavioral theoretical framework—the Theoretical Domains Framework (TDF)—to inform their analysis. Other reviews, despite incorporating strategies like CBT, shared decision-making, and motivational approaches, did not explicitly detail theoretical underpinnings (Table 5).

Barriers and facilitators were systematically evaluated in two reviews [37, 46]. Common barriers included patient dependence, withdrawal anxiety, absence of clear deprescribing guidelines, limited clinician training, and inadequate resources. Facilitators identified were structured patient education, multidisciplinary collaboration, shared decision-making, active patient participation, and systematic provider support (Table 4).

Follow-up durations varied broadly, from as brief as eight days to as long as 36 months.

Most reviews (93%) reported medication-related outcomes, such as reductions in benzodiazepine prescriptions or dosage levels, while fewer assessed clinical outcomes (43%), including withdrawal symptoms, cognitive function, or sleep quality. However, only one review [42] evaluated implementation-specific outcomes such as feasibility, acceptability, or sustainability, highlighting a major evidence gap regarding how deprescribing strategies are adopted, delivered, and maintained in real-world primary care settings. Details are presented in Tables 3 and, Supplementary Appendix 9.

### Application of behavior change techniques in deprescribing interventions

Several reviews demonstrated that specific behavior change techniques (BCTs), either explicitly reported or inferred from intervention descriptions, contributed meaningfully to the effectiveness of deprescribing strategies (Table 6). Reviews with insufficient detail for individual BCT coding were still included in the broader mapping of behavioral strategies and TDF domains (Appendix 10 and Table 5). In Ashkanani et al. (2023) [37], pharmacist-led interventions incorporated structured education on the risks of prolonged benzodiazepine (BZD) use (BCT 5.1 – Information about health consequences), the setting of individualized tapering goals (BCT 1.1 – Goal setting (behavior)), and the use of pharmacists as credible sources of information (BCT 9.1 – Credible source). These elements were associated with significant clinical impact, including up to 33% of patients achieving complete cessation or at least a 50% dose reduction.

**Table 6:**
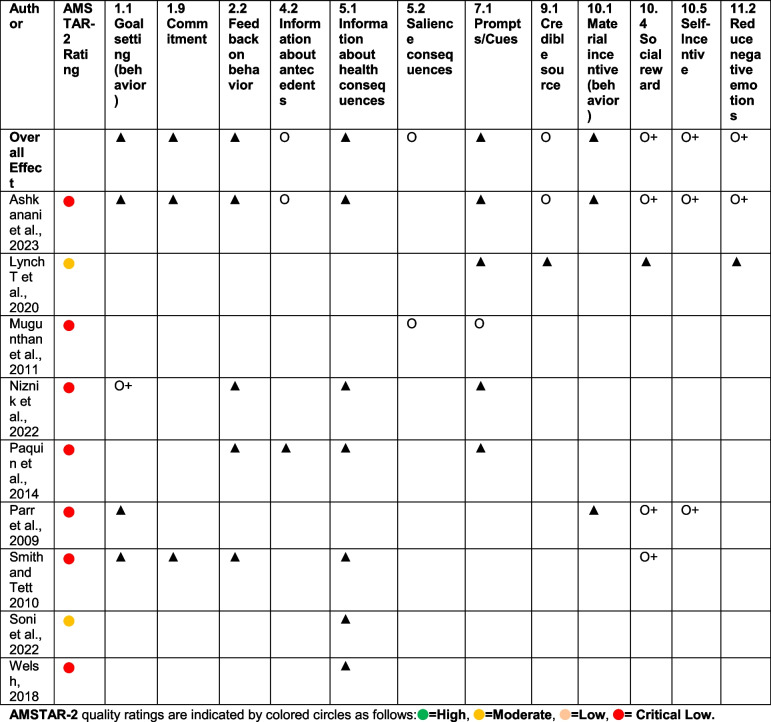
Summary of behavior change techniques in benzodiazepine deprescribing interventions [37, 40, 42–45, 47, 48]

Classification of effectiveness from current systematic reviews and meta-analyses: ▲ = Positive effect of BCT based on good evidence such as subgroup or regression analyses. O+ = Positive effect of BCT based on low evidence such as frequency of individual BCTs within effective interventions. O = Mixed evidence or no effect. O− = Negative effect of BCT based on low level of evidence such as frequency of individual BCTs within effective interventions.▼ = Negative effect of BCT based on good evidence such as subgroup or regression analyses. The following reviews were excluded from detailed BCT classification in Table 6 due to insufficiently specific descriptions of techniques to map individual BCTs reliably. However, these reviews are included in Table 5 and Appendix 10 for broader behavioral strategy and TDF domain inference: Brandt et al. (2024), Gould et al. (2014), and Rodrigues Melo et al. (2023). Baandrup et al. (2018) and Rasmussen et al. (2021) did not evaluate behavioral interventions and were excluded from both TDF and BCT mapping.

Similarly, Lynch et al. [40], the only review explicitly applying the Theoretical Domains Framework (TDF), described brief interventions comprising motivational letters and structured follow-up. These were mapped to BCT 7.1 – Prompts/cues, BCT 10.4 – Social reward, and BCT 11.2 – Reduce negative emotions, reflecting an effort to address both psychological barriers and emotional determinants of BZD use. The intervention achieved sustained reductions in dosage and discontinuation rates at 12- and 36-month follow-ups.

In Parr et al. [45], financial and motivational incentives were used to reinforce deprescribing behavior, aligning with BCT 10.1 – Material incentive (behavior), BCT 10.4 – Social reward, and BCT 10.5 – Self-incentive. These approaches yielded significantly better outcomes than usual care in promoting discontinuation.

Paquin et al. [44] evaluated combined interventions using cognitive-behavioral therapy (CBT) and gradual dose reduction (GDR). These included BCT 1.1 – Goal setting (behavior), BCT 4.2 – Information about antecedents, and BCT 5.1 – Information about health consequences, with cessation success rates ranging from 67 to 85%.

### Quality appraisal of the included systematic reviews

**Fig. 2:**
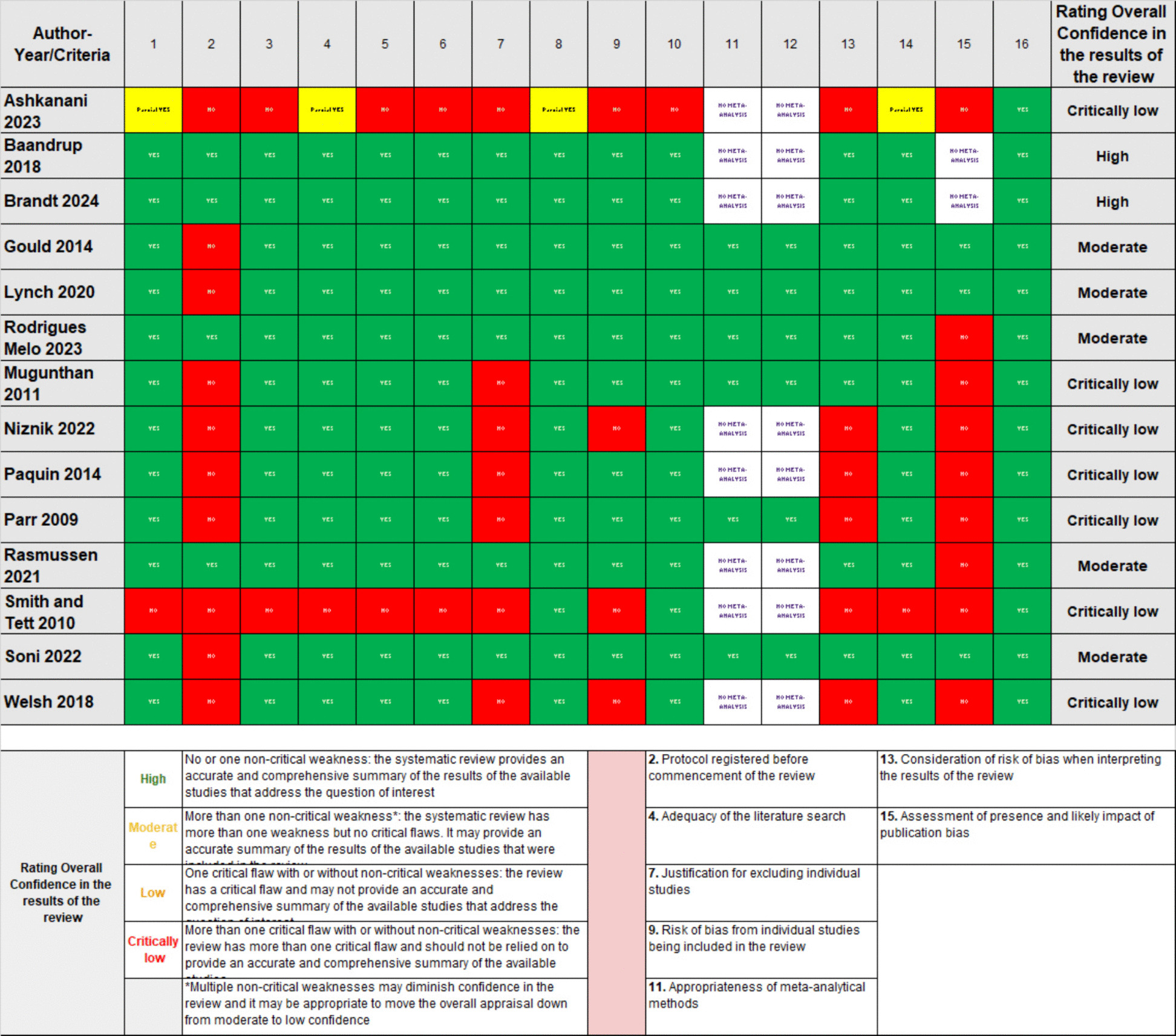
Critical appraisal outcomes based on AMSTAR 2 (A Measurement Tool to Assess Systematic Reviews)

Overall, confidence in the results of the systematic reviews was high in 14% (2/14) the reviews, moderate in 36% (5/14), low in 0% (0/14), and critically low in 50% (7/14). The most common methodological weaknesses included the omission of a registered review protocol (10/14, 71%), lack of a complete list of excluded studies with justification (7/14, 50%), absence of publication bias assessment (6/14, 43%), and lack of formal risk of bias assessment for included studies (4/14, 29%). (Fig. 2, Supplementary Material Appendix 11).

### Overlap in primary studies included in reviews

**Fig. 3:**
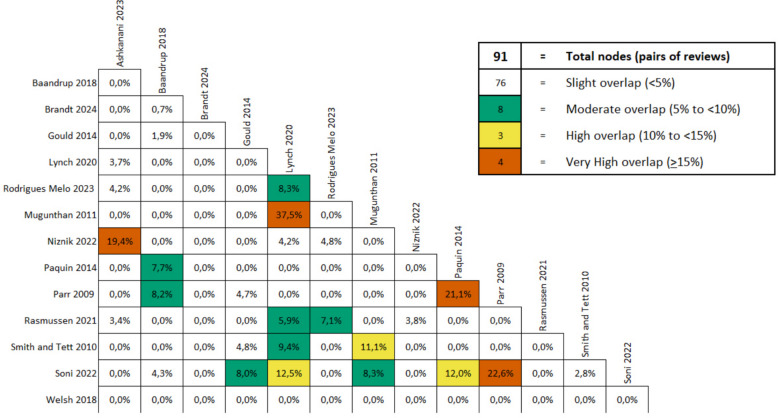
Overlap of primary studies among review pairs using the GROOVE (Graphical Representation of Overlap for Overviews) tool

The overlap assessment revealed that most primary studies were cited only once across the systematic reviews. Overall, there was a minimal overlap in the 279 primary studies included in the 14 systematic reviews, with a Corrected Covered Area (CCA) of 1.24%. Comparisons between pairs of systematic reviews indicated that 83.5% (76 out of 91) had a low overlap (< 5%), 8.8% (8 out of 91) had a moderate overlap (5% to < 10%), 3.2% (3 out of 91) had a high overlap (10% to < 15%), and 4.4% (4 out of 91) had a very high overlap (≥ 15%). Figure 3 and detailed in Supplementary Material Appendix 12 and 13.

## Discussion

This overview systematically synthesized the existing evidence from 14 systematic reviews evaluating strategies for benzodiazepine (BZD) deprescribing in primary care. The analysis identified a considerable diversity of interventions, varying widely in their complexity, duration, and behavioral components. Despite clear clinical guidelines advocating against prolonged BZD prescribing, translating these recommendations into practice remains challenging, influenced by multiple contextual and behavioral factors, including the limited integration of theory-informed approaches. These findings are consistent with evidence from deprescribing interventions targeting other medication classes, including opioids, proton pump inhibitors, and polypharmacy management, where multifaceted and behaviorally informed approaches have similarly demonstrated greater effectiveness and sustainability [24–26].

Multifaceted, behaviorally informed interventions—especially those involving structured education, pharmacist-led strategies, and psychological support—were consistently more effective than single-component or pharmacological approaches. These findings are in line with prior implementation research and underscore the importance of integrating behavioral strategies to address patient motivation, clinician engagement, and structured follow-up mechanisms. Interventions that applied cognitive-behavioral therapy (CBT), shared decision-making, or educational support showed improved outcomes, often sustained over time.

Pharmacist-led initiatives emerged as particularly effective, emphasizing the value of pharmacists as credible sources of information and facilitators of deprescribing. Interventions including BCTs such as goal-setting, prompts, and information about health consequences demonstrated greater impact compared to those that did not explicitly incorporate these components.

Despite the behavioral orientation of many interventions, only one review explicitly applied a behavioral framework (Lynch et al., [40]. While several reviews included components suggestive of behavior change strategies, many lacked the specificity required to code individual BCTs, highlighting the need for more detailed reporting in future studies.

The limited explicit use of behavioral frameworks does not necessarily indicate a lack of theoretical awareness among researchers. Many deprescribing interventions may have been implicitly informed by behavioral or psychological principles—such as patient motivation, clinician feedback, or structured reinforcement—without explicitly referencing a formal model. However, without clear theoretical articulation, it remains difficult to identify the mechanisms that drive successful change or to replicate interventions across diverse contexts. This limited use of explicit theory may also reflect practical barriers, including perceived complexity or limited familiarity with frameworks such as COM-B or TDF among researchers and practitioners. This observation is consistent with broader implementation research showing that the explicit use of theory to design or evaluate interventions remains uncommon, despite its recognized value in improving intervention reproducibility and understanding mechanisms of change [51, 52]. Future studies should not only adopt theory-informed designs but also report transparently how theoretical constructs are operationalized to guide intervention development, implementation, and evaluation.

### Strengths and limitations

A notable strength of this overview is its rigorous methodology, including comprehensive literature searches, systematic use of established frameworks (BCT taxonomy and TDF), transparent quality appraisal with the AMSTAR-2 tool, and an explicit assessment of study overlap among included systematic reviews. The overlap assessment revealed minimal redundancy among primary studies, with a Corrected Covered Area (CCA) of only 1.24%, indicating robustness in the diversity and comprehensiveness of the synthesized evidence. Comparisons between pairs of systematic reviews showed that 83.5% had low overlap (< 5%), further highlighting the methodological rigor and comprehensive nature of our findings.

However, approximately half of the included systematic reviews were rated as having critically low confidence according to AMSTAR-2 (Fig. 2). This was primarily due to methodological weaknesses such as missing registered protocols (71%), incomplete lists of excluded studies (50%), lack of publication bias assessment (43%), and absence of formal risk-of-bias evaluation (29%). These limitations constrain the overall certainty and generalizability of the synthesized evidence, reducing the strength of conclusions regarding intervention effectiveness. This finding also reflects broader methodological challenges in deprescribing research, where variability in review conduct and transparency hinders reproducibility and evidence translation.

Nevertheless, some limitations should be acknowledged. Variability in intervention descriptions occasionally necessitated inferential rather than explicit coding of behavioral techniques, potentially introducing subjectivity despite rigorous consensus-based methods. Furthermore, the predominance of studies from high-income countries significantly limits findings' applicability to more diverse, resource-limited settings.

### Implications for research and practice

Future research should focus on strengthening the theoretical and methodological foundations of deprescribing interventions by making theory use more explicit, transparent, and practically relevant. Rather than advocating for theory use per se, researchers should critically examine which frameworks (e.g., TDF, COM-B) add tangible value to intervention design and implementation, and how their constructs can be applied in feasible and context-sensitive ways [53]. Expanding research into low- and middle-income settings, and leveraging pharmacist-led and patient-centered strategies, represent promising avenues for achieving sustainable deprescribing outcomes. Finally, addressing barriers such as limited training, emotional challenges, and contextual constraints remains key to improving the real-world translation of evidence into practice [54].

## Conclusions

This overview underscores the efficacy of multifaceted, behaviorally informed interventions —particularly those combining patient education, audit-and-feedback, and pharmacist engagement— in reducing inappropriate benzodiazepine prescribing in primary care. In addition, evidence indicates that even brief, theory-informed interventions can achieve moderate deprescribing effects. Although explicit use of behavioral frameworks remains limited, integrating such approaches offers substantial potential to enhance intervention design, implementation, and sustainability. Addressing methodological limitations, such as study heterogeneity and limited representation from low-income settings, will be critical for strengthening global deprescribing practices. Policymakers and healthcare providers should leverage these behavioral insights to facilitate evidence-based deprescribing tailored to diverse patient and systemic needs.

## Supplementary Information

Additional file 1.

## Data Availability

All data generated or analyzed during this study are included in this published article.

## Abbreviations

AMSTAR-2: A Measurement Tool to Assess Systematic Reviews-2
BCT: Behavior Change Technique
BCW: Behaviour Change Wheel
BZDs: Benzodiazepines
CCA: Corrected Covered Area
CBT: Cognitive Behavioral Therapy
COM-B: Capability, Opportunity, Motivation–Behavior
ERIC: Expert Recommendations for Implementing Change
GDR: Gradual Dose Reduction
GRADE: Grading of Recommendations Assessment, Development and Evaluation
GROOVE: Graphical Representation of Overlap for Overviews
LVC: Low-value care
NNT: Number Needed to Treat
OR: Odds Ratio
PRIOR: Preferred Reporting Items for Overviews of Reviews
PRISMA: Preferred Reporting Items for Systematic Review and Meta-Analysis
RCT: Randomized Controlled Trial
RR: Risk Ratio
SR: Systematic Review
TDF: Theoretical Domains Framework
