# Supplementary material for: De-implementing inappropriate benzodiazepine prescribing in primary care: an overview of systematic reviews informed by behavioral frameworks
Source: Implement Sci Commun. 2026 Feb 21;7:58. doi: 10.1186/s43058-026-00879-1 (PMC13037225; doi:10.1186/s43058-026-00879-1)
Supplement: Supplementary file 1 — Additional file 1. [file 43058_2026_879_MOESM1_ESM.docx]

**De-implementing low-value practices in the prescription of benzodiazepines: an overview of systematic reviews–Multimedia Appendices**

Contents

[Appendix 1 – PRIOR Checklist 2](#_Toc192353149)

[Appendix 2. Registered in PROSPERO CRD42024548653 4](#_Toc192353150)

[Appendix 3 - Identification of studies via other methods (Grey Literature Sources) 10](#_Toc192353151)

[Websites 10](#_Toc192353152)

[Organizations 10](#_Toc192353153)

[Citation Searching 11](#_Toc192353154)

[Appendix 4 – Search strategy in line-by-line format 25](#_Toc192353155)

[Appendix 5 – Strategy in block format 26](#_Toc192353156)

[Appendix 6 – List of excluded studies 27](#_Toc192353157)

[Appendix 7– Data extraction form 29](#_Toc192353158)

[Appendix 8 – Risk of Bias Assessment (Critical Appraisal of Systematic Reviews) 31](#_Toc192353159)

[Appendix 9 – Characteristics of Systematic Reviews 35](#_Toc192353160)

[Appendix 14 – Participant Characteristics 39](#_Toc192353161)

[Appendix 14 a. Participant characteristics by age and target population 39](#_Toc192353162)

[Appendix 10 – Critical assessments based on AMSTAR 2 41](#_Toc192353163)

[Appendix 11 – Overlap in primary studies included in reviews 47](#_Toc192353164)

[Appendix 12. Matrix of evidence 48](#_Toc192353165)

Appendix 1 – PRIOR Checklist

**Reporting guideline for overviews of reviews of healthcare interventions: development of the PRIOR statement.**

| **Section**  Topic | **#** | **Item** | **Location reported on page #** |
| --- | --- | --- | --- |
| **TITLE** | | |  |
| Title | 1 | Identify the report as an overview of reviews. | 1 |
| **ABSTRACT** | | |  |
| Abstract | 2 | Provide a comprehensive and accurate summary of the purpose, methods, and results of the overview of reviews. | 2 |
| **INTRODUCTION** | | |  |
| Rationale | 3 | Describe the rationale for conducting the overview of reviews in the context of existing knowledge. | 2-3 |
| Objectives | 4 | Provide an explicit statement of the objective(s) or question(s) addressed by the overview of reviews. | 3 |
| **METHODS** | | |  |
| Eligibility criteria | 5a | Specify the inclusion and exclusion criteria for the overview of reviews. If supplemental primary studies were included, this should be stated, with a rationale. | 4, Table 1 |
|  | 5b | Specify the definition of ‘systematic review’ as used in the inclusion criteria for the overview of reviews. | 5 (note) |
| Information sources | 6 | Specify all databases, registers, websites, organizations, reference lists, and other sources searched or consulted to identify systematic reviews and supplemental primary studies (if included).  Specify the date when each source was last searched or consulted. | 5-6, Appendix 3,4 y 5 |
| Search strategy | 7 | Present the full search strategies for all databases, registers and websites, such that they could be reproduced. Describe any search filters and limits applied. | 5-6, Appendix 4-5 |
| Selection process | 8a | Describe the methods used to decide whether a systematic review or supplemental primary study (if included) met the inclusion criteria of the overview of reviews. | 6 |
|  | 8b | Describe how overlap in the populations, interventions, comparators, and/or outcomes of systematic reviews was identified and managed during study selection. | 8 |
| Data collection process | 9a | Describe the methods used to collect data from reports. | 6, Appendix 8 |
|  | 9b | If applicable, describe the methods used to identify and manage primary study overlap at the level  of the comparison and outcome during data collection. For each outcome, specify the method used to illustrate and/or quantify the degree of primary study overlap across systematic reviews. | 6-7, 15, Figure 2 and Appendix 16 |
|  | 9c | If applicable, specify the methods used to manage discrepant data across systematic reviews during data collection. | 6, Appendix 9 |
| Data items | 10 | List and define all variables and outcomes for which data were sought. Describe any assumptions made and/or measures taken to identify and clarify missing or unclear information. | 6-7, Textbox1 Appendix 8-9 |
| Risk of bias assessment | 11a | Describe the methods used to *assess* risk of bias or methodological quality of the included systematic reviews. | 8, Appendix 10 |
|  | 11b | Describe the methods used to *collect* data on (from the systematic reviews) and/or *assess* the risk of bias of the primary studies included in the systematic reviews. Provide a justification for instances where flawed, incomplete, or missing assessments are identified but not re-assessed. | 7 |
|  | 11c | Describe the methods used to *assess* the risk of bias of supplemental primary studies (if included). | NA |
| Synthesis methods | 12a | Describe the methods used to summarize or synthesize results and provide a rationale for the choice(s). | 8 |
|  | 12b | Describe any methods used to explore possible causes of heterogeneity among results. | 8 |
|  | 12c | Describe any sensitivity analyses conducted to assess the robustness of the synthesized results. | 8 |
| Reporting bias assessment | 13 | Describe the methods used to *collect* data on (from the systematic reviews) and/or *assess* the risk of bias due to missing results in a summary or synthesis (arising from reporting biases at the levels of the systematic reviews, primary studies, and supplemental primary studies, if included). | 14, Table 5, Appendix 10 |
| Certainty assessment | 14 | Describe the methods used to *collect* data on (from the systematic reviews) and/or *assess* certainty (or confidence) in the body of evidence for an outcome. | NA |
| **RESULTS** | | |  |
| Systematic review and supplemental primary study selection | 15a | Describe the results of the search and selection process, including the number of records screened, assessed for eligibility, and included in the overview of reviews, ideally with a flow diagram. | 9-20, Figure 1, |
|  | 15b | Provide a list of studies that might appear to meet the inclusion criteria, but were excluded, with the main reason for exclusion. | Appendix 7 |

From: (Gates M, Gates A, Pieper D, et al. Reporting guideline for overviews of reviews of healthcare interventions: development of the PRIOR statement. BMJ 2022;378:e070849. doi:10.1136/bmj-2022-070849.)

# Appendix 2. Registered in PROSPERO CRD42024548653


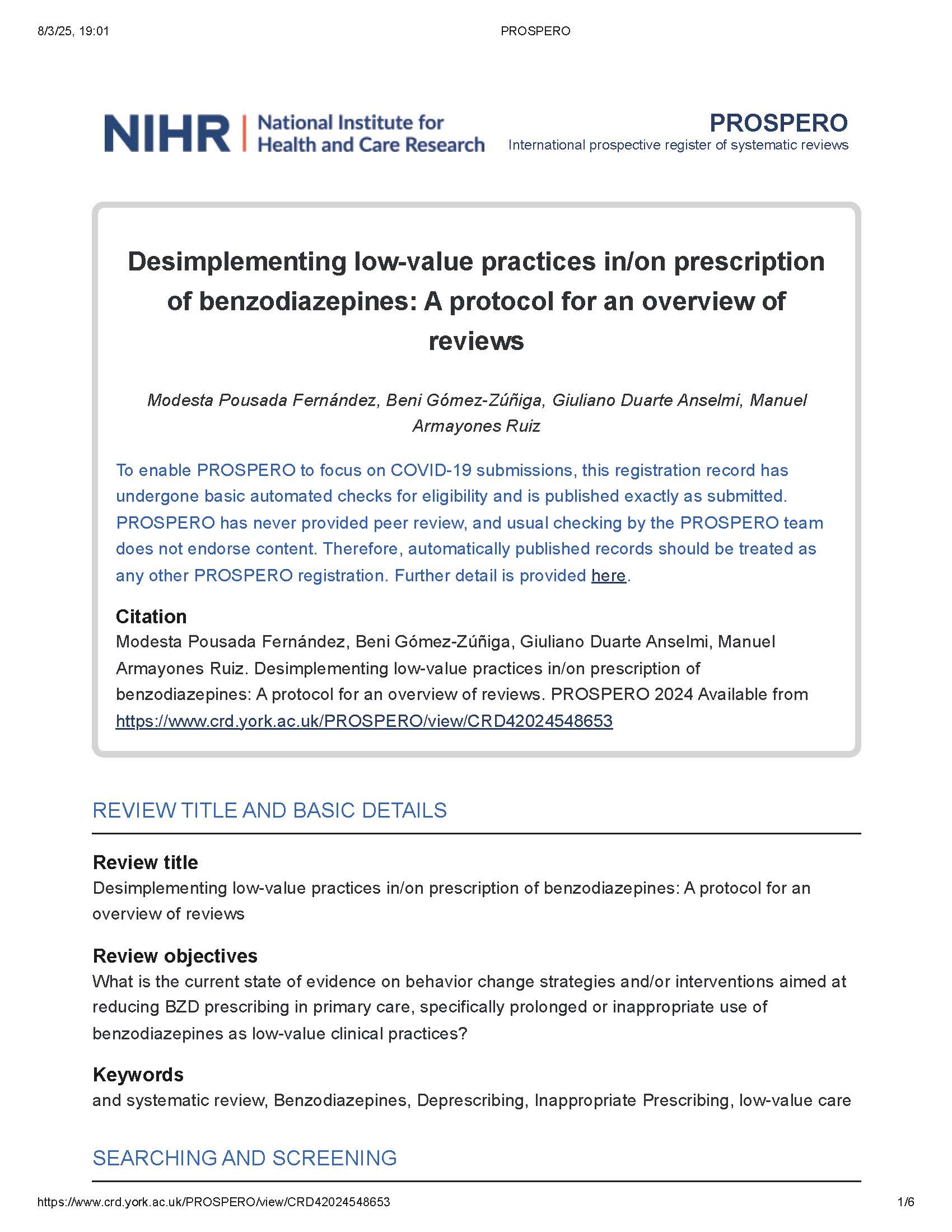


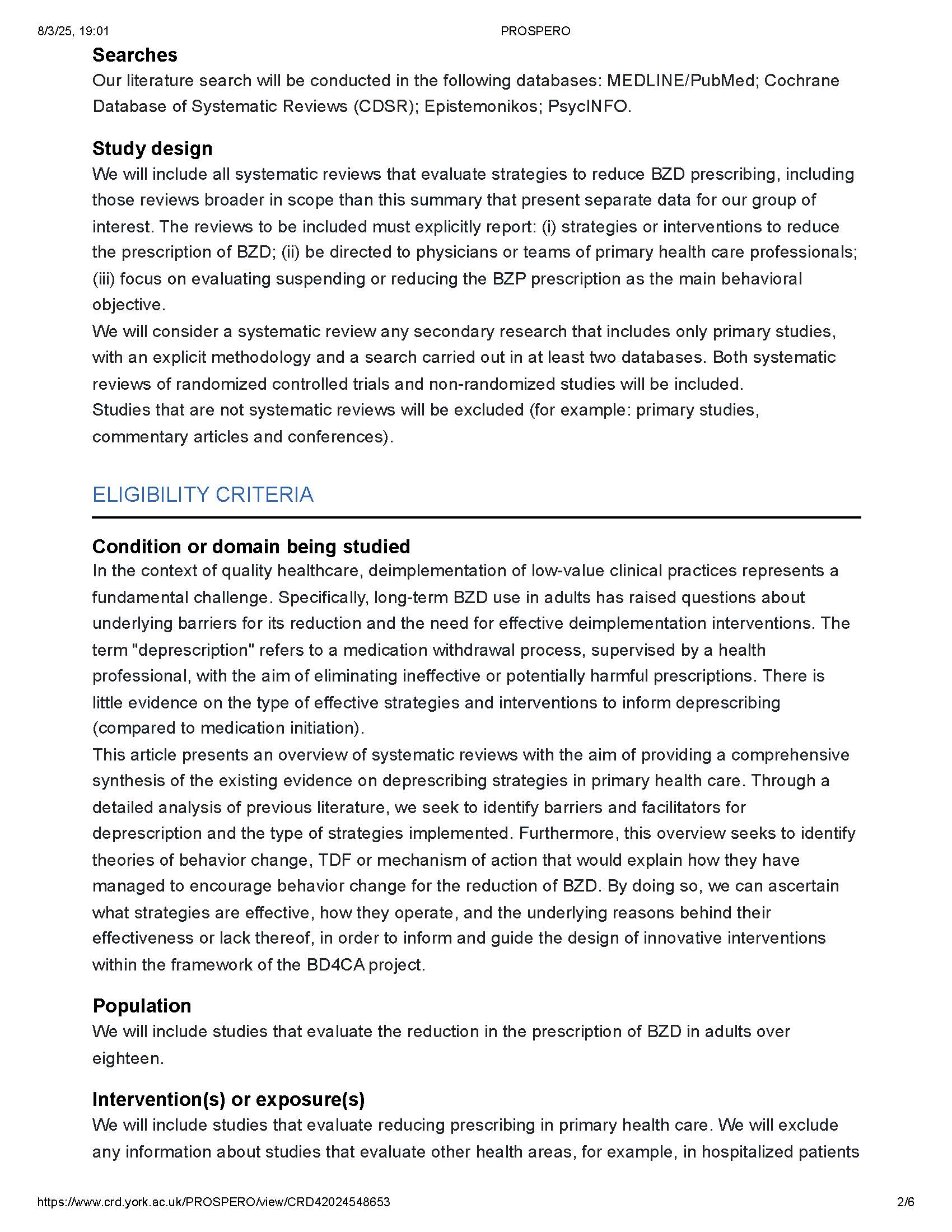

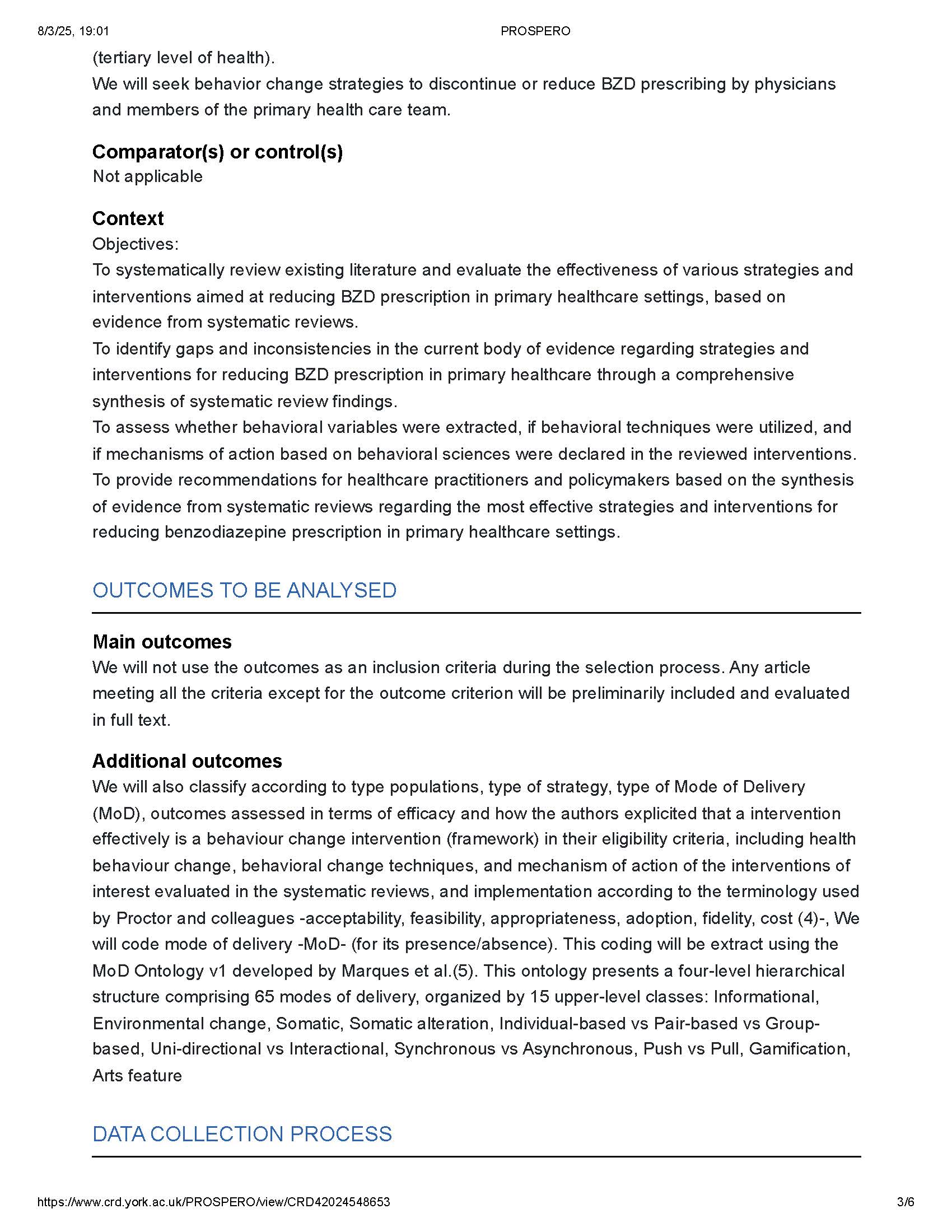

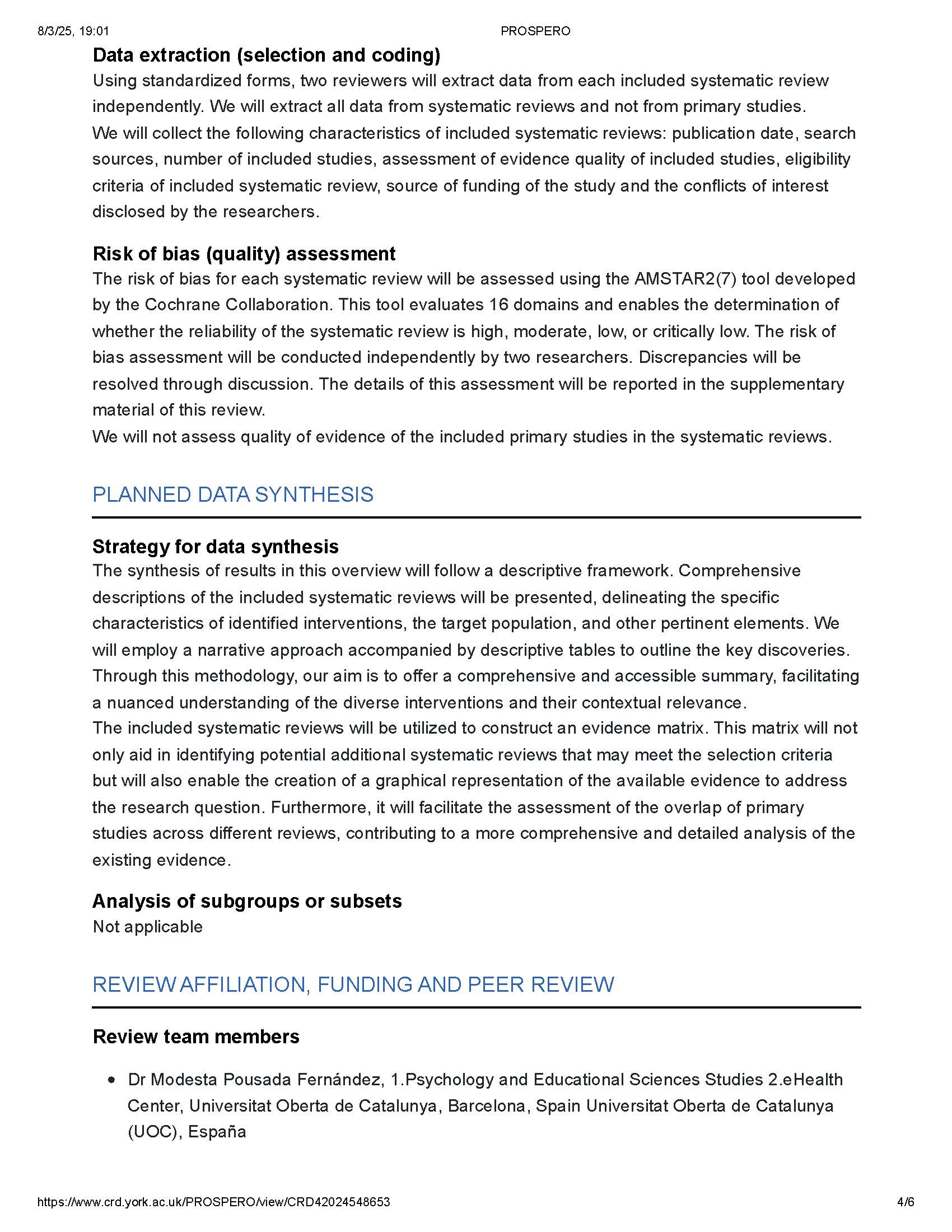

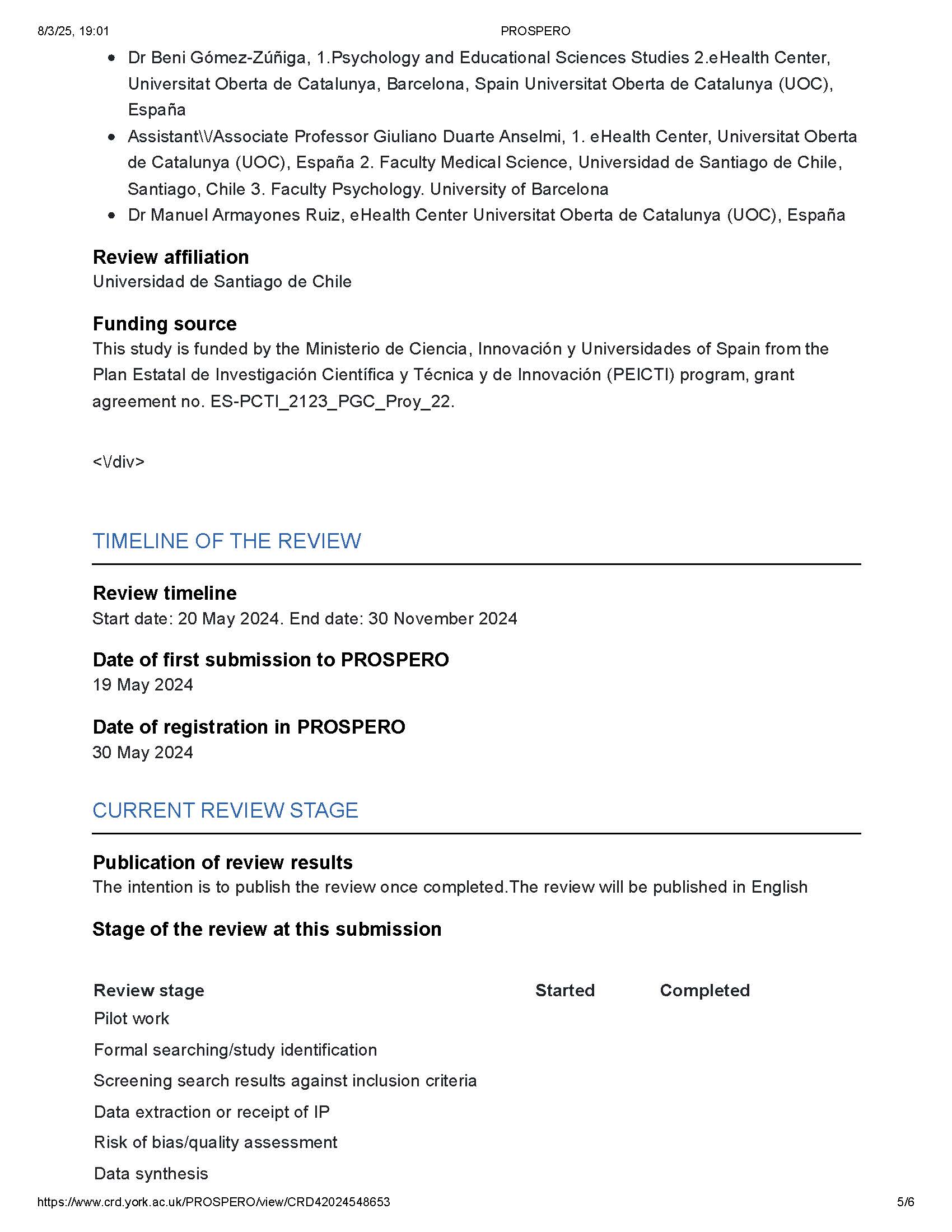

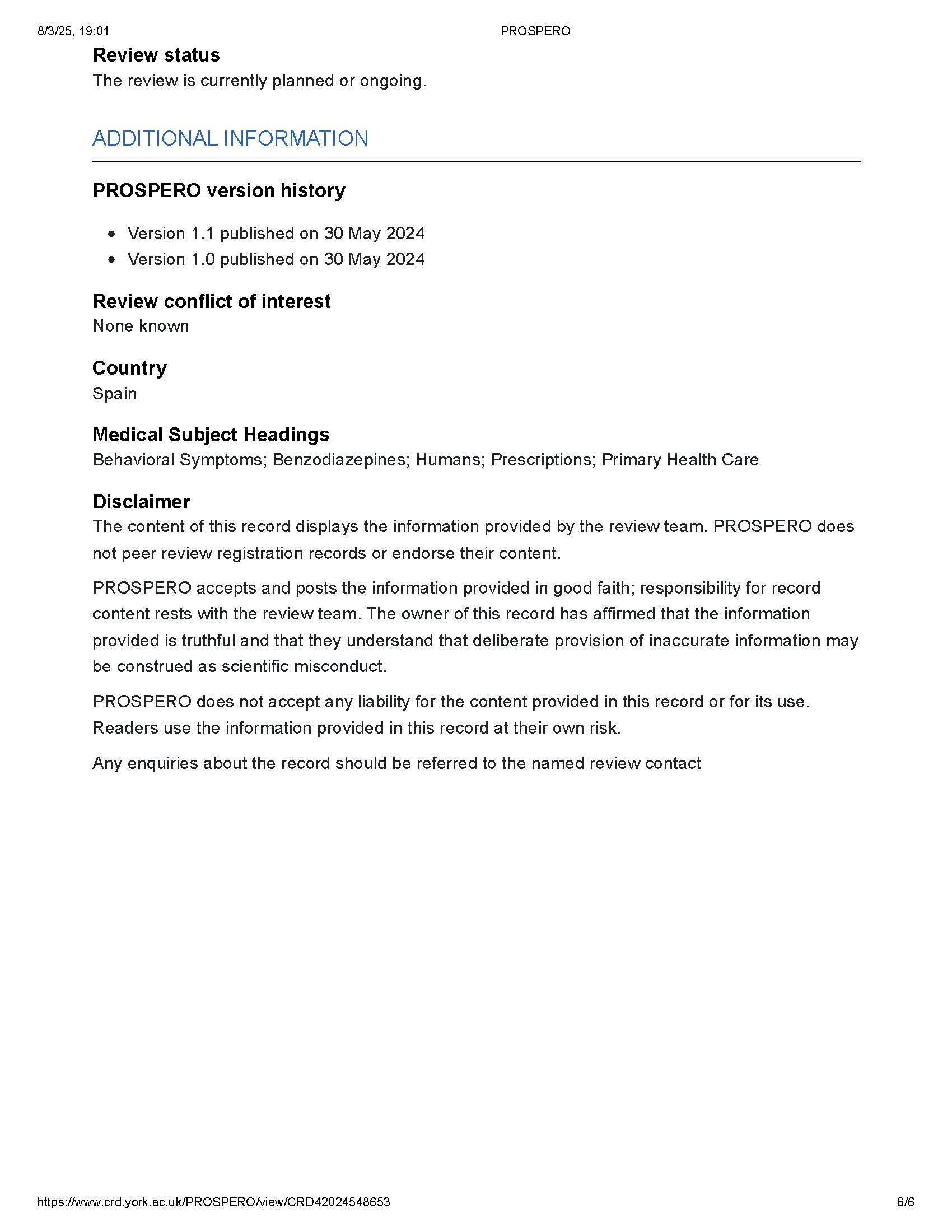


xx

# Appendix 3 - Identification of studies via other methods (Grey Literature Sources)

In addition to the primary search strategy, supplementary efforts were undertaken to ensure comprehensive identification of relevant studies. These efforts are detailed below:

**1. Manual Review of References**

A manual review of references cited in the included studies was conducted to identify additional relevant literature that may not have been captured in the initial search.

**2. Examination of Related Systematic Reviews**

Systematic reviews that shared at least one study with the included reviews were examined to uncover further studies that could contribute valuable insights to this overview.

**3. Additional Records Identified**

Supplementary searches were performed across various platforms and methods, yielding the following:

Websites (n = 6):

1. National Institute for Health and Care Excellence. Medicines associated with dependence or withdrawal symptoms: safe prescribing and withdrawal management for adults [NG215]; 2022:1–41. <https://www.nice.org.uk/guidance/ng215>
2. Google Scholar <https://scholar.google.es/>
3. WHO: [http://www.who.int/en/](about:blank)
4. Health & Environmental Research Online (HERO) <https://hero.epa.gov/>
5. PROSPERO <https://www.crd.york.ac.uk/prospero/>
6. Crowley D, Delargy I, Bourke M, et al. For government of Irelanddepartment of health and children. Benzodiazepines: good practice guidelines for clinicians; 2002:1–28. <https://www.drugsandalcohol.ie/5349/>

Organizations (n = 9):

1. **MAGIC Evidence Ecosystem Foundation**

- **Referencia:** Agoritsas T, Kumbargere N, Callegari ES, Johansson M. **BE-SAFE: deprescription of benzodiazepine and sedative hypnotics (BSHs) in insomnia disorder.** MAGIC Evidence Ecosystem Foundation; 2023.
- **Enlace:** [Acceder](https://app.magicapp.org/#/guideline/jDePyL) (Accedido el 29 de diciembre de 2024).

1. **Benzodiazepine Action Working Group**

- **Referencia:** **Benzodiazepine Deprescribing Guidance**; 2022.
- **Enlace:** [Acceder](https://corxconsortium.org/wp-content/uploads/Benzo-Deprescribing.pdf) (Accedido en 2024).

1. **National Institute for Health and Care Excellence (NICE)**

- **Referencia:** **Medicines associated with dependence or withdrawal symptoms: safe prescribing and withdrawal management for adults [NG215]**; 2022:1–41.
- **Enlace:** [Acceder](https://www.nice.org.uk/guidance/ng215/resources/medicines-associated-with-dependence-or-withdrawal-symptoms-safe-prescribing-and-withdrawal-management-for-adults-pdf-66143776880581) (Accedido en septiembre de 2024).

1. **Royal Australian College of General Practitioners (RACGP)**

- **Referencia:** **Prescribing drugs of dependence in general practice, Part B—benzodiazepines.** Melbourne; 2015:1–85.
- **Enlace:** [Acceder](https://www.racgp.org.au/download/Documents/Guidelines/Addictive-drugs/Addictive-drugs-guide-B.pdf) (Accedido en septiembre de 2024).

1. **JPS Health Network**

- **Referencia:** **Guidelines for prescribing and tapering benzodiazepines in an outpatient setting**; 2014:1–11.
- **Enlace:** [Acceder](https://nmbon.sks.com/uploads/FileLinks/d7c0192068a54744b9cb11172053e46e/Guidelines_for_the_use_of_Benzo.pdf) (Accedido en septiembre de 2024).

1. **College of Psychiatry of Ireland**

- **Referencia:** **A consensus statement on the use of benzodiazepines in specialist mental health services.** EAP Position Paper; 2012:1–9.
- **Enlace:** [Acceder](http://www.drugsandalcohol.ie/23872/1/CPsychI_position_paper_on_benzodiazepimes_June_2012.sflb.pdf) (Accedido en septiembre de 2024).

1. **New Mexico Overdose Prevention and Pain Management Advisory Council**

- **Referencia:** **Guidelines for the use of benzodiazepines in the state of New Mexico**; 2018:1–7.
- **Enlace:** [Acceder](https://www.nmhealth.org/publication/view/guide/6327/#:%E2%88%BC:text=Long%2Dterm%20use%3A,or%20any%20mental%20health%20indication) (Accedido en septiembre de 2024).

1. **Haute Autorité de Santé (HAS)**

- **Referencia:** **Guidance leaflet–discontinuation of benzodiazepines and related medicinal products: procedure for the doctor providing outpatient treatment**; 2015:1–5.
- **Enlace:** [Acceder](https://www.has-sante.fr/upload/docs/application/pdf/2016-11/fiche_memo_arret_benzodiazepines_pour_mel_2015_06_16_en.pdf) (Accedido en septiembre de 2024).

1. **Ministry of Health Singapore**

- **Referencia:** **Prescribing of benzodiazepines**; 2008:1–57.
- **Enlace:** [Acceder](https://www.moh.gov.sg/docs/librariesprovider4/guidelines/cpg_prescribing-of-benzodiazepines.pdf) (Accedido en septiembre de 2024).

Citation Searching (n = 274): Citation searches were conducted to track references that cited key studies included in the overview.

| **Nro** | **Author/ Year** | **Reference** |
| --- | --- | --- |
| 1 | **Aharaz et al., 2021** | Aharaz A, Rasmussen JH, McNulty HBO, et al. A collaborative deprescribing intervention in a subacute medical outpatient clinic: A pilot randomized controlled trial. Metabolites. 2021;11(4) |
| 2 | **Ailabouni et al., 2019** | Ailabouni N, Mangin D, Nishtala PS. DEFEAT-polypharmacy: deprescribing anticholinergic and sedative medicines feasibility trial in residential aged care facilities. Int J Clin Pharm. 2019;41(1):167–178. |
| 3 | **Monzón-Kenneke et al., 2021** | Monzón-Kenneke M, Chiang P, Yao NA, Greg M. Pharmacist medication review: an integrated team approach to serve home-based primary care patients. PLoS One. 2021;16(5), e0252151. |
| 4 | **van der Meer et al., 2019** | van der Meer HG, Wouters H, Teichert M, et al. Feasibility, acceptability and potential effectiveness of an information technology-based, pharmacist-led intervention to prevent an increase in anticholinergic and sedative load among older community-dwelling individuals. Ther Adv Drug Safety. 2019;10:1–13. |
| 5 | **Campbell et al., 2019** | Campbell NL, Perkins AJ, Khan BA, et al. Deprescribing in the pharmacologic Management of Delirium: a randomized trial in the intensive care unit. J Am Geriatr Soc. 2019;67(4):695–702. |
| 6 | **Carr et al., 2019** | Carr F, Tian P, Chow J, et al. Deprescribing benzodiazepines among hospitalised older adults: quality improvement initiative. BMJ Open Qual. 2019;8(3), e000539. |
| 7 | **Feldman et al., 2019** | Feldman EA, Noviasky J, Ulen KR, et al. Targeted medication Deprescribing in the elderly: results of a pharmacist-driven procedure in a transitional care unit. Sr Care Pharm. 2019;34(10):678–686. |
| 8 | **Houlind et al., 2020** | Houlind MB, Andersen AL, Treldal C, et al. A collaborative medication review including deprescribing for older patients in an emergency department: A longitudinal feasibility study. J Clin Med. 2020;9(2) (no pagination). |
| 9 | **Mudge et al., 2016** | Mudge A, Radnedge K, Kasper K, et al. Effects of a pilot multidisciplinary clinic for frequent attending elderly patients on deprescribing. Australian Health Rev A Publicat Australian Hosp Associat. 2016;40(1):86–91. |
| 10 | **Shilpa et al., 2019** | Shilpa HSS, Kumar NN, Maheswari E, et al. Deprescribing of benzodiazepines and Z-drugs amongst the psychiatric patients of a tertiary care hospital. Asian J Psychiatr. 2019;44:189–194. |
| 11 | **Wilson et al., 2018** | Wilson MG, Lee TC, Hass A, et al. EMPOWERing hospitalized older adults to deprescribe sedative hypnotics: a pilot study. J Am Geriatr Soc. 2018;66(6):1186–1189. |
| 12 | **Lindsay et al., 2015** | Lindsay J, Dooley M, Martin J, et al. The development and evaluation of an oncological palliative care deprescribing guideline: the ‘OncPal deprescribing guideline’. Support Care Cancer. 2015;23(1):71–78. |
| 13 | **Martin et al., 2018** | Martin P, Tamblyn R, Benedetti A, et al. Effect of a pharmacist-led educational intervention on inappropriate medication prescriptions in older adults: the D-PRESCRIBE randomized clinical trial. JAMA. 2018;320(18):1889–1898. |
| 14 | **Tannenbaum et al., 2014** | Tannenbaum C, Martin P, Tamblyn R, et al. Reduction of inappropriate benzodiazepine prescriptions among older adults through direct patient education: the EMPOWER cluster randomized trial. JAMA Intern Med. 2014;174(6):890–898. |
| 15 | **Turner et al., 2021** | Turner JP, Sanyal C, Martin P, et al. Economic evaluation of sedative deprescribing in older adults by community pharmacists. J Gerontol. 2021;76(6):1061–1067. |
| 16 | **Lui et al., 2021** | Lui E, Wintemute K, Muraca M, et al. Pharmacist-led sedative-hypnotic deprescribing in team-based primary care practice. Canadian Pharmac J. 2021;154(4):278–284. |
| 17 | **Westbury et al., 2018** | Westbury JL, Gee P, Ling T, et al. RedUSe: reducing antipsychotic and benzodiazepine prescribing in residential aged care facilities. Med J Aust. 2018;208(9):398–403. |
| 18 | **Farrell et al., 2018** | Farrell B, Richardson L, Raman-Wilms L, et al. Self-efficacy for deprescribing: a survey for health care professionals using evidence-based deprescribing guidelines. Res Soc Administrat Pharm RSAP. 2018;14(1):18–25. |
| 19 | **Blenke et al., 2018** | Blenke AA, Van Marum RJ, Windsant-Van Den Tweel AMV, et al. Deprescribing in newly admitted psychogeriatric nursing facility patients. Consult Pharm. 2018;33(6):331–338. |
| 20 | **Whitman et al., 2018** | Whitman A, DeGregory K, Morris A, et al. Pharmacist-led medication assessment and deprescribing intervention for older adults with cancer and polypharmacy: a pilot study. Support Care Cancer. 2018;26(12):4105–4113. |
| 21 | **Ashton 1990** | Ashton CH, Rawlins MD, Tyrer SP. A double-blind placebo-controlled study of buspirone in diazepam withdrawal in chronic benzodiazepine users. *British Journal of Psychiatry* 1990;157:232-8. |
| 22 | **Baandrup 2016** | Baandrup L, Fagerlund B, Glenthoj B. Neurocognitive performance, subjective well-being, and psychosocial functioning after benzodiazepine withdrawal in patients with schizophrenia or bipolar disorder: a randomized clinical trial of add-on melatonin versus placebo. *European Archives of Psychiatry and Clinical Neuroscience* 2017;267(2):163-71. |
| 23 | **Cassano 1996** | Cassano GB, Petracca A, Borghi C, Chiorri S, Didoni G, Garreau M. A randomized, double-blind study of alpidem vs placebo in the prevention and treatment of benzodiazepine withdrawal syndrome. *European Psychiatry* 1996;11(2):93-9. |
| 24 | **Cialdella 2001** | Cialdella P, Boissel JP, Belon P, the ASTRHO group. Homeopathic specialties as a substitute for benzodiazepines: a double-blind vs. placebo study. *Thérapie* 2001;56:397-402. |
| 25 | **Di Costanzo 1992** | Di Costanzo E, Rovea A. The prophylaxis of benzodiazepine withdrawal syndrome in the elderly: the effectiveness of carbamazepine. A double-blind study vs. placebo. *Minerva Psichiatrica* 1992;33:301-4. |
| 26 | **Garfinkel 1999** | Garfinkel D, Zisapel N, Wainstein J, Laudon M. Facilitation of benzodiazepine discontinuation by melatonin: a new clinical approach. *Archives of Internal Medicine* 1999;159(20):2456-60. |
| 27 | **Gerra 1993** | Gerra G, Marcato A, Caccavari R, Fontanan-QQin C, Fortunato A, Zaimovic A, et al. Effectiveness of flumazenil (Ro 15-1788) in the treatment of benzodiazepine withdrawal. *Current Therapeutic Research - Clinical and Experimental* 1993;54(5):580-7. |
| 28 | **Gerra 2002** | Gerra G, Zaimovic A, Guisti M, Moi G, Brewer C. Intravenous flumazenil versus oxazepam tapering in the treatment of benzodiazepine withdrawal: a randomized, placebo-controlled study. *Addiction Biology* 2002;7:385-95. |
| 29 | **GlaxoSmithKline 2002** | [GlaxoSmithKline. The comparison of paroxetine and placebo on the symptoms emerging during the taper phase of a chronic benzodiazepine treatment: a randomised clinical trial of anxiety disorders. CSK - Clinical Study Register 2002. www.gsk-clinicalstudyregister.com.](http://www.gsk-clinicalstudyregister.com/) |
| 30 | **Hadley 2012** | Hadley SJ, Mandel FS, Schweizer E. Switching from long-term benzodiazepine therapy to pregabalin in patients with generalized anxiety disorder: a double-blind, placebo-controlled trial. *Journal of Psychopharmacology* 2012;26(4):461-70. |
| 31 | **Hantouche 1998** | Hantouche EG, Guelfi JD, Comet D. Discontinuation of long-term benzodiazepine use: double-blind controlled study of α-β L-aspartate magnesium versus placebo in 144 chronic users. *L'Encéphale* 1998;24:469-79. |
| 32 | **Harrison-Read 1996** | Harrison-Read PE, Tyrer P, Lawson C, Kader I, File SE. Flumazenil-precipitated panic and dysphoria in patients dependent on benzodiazepines: a possible aid to abstinence. *Journal of Psychopharmacology* 1996;10:89-97. |
| 33 | **Klein 1994** | Klein E, Colin V, Saksi K, Liron RH. Alprazolam withdrawal in patients with panic disorder and generalized anxiety disorder: a double-blind comparison. *American Journal of Psychiatry* 1994;151(12):1760-6. |
| 34 | **Kornowski 2002** | Kornowski J. The comparison between tianeptine and carbamazepine in benzodiazepines withdrawal syndrome. Psychiatria Polska 2002;6(Suppl):311-8. |
| 35 | **Lader 1987** | Lader M, Olajide D. A comparison of buspirone and placebo in relieving benzodiazepine withdrawal symptoms. *Journal of Clinical Psychopharmacology* 1987;7(1):11-5. |
| 36 | **Lader 1993** | Lader M, Farr I, Morton S. A comparison of alpidem and placebo in relieving benzodiazepine withdrawal symptoms. International Clinical Psychopharmacology 1993;8(1):31-6. |
| 37 | **Lecrubier 2005** | Lecrubier Y, Fessard N. Benzodiazepine discontinuation in chronic users: a double-blind trial of lithium gluconate vs placebo. *Annales Medico Psychologiques* 2005;163:24-9. |
| 38 | **Lemoine 2006** | Lemoine P, Kermadi I, Garcia-Acosta S, Garay RP, Dib M. Double-blind, comparative study of cyamemazine vs. bromazepam in the benzodiazepine withdrawal syndrome. Progress in Neuro-Psychopharmacology & Biological Psychiatry 2006;30(1):131-7. |
| 39 | **Mariani 2016** | Mariani JJ, Malcolm RJ, Mamczur AK, Choi JC, Brady R, Nunes E, et al. Pilot trial of gabapentin for the treatment of benzodiazepine abuse or dependence in methadone maintenance patients. American Journal of Drug and Alcohol Abuse 2016;42(3):333-40. [PUBMED: 26962719] |
| 40 | **Mercier-Guyon 2004** | Mercier-Guyon C, Chabannes JP, Saviuc P. The role of captodiamine in the withdrawal from long-term benzodiazepine treatment. *Current Medical Research and Opinion* 2004;20(9):1347-55. |
| 41 | **Morton 1995** | Morton S, Lader M. Buspirone treatment as an aid to benzodiazepine withdrawal. Journal of Psychopharmacology 1995;9(4):331-5. |
| 42 | **Nakao 2006** | Nakao M, Takeuchi T, Nomura K, Teramoto T, Yano E. Clinical application of paroxetine for chronic benzodiazepine users at an internal medicine clinic. Therapeutic Research 2006;27(5):859-67. |
| 43 | **Pat-Horenczyk 1998** | Pat-Horenczyk R, Hacohen D, Herer P, Lavie P. The eQects of substituting zopiclone in withdrawal from chronic use of benzodiazepine hypnotics. Psychopharmacology 1998;140(4):450-7. [DOI: 10.1007/s002130050789] |
| 44 | **Peles 2007** | Peles E, Hetzroni T, Bar-Hamburger R, Adelson M, Schreiber S. Melatonin for perceived sleep disturbances associated with benzodiazepine withdrawal among patients in methadone maintenance treatment: a double-blind randomized clinical trial. Addiction 2007;102(12):1947-53. |
| 45 | **Rickels 1999** | Rickels K, Schweizer E, Garcia-Espana F, Case G, DeMartinis N, Greenblatt D. Trazodone and valproate in patients discontinuing long-term benzodiazepine therapy: effects on withdrawal symptoms and taper outcome. *Psychopharmacology* 1999;141(1):1-5. |
| 46 | **Rickels 2000** | Rickels K, Schweizer E, Garcia Espana F, Case, G, DeMartinis N, Greenblatt D. Trazodone and valproate in patients discontinuing long-term benzodiazepine therapy: eQects on withdrawal symptoms and taper outcome. Psychopharmacology 1999;141(1):1-5. |
| 47 | **Romach 1998** | Romach MK, Kaplan HL, Busto UE, Somer G, Sellers EM. A controlled trial of ondansetron, a 5-HT3 antagonist, in benzodiazepine discontinuation. Journal of Clinical Psychopharmacology 1998;18(2):121-31. |
| 48 | **Rynn 2003** | Rynn M, Garcia-Espana F, Greenblatt DJ, Mandos LA, Schweizer E, Rickels K. Imipramine and buspirone in patients with panic disorder who are discontinuing long-term benzodiazepine therapy. Journal of Clinical Psychopharmacology 2003;23(5):505-8. |
| 49 | **Saul 1989** | Saul PA, Korlipara K, Presley P. A randomised, multicentre, double-blind, comparison of atenolol and placebo in the control of benzodiazepine withdrawal symptoms. Acta Therapeutica 1989;15(2):117-23. |
| 50 | **Schweizer 1991** | Schweizer E, Rickels, K, Case WG, Greenblatt DJ. Carbamazepine treatment in patients discontinuing long-term benzodiazepine therapy. EQects on withdrawal severity and outcome. Archives of General Psychiatry 1991;48(5):448-52. |
| 51 | **Schweizer 1995** | Schweizer E, Case WG, Garcia-Espana F, Greenblatt DJ, Rickels K. Progesterone co-administration in patients discontinuing long-term benzodiazepine therapy: effects on withdrawal severity and outcome. *Psychopharmacology* 1995;117(4):424-9. |
| 52 | **Tyrer 1981** | Tyrer P, Rutherford D, Huggett T. Benzodiazepine withdrawal symptoms and propranolol. *Lancet* 1981;1(8221):520-2. |
| 53 | **Tyrer 1996** | Tyrer P, Ferguson B, Hallstrom C, Michie M, Tyrer S, Cooper S, et al. A controlled trial of dothiepin and placebo in treating benzodiazepine withdrawal symptoms. British Journal of Psychiatry 1996;168(4):457-61. |
| 54 | **Udelman 1990** | Udelman HD, Udelman DL. Concurrent use of buspirone in anxious patients during withdrawal from alprazolam therapy. Journal of Clinical Psychiatry 1990;51 Suppl:46-50. |
| 55 | **Vissers 2007** | Vissers FH, Knipschild PG, Crebolder HF. Is melatonin helpful in stopping the long-term use of hypnotics? A discontinuation trial. Pharmacy World & Science 2007;29(6):641-6. [DOI: 10.1007/s11096-007-9118-y] |
| 56 | **Vorma 2011** | Vorma H, Katila H. Effect of valproate on benzodiazepine withdrawal severity in opioid-dependent subjects: a pilot study. *Heroin Addiction and Related Clinical Problems* 2011;13(1):15-20. |
| 57 | **Zhang 2013** | Zhang H, Jiang X, Ma M, Zhang J. A control study on treatment for benzodiazepine dependence with trazodone. *Chinese Journal of Contemporary Neurology and Neurosurgery* 2013;13(5):411-5. |
| 58 | **Zitman 2001** | Zitman FG, Couvee JE. Chronic benzodiazepine use in general practice patients with depression: an evaluation of controlled treatment and taper-ex protocol on behalf of the Dutch Chronic Benzodiazepine Working Group. *British Journal of Psychiatry* 2001;178:317-24. |
| 59 | **Eleftheriou et al., 2023** | Consensus panel recommendations for the pharmacological management of pregnant women with depressive disorders. Int J Environ Res Publ Health. 2023;20(16):6565. [DOI](https://doi.org/10.3390/ijerph2016 6565) |
| 60 | **Ilješ et al., 2023** | Recommendations for treatment of unipolar depressive disorder. Slov Med J. 2023:1–6. DOI |
| 61 | **Miller et al., 2023** | Treatment and management of mental health conditions during pregnancy and postpartum: ACOG clinical practice guideline No. 5. Obstet Gynecol. 2023;141(6):1262–1288. DOI |
| 62 | **Mula et al., 2022** | ILAE clinical practice recommendations for the medical treatment of depression in adults with epilepsy. Epilepsia. 2022;63(2):316–334. DOI |
| 63 | **Voytenko et al., 2018** | Evidence-Based practice guideline for the treatment of adult patients with depressive disorders. Part I: psychiatric management. Psychiatr i Psychol Klin. 2018;18(3):234–241. DOI |
| 64 | **Piotrowski et al., 2017** | Guidelines of the polish psychiatric association–wroclaw division, the polish society of family medicine and the college of family physicians in Poland for diagnosis and treatment of depressive disorders in primary health care. Fam Med Prim Care Rev. 2017;(3):335–346. DOI |
| 65 | **Kennedy et al., 2016** | CANMAT 2016 clinical guidelines for the management of adults with major depressive disorder: section 3. Pharmacological treatments. Can J Psychiatr. 2016;61(9):540–560. DOI |
| 66 | **Bauer et al., 2013** | World Federation of Societies of Biological Psychiatry (WFSBP) guidelines for biological treatment of unipolar depressive disorders, part 1: update 2013 on the acute and continuation treatment of unipolar depressive disorders. World J Biol Psychiatr. 2013;14(5):334–385. DOI |
| 67 | **Bauer et al., 2015** | WFSBP guidelines for biological treatment of unipolar depressive disorders. Part 2: maintenance treatment of major depressive disorder-update 2015. World J Biol Psychiatr. 2015;16(2):76–95. DOI |
| 68 | **Austin et al., 2013** | Detection and management of mood disorders in the maternity setting: the Australian Clinical Practice Guidelines. Women Birth. 2013;26(1):29. DOI |
| 69 | **Chua et al., 2012** | Ministry of Health clinical practice guidelines: depression. Singap Med J. 2012;53(2):137–144. |
| 70 | **Malhi et al., 2009** | Clinical practice recommendations for depression. Acta Psychiatr Scand. 2009;119:8–26. DOI |
| 71 | **Fleck et al., 2009** | Review of the guidelines of the Brazilian Medical Association for the treatment of depression (Full version). Br J Psychiatry. 2009;31:S7–S17. DOI |
| 72 | **Anderson 2001** | Evidence-based guidelines for treating depressive disorders with antidepressants: a revision of the 1993 British Association for Psychopharmacology guidelines. J Psychopharmacol. 2000;14(1):3–20. DOI |
| 73 | **Conn 2006** | National guidelines for seniors’ mental health: the assessment and treatment of mental health issues in long-term care homes (focus on mood and behaviour symptoms). Toronto, ON: Canadian Coalition for Seniors’ Mental Health; 2006:1–56. PDF |
| 74 | **Rosenbluth 2012** | The Canadian Network for Mood and Anxiety Treatments (CANMAT) task force recommendations for the management of patients with mood disorders and comorbid personality disorders. Ann Clin Psychiatr. 2012;24(1):56–68 |
| 75 | **Dodd 2011** | A consensus statement for safety monitoring guidelines of treatments for major depressive disorder. Aust N Z J Psychiatry. 2011;45(9):712–725. DOI |
| 76 | **Motohashi 2008** | Revised psychopharmacological algorithms for the treatment of mood disorders in Japan. Int J Psychiatr Clin Pract. 2008;12(1):11–18. DOI |
| 77 | **Romeijnders 2005** | [Summary of the standard ’depressive disorder’ (first revision) of the Dutch society of general practitioners]. Ned Tijdschr Geneeskd. 2005;149:523–527 |
| 78 | **Crismon 1999** | The Texas medication algorithm project: report of the Texas consensus conference panel on medication treatment of major depressive disorder. JClin Psychiatr. 1999;60(3):142–156. DOI |
| 79 | **Doctors of B.C 2013** | Major depressive disorder in adults: diagnosis & management; 2013:1–6. PDF (Accessed September 2023) |
| 80 | **Ministry of Health Malaysia 2019** | Management of major depressive disorder. 2nd ed.; 2019:1–66. PDFCPG_Management_Major_Depressive_Disorder(Second_Edition).pdf) (Accessed September 2023) |
| 81 | **Trangle 2016** | Institute for clinical system improvement. Adult depression in primary care: healthcare guideline. Bloomington, MN; 2016:1–131. PDF (Accessed September 2023) |
| 82 | **Scottish Intercollegiate Guidelines Network 2012** | Sign 127: management of perinatal mood disorders; 2012:1–47. PDF (Accessed September 2023) |
| 83 | **Claassen 2022** | [NHG-Standaard depressie (M44)]; 2022:1–113. Link (Accessed September 2023) |
| 84 | **Finnish Medical Society Duocedim 2023** | Depression: good medical practice. Helsinki; 2023. Link (Accessed September 2023) |
| 85 | **Schaffer 2012** | The Canadian Network for Mood and Anxiety Treatments (CANMAT) task force recommendations for the management of patients with mood disorders and comorbid anxiety disorders. Ann Clin Psychiatr. 2012;24(1):6–22 |
| 86 | **Fuchs 2000** | Guidelines for the treatment of depression. Rev Med Liege. 2000;55(5):389–394 |
| 87 | **Andrews 2018** | Royal Australian and New Zealand College of Psychiatrists clinical practice guidelines for the treatment of panic disorder, social anxiety disorder and generalised anxiety disorder. Aust N Z J Psychiatr. 2018;52(12):1109–1172. DOI |
| 88 | **Subramanyam 2018** | Clinical practice guidelines for geriatric anxiety disorders. Indian J Psychiatr. 2018;60(Suppl 3):S371–S382. DOI |
| 89 | **Gautam 2017** | Clinical practice guidelines for the management of generalised anxiety disorder (GAD) and panic disorder (PD). Indian J Psychiatr. 2017;59(Suppl 1):S67–S73. DOI |
| 90 | **Bandelow 2022** | The German Guidelines for the treatment of anxiety disorders: first revision. Eur Arch Psychiatr Clin Neurosci. 2022;272(4):571–582. DOI |
| 91 | **Katzman 2014** | Canadian clinical practice guidelines for the management of anxiety, posttraumatic stress and obsessive-compulsive disorders. BMC Psychiatry. 2014;14:1–83. DOI |
| 92 | **Baldwin 2014** | Evidence-based pharmacological treatment of anxiety disorders, PTSD, and OCD: a revision of the 2005 guidelines from the British Association for Psychopharmacology. J Psychopharmacol. 2014;28(5):403–439. DOI |
| 93 | **NICE 2011** | Generalised anxiety disorder and panic disorder in adults: management. NICE Clinical Guideline 113; 2011:1–47. Link (Accessed September 2023) |
| 94 | **Guideline working group 2008** | Clinical Practice Guidelines in the NHS. UETS N◦ 2006/10. Link (Accessed September 2023) |
| 95 | **Heggie 2018** | Magellan’s clinical practice guideline for the assessment and treatment of generalized anxiety disorder in adults; 2018:1–62. Link (Accessed September 2023) |
| 96 | **Finnish Medical Society Duocedim 2019** | Anxiety disorders. Current care guidelines; 2019. Link (Accessed September 2023) |
| 97 | **NHG-Working Group 2019** | [NHG-Standard anxiety (M62)]; 2019:1–73. Link (Accessed September 2023) |
| 98 | **Haute Autorité de Santé 2017** | [Acts and services for long-term conditions: serious anxiety disorders]; 2017. Link (Accessed September 2023) |
| 99 | **Brackett 2019** | Dartmouth-hitchcock. Clinical practice guideline: management of anxiety in adults in primary care; 2019:1–7. Link (Accessed September 2023) |
| 100 | **Linden 2013** | The best next drug in the course of generalized anxiety disorders: the “PN-GAD-algorithm”. Int J Psychiatr Clin Pract. 2013;17(2):78–89. DOI |
| 101 | **Abejuela 2016** | The psychopharmacology algorithm project at the Harvard South Shore Program: an algorithm for generalized anxiety disorder. Harv Rev Psychiatr. 2016;24(4):243-256. DOI |
| 102 | **Davidson 2010** | A psychopharmacological treatment algorithm for generalised anxiety disorder (GAD). J Psychopharmacol. 2010;24(1):3–26. DOI |
| 103 | **Allgulander 2003** | WCA recommendations for the long-term treatment of generalized anxiety disorder. CNS Spectr. 2003;8(S1):53–61. |
| 104 | **Bandelow 2023** | World Federation of Societies of Biological Psychiatry (WFSBP) guidelines for treatment of anxiety, obsessive-compulsive, and posttraumatic stress disorders–Version 3. Part I: anxiety disorders. World J Biol Psychiatr. 2023;24(2):79–117. DOI |
| 105 | **Yoon 2018** | Korean guidelines for the pharmacological treatment of social anxiety disorder: initial treatment strategies. Psychiatry Investig. 2018;15(2):147–155. DOI |
| 106 | **Levitan 2011** | Guidelines of the Brazilian Medical Association for the treatment of social anxiety disorder. Br J Psychiatry. 2011;33:292–302. DOI |
| 107 | **Stein 2010** | A 2010 evidence-based algorithm for the pharmacotherapy of social anxiety disorder. Curr Psychiatr Rep. 2010;12:471–477. DOI |
| 108 | **Van Ameringen 2003** | WCA recommendations for the long-term treatment of social phobia. CNS Spectr. 2003;8(S1):40–52. |
| 109 | **Ballenger 1998** | Consensus statement on social anxiety disorder from the international consensus group on depression and anxiety. J Clin Psychiatr. 1998;59:54–60. |
| 110 | **Stein 2001** | Pharmacotherapy of social anxiety disorder: an algorithm for primary care–2001. Prim Care Psychiatr. 2001;7(3):107–110. DOI |
| 111 | **National Collaborating Centre 2013** | Social anxiety disorder: the NICE guideline on recognition, assessment and treatment. British Psychological Society & Royal College of Psychiatrists; 2013:1–320. ISBN-: 978-1-909726-03-1. PDF |
| 112 | **Stein 2009** | Practice guideline for the treatment of patients with panic disorder (2nd ed.). Am Psychiatr Assoc. 2009;166(2):1–90. DOI |
| 113 | **Pollack 2003** | WCA recommendations for the long-term treatment of panic disorder. CNS Spectr. 2003;8(S1):17–30. |
| 114 | **Roy-Byrne 1998** | Pharmacotherapy of panic disorder: proposed guidelines for the family physician. J Am Board Fam Pract. 1998;11(4):282–290. |
| 115 | **Ballenger 1998** | Consensus statement on panic disorder from the international consensus group on depression and anxiety. J Clin Psychiatr. 1998;59:47–54. |
| 116 | **Bandelow 2023** | World Federation of Societies of Biological Psychiatry (WFSBP) guidelines for treatment of anxiety, obsessive-compulsive and posttraumatic stress disorders–Version 3. Part II: OCD and PTSD. World J Biol Psychiatr. 2023;24(2):118–134. DOI |
| 117 | **Reddy 2017** | Clinical practice guidelines for obsessive-compulsive disorder. Indian J Psychiatr. 2017;59(S1):S74–S90. DOI |
| 118 | **de Oliveira 2023** | Brazilian Research Consortium on Obsessive-Compulsive Spectrum Disorders guidelines for the treatment of adult obsessive-compulsive disorder. Part I: pharmacological treatment. Br J Psychiatry. 2023;45:146–161. DOI |
| 119 | **Koran 2013** | Guideline watch (March 2013): practice guideline for the treatment of patients with obsessive-compulsive disorder. Arlington, TX, USA: American Psychiatric Association Practice Guidelines; 2013:1–22. |
| 120 | **Greist 2003** | WCA recommendations for the long-term treatment of obsessive-compulsive disorder in adults. CNS Spectr. 2003;8(S1):7–16. DOI |
| 121 | **Stein 2012** | A 2012 evidence-based algorithm for the pharmacotherapy for obsessive-compulsive disorder. Curr Psychiatr Rep. 2012;14:211–219. DOI |
| 122 | **Forbes 2007** | Australian guidelines for the treatment of adults with acute stress disorder and post-traumatic stress disorder. Aust N Z J Psychiatr. 2007;41(8):637–648. DOI |
| 123 | **Ursano 2010** | Practice guideline for the treatment of patients with acute stress disorder and posttraumatic stress disorder. Washington, DC. USA: American Psychiatric Association Practice Guidelines; 2010:1–95. |
| 124 | **Foa 2000** | Guidelines for treatment of PTSD. J Trauma Stress. 2000;13(4):539–588. DOI |
| 125 | **Stein 2003** | WCA Recommendations for the long-term treatment of posttraumatic stress disorder. CNS Spectr. 2003;8(S1):31–39. DOI |
| 126 | **National Center for PTSD 2013** | Helping patients taper from benzodiazepines; 2013:1–2. PDF (Accessed September 2023) |
| 127 | **Watson 2023** | Alliance for sleep clinical practice guideline on switching or deprescribing hypnotic medications for insomnia. J Clin Med. 2023;12(7):1–22. DOI |
| 128 | **Wichniak 2023** | Treatment of insomnia in older adults. Recommendations of the polish sleep research society, polish society of family medicine and the polish psychiatric association. Psychiatr Pol. 2023;57(3). DOI |
| 129 | **Mysliwiec 2020** | The management of chronic insomnia disorder and obstructive sleep apnea: synopsis of the 2019 US Department of Veterans Affairs and US Department of Defense clinical practice guidelines. Ann Intern Med. 2020;172(5):325–336. DOI |
| 130 | **Palagini 2020** | Expert opinions and consensus recommendations for the evaluation and management of insomnia in clinical practice: joint statements of five Italian scientific societies. Front Psychiatr. 2020;11:558. DOI |
| 131 | **Choi 2020** | Korean clinical practice guideline for the diagnosis and treatment of insomnia in adults. Psychiatry Investig. 2020;17(11):1048–1059. DOI |
| 132 | **Wilson 2019** | British Association for Psychopharmacology consensus statement on evidence-based treatment of insomnia, parasomnias and circadian rhythm disorders: an update. J Psychopharmacol. 2019;33(8):923–947. DOI |
| 133 | **Praharaj 2018** | Clinical practice guideline on management of sleep disorders in the elderly. Indian J Psychiatr. 2018;60(S3):S383–S396. DOI |
| 134 | **Pottie 2018** | Deprescribing benzodiazepine receptor agonists: evidence-based clinical practice guideline. Can Fam Physician. 2018;64(5):339–351. Full Text |
| 135 | **Sateia 2017** | Clinical practice guideline for the pharmacologic treatment of chronic insomnia in adults: an American Academy of Sleep Medicine clinical practice guideline. J Clin Sleep Med. 2017;13(2):307–349. DOI |
| 136 | **Riemann 2017** | European guideline for the diagnosis and treatment of insomnia. J Sleep Res. 2017;26(6):675–700. DOI |
| 137 | **Gupta 2017** | Clinical practice guidelines for sleep disorders. Indian J Psychiatr. 2017;59(S1):S116–S138. DOI |
| 138 | **Qaseem 2016** | Clinical Guidelines Committee of the American College of Physicians. Management of chronic insomnia disorder in adults: a clinical practice guideline from the American College of Physicians. Ann Intern Med. 2016;165(2):125–133. DOI |
| 139 | **Medina-Chávez 2014** | Clinical practice guideline. Diagnosis and treatment of insomnia in the elderly. Rev Méd Inst Mex Seguro Soc. 2014;52(1):108–119. |
| 140 | **Pinto 2010** | New guidelines for diagnosis and treatment of insomnia. Arq Neuropsiquiatr. 2010;68(4):666–675. |
| 141 | **Mayer 2009** | S3-Guideline. [Non restorative sleep⁄ sleep disorders. German Sleep Society]. Somnologie. 2009;13(S1):4–160. DOI |
| 142 | **Bloom 2009** | Evidence-based recommendations for the assessment and management of sleep disorders in older persons. J Am Geriatr Soc. 2009;57(5):761–789. DOI |
| 143 | **Terzano 2005** | Insomnia in general practice: a consensus report produced by sleep specialists and primary-care physicians in Italy. Clin Drug Invest. 2005;25:745–764. DOI |
| 144 | **Estivill 2003** | Consensus on drug treatment, definition and diagnosis for insomnia. Clin Drug Invest. 2003;23(6):351–385. DOI |
| 145 | **Roth 2001** | Consensus for the pharmacological management of insomnia in the new millennium. Int J Clin Pract. 2001;55(1):42–52. |
| 146 | **Yakabowich 1992** | Hypnotics in the elderly: appropriate usage guidelines. J Geriatr Drug Ther. 1992;6(3):5–21. DOI |
| 147 | **Alberta Doctors 2015** | Towards optimized practice: assessment to management of adult insomnia; 2015:1–35. Link |
| 148 | **Damen-van Beek 2015** | [The NHG guideline ’Sleep problems and sleeping pills’]. Ned Tijdschr Geneeskd. 2015; 159:A8679. |
| 149 | **Finnish Medical Society Duocedim 2023** | Insomnia. Valid treatment recommendation; 2023. Enlace. Accessed September 2023. |
| 150 | **Cloetens 2018** | First-line treatment of sleep disorders and insomnia in adults; 2018:1–10. [Enlace](https://cdn.nimbu.io/s/yba55wt/assets/WG Sleep disorders.pdf). Accessed September 2023. |
| 151 | **Agoritsas 2023** | BE-SAFE: deprescription of benzodiazepine and sedative hypnotics (BSHs) in insomnia disorder. MAGIC Evidence Ecosystem Foundation; 2023. Enlace. Accessed December 29, 2023. |
| 152 | **Benzodiazepine action working group 2022** | Benzodiazepine Deprescribing Guidance; 2022. Enlace. |
| 153 | **National Institute for Health and Care Excellence 2022** | Medicines associated with dependence or withdrawal symptoms: safe prescribing and withdrawal management for adults [NG215]; 2022:1–41. Enlace. Accessed September 2023. |
| 154 | **Sparks 2022** | Benzodiazepine and Z-drug safety guideline; 2022:1–26. Enlace. Accessed September 2023. |
| 155 | **Barry 2021** | Guidance on appropriate prescribing of benzodiazepines and Z-drugs (BZRA) for the treatment of anxiety and insomnia; 2021:1–48. Enlace. Accessed 2023. |
| 156 | **Conn 2020** | Canadian guidelines on benzodiazepine receptor agonist use disorder among older adults. Can Geriatr J. 2020;23(1):116–122. DOI. |
| 157 | **Amanti 2018** | Benzodiazepine taper guidelines for older adults in an inpatient geiatric/psychiatric unit. Dissertation. University of Arizona; 2018:1–97. Enlace. Accessed 2023. |
| 158 | **New Mexico Overdose Prevention and Pain Management Advisory Council 2018** | Guidelines for the use of benzodiazepines in the state of New Mexico; 2018:1–7. Enlace. Accessed September 2023. |
| 159 | **Royal Australian College of General Practitioners 2015** | Prescribing drugs of dependence in general practice, Part B—benzodiazepines. Melbourne; 2015:1–85. Enlace. Accessed September 2023. |
| 160 | **Haute Autorite de Sante 2015** | Guidance leaflet–discontinuation of benzodiazepines and related medicinal products: procedure for the doctor providing outpatient treatment; 2015:1–5. Enlace. Accessed September 2023. |
| 161 | **JPS Health Network 2014** | Guidelines for prescribing and tapering benzodiazepines in an outpatient setting; 2014:1–11. Enlace. Accessed September 2023. |
| 162 | **College of Psychiatry of Ireland 2012** | A consensus statement on the use of benzodiazepines in specialist mental health services. EAP Position Paper; 2012:1–9. Enlace. Accessed September 2023. |
| 163 | **Lee 2008** | For Ministry of Health Singapore. Prescribing of benzodiazepines; 2008:1–57. Enlace. Accessed September 2023. |
| 164 | **Crowley 2002** | Benzodiazepines: good practice guidelines for clinicians; 2002:1–28. Enlace. Accessed September 2023. |
| 165 | **Baillargeon 2003** | Discontinuation of benzodiazepines among older insomniac adults treated with cognitive-behavioural therapy combined with gradual tapering: a randomized trial. CMAJ 2003; 169: 1015–20. |
| 166 | **Cardinali 2002** | A double blind–placebo controlled study on melatonin efficacy to reduce anxiolytic benzodiazepine use in the elderly. Neuro Endocrinol Lett 2002; 23: 55–60. |
| 167 | **Giblin 1983** | Sleep without drugs. J R Coll Gen Pract 1983; 33: 628–33. |
| 168 | **Habraken 1997** | Gradual withdrawal from benzodiazepines in residents of homes for the elderly: experience and suggestions for future research. Eur J Clin Pharmacol 1997; 51: 355–8. |
| 169 | **Morin 2004** | Randomized clinical trial of supervised tapering and cognitive behavior therapy to facilitate benzodiazepine discontinuation in older adults with chronic insomnia. Am J Psychiatry 2004; 161: 332–42. |
| 170 | **Petrovic 2002** | Fast withdrawal from benzodiazepines in geriatric inpatients: a randomised double-blind, placebo-controlled trial. Eur J Clin Pharmacol 2002; 57: 759–64. |
| 171 | **Salonoja 2010** | One-time counselling decreases the use of benzodiazepines and related drugs among community-dwelling older persons. Age Ageing 2010; 39: 313–9. |
| 172 | **Tham 1989** | Temazepam withdrawal in elderly hospitalized patients – a double-blind randomized trial comparing abrupt versus gradual withdrawal. Ir J Med Sci 1989; 158: 294–9. |
| 173 | **Velert Vila 2011** | Intervención Farmacéutica para la Adecuación de la Prescripción de Benzodiazepinas en Pacientes Mayores. Cardinal Herrera University, 2011. |
| 174 | **Velert Vila 2012** | Adecuación de la utilización de benzodiazepinas en ancianos desde la oficina de farmacia. Un estudio de colaboración médicofarmacéutico. Aten Primaria 2012; 44: 402–10. |
| 175 | **Avorn 1992** | A randomized trial of a program to reduce the use of psychoactive drugs in nursing homes. N Engl J Med 1992; 327: 168–73. |
| 176 | **Crotty 2004** | An outreach intervention to implement evidence based practice in residential care: a randomized controlled trial. BMC Health Serv Res 2004; 4:6. |
| 177 | **Pit 2007** | A Quality Use of Medicines program for general practitioners and older people: a cluster randomised controlled trial. Med J Aust 2007; 187: 23–30. |
| 178 | **Rikala 2011** | The effects of medication assessment on psychotropic drug use in the community-dwelling elderly. Int Psychogeriatr 2011; 23: 473–84. |
| 179 | **Roberts 2001** | Outcomes of a randomized controlled trial of a clinical pharmacy intervention in 52 nursing homes. Br J Clin Pharmacol 2001; 51: 257–65. |
| 180 | **Strikwerda 1994** | Drug therapy in a nursing home; favorable effect of feedback by the pharmacist on family physician’s prescribing behavior. Ned Tijdschr Geneeskd 1994; 138: 1770–4. |
| 181 | **Heather N., 2004** | Heather N., Bowie A., Ashton H., McAvoy B., Spencer I., Brodie J. et al. Randomised controlled trial of two brief interventions against long-term benzodiazepine use: outcome of intervention. Addict Res Theory 2004; 12:141–54. |
| 182 | **Bashir K., 1994** | Bashir K., King M., Ashworth M. Controlled evaluation of brief intervention by general practitioners to reduce chronic use of benzodiazepines. Br J Gen Pract 1994; 44:408–12. |
| 183 | **Cormack M. A., 1994** | Cormack M. A., Sweeney K. G., Hughes-Jones H., Foot G. A. Evaluation of an easy, cost-effective strategy for cutting benzodiazepine use in general practice. Br J Gen Pract 1994; 44: 5–8. |
| 184 | **Vicens C., 2006** | Vicens C., Fiol F., Llobera J., Campoamor F., Mateu C., Alegret S. et al. Withdrawal from long-term benzodiazepine use: randomised trial in family practice. Br J Gen Pract 2006; 56: 958–63. |
| 185 | **Kuntz J. L., 2019** | Kuntz J. L., Kouch L., Christian D., Hu W., Peterson P. L. Patient education and pharmacist consultation influence on nonbenzodiazepine sedative medication deprescribing success for older adults. Perm J 2019; 23:18–61. |
| 186 | **Navy H. J., 2018** | Navy H. J., Weffald L., Delate T., Patel R. J., Dugan J. P. Clinical pharmacist intervention to engage older adults in reducing use of alprazolam. Consult Pharm 2018; 33:711–22. |
| 187 | **Vicens C., 2014** | Vicens C., Bejarano F., Sempere E., Mateu C., Fiol F., Socias I. et al. Comparative efficacy of two interventions to discontinue long-term benzodiazepine use: cluster randomised controlled trial in primary care. Br J Psychiatry 2014; 204:471–9. |
| 188 | **Ashworth 2021** | Ashworth N, Kain N, Wiebe D, et al. Reducing prescribing of benzodiazepines in older adults: a comparison of four physician-focused interventions by a medical regulatory authority. BMC Fam Pract. 2021;22(1):68. https://doi.org/10.1186/ s12875-021-01415-x. 24. |
| 189 | **Evrard 2020** | Evrard P, Henrard S, Foulon V, et al. Benzodiazepine use and Deprescribing in Belgian nursing homes: results from the COMEON study. J Am Geriatr Soc. 2020;68(12):2768–77. https://doi. org/10.1111/jgs.16751. |
| 190 | **Navy 2018** | Navy HJ, Weffald L, Delate T, et al. Clinical pharmacist intervention to engage older adults in reducing use of alprazolam. Consult Pharm. 2018;33(12):711–22. https://doi.org/10.4140/TCP.n.2018. 711. |
| 191 | **Roberts 2001** | Roberts MS, Stokes JA, King MA, et al. Outcomes of a randomized controlled trial of a clinical pharmacy intervention in 52 nursing homes. Br J Clin Pharmacol. 2001;51(3):257–65. https:// doi.org/10.1046/j.1365-2125.2001.00347.x. |
| 192 | **Martin 2017** | P. Martin, C. Tannenbaum. Use of the EMPOWER brochure to deprescribe sedative-hypnotic drugs in older adults with mild cognitive impairment. BMC Geriatr. 2017;17:37. |
| 193 | **Gnjidic 2019** | D. Gnjidic, H. Ong, C. Leung, et al. The impact of in-hospital patient-education intervention on older people’s attitudes and intention to have their benzodiazepines deprescribed: a feasibility study. Ther Adv Drug Saf. 2019;10:2042098618816562. |
| 194 | **Hagen 2005** | B.F. Hagen, C. Armstrong-Esther, et al. Neuroleptic and benzodiazepine use in nursing homes: characteristics and changes following pharmacist intervention. Int Psychogeriatr. 2005;17(4):631–652. |
| 195 | **Morgan 2002** | J.D. Morgan, D.J. Wright, H. Chrystyn. Pharmacoeconomic evaluation of a patient education letter aimed at reducing long-term prescribing of benzodiazepines. Pharm World Sci. 2002;24(6):231–235. |
| 196 | **Luchen 2019** | G.G. Luchen, E.S. Prohaska, J.F. Ruisinger, et al. Impact of community pharmacist intervention on concurrent benzodiazepine and opioid prescribing patterns. J Am Pharm Assoc. 2019;59(2):238–242. |
| 197 | **Shayegani 2018** | R. Shayegani, P. Panneman, K. Van Dyke, et al. Reducing co-prescriptions of benzodiazepines and opioids in a veteran population. Am J Manag Care. 2018;24(8):e265–e269. |
| 198 | **Harden 2015** | P. Harden, S. Ahmed, K. Ang, et al. Clinical implications of tapering chronic opioids in a veteran population. Pain Med. 2015;16(10):1975–1981. |
| 199 | **Geka 2019** | M. Geka, K. Inoue, et al. Effectiveness of pharmacist-convened multidisciplinary clinical team meetings in promoting appropriate benzodiazepine receptor agonist use. Yakugaku Zasshi. 2019;139(6):931–937. |
| 200 | **Furbish 2017** | S.M.L. Furbish, M.E. Kroehl, D.F. Loeb, et al. A pharmacist-physician intervention for reducing benzodiazepine prescribing among older adults. J Am Geriatr Soc. 2017;65(1):70–76. |
| 201 | **Westbury 2010** | J. Westbury, S. Jackson, P. Gee, et al. An effective approach to decrease antipsychotic and benzodiazepine use in nursing homes: the RedUSe project. Int Psychogeriatr. 2010;22(1):26–36. |
| 202 | **Darchuk 2010** | K.M. Darchuk, C.O. Townsend, J.D. Rome, et al. Longitudinal treatment outcomes for geriatric patients with chronic non-cancer pain at an interdisciplinary pain rehabilitation program. Pain Med. 2010;11(9):1352–1364. |
| 203 | **Hopkins 1982** | Hopkins DR, Sethi KB, Mucklow JC. Benzodiazepine withdrawal in general practice. J R Coll Gen Pract 1982;32(245):758-62. |
| 204 | **Schweizer 1990** | Schweizer E, Rickels K, Case WG, Greenblatt DJ. Long-term therapeutic use of benzodiazepines. II. Effects of gradual taper. Arch Gen Psychiatry 1990;47(10):908-15. |
| 205 | **Tyrer 1983** | Tyrer P, Owen R, Dawling S. Gradual withdrawal of diazepam after long-term therapy. Lancet 1983;1(8339):1402-6 |
| 206 | **Schweizer 1998** | Schweizer E, Rickels K, Case WG, Greenblatt DJ. Long-term therapeutic use of benzodiazepines. Psychol Med 1998;28(3):713-20 |
| 207 | **Couvee 2002** | Couvee JE, Timmermans MA, Zitman FG; Dutch Chronic Benzodiazepine Working Group. The long-term outcome of a benzodiazepine discontinuation programme in depressed outpatients. J Affect Disord 2002;70:133-41 |
| 208 | **Morin 2004** | Morin CM, Bastien C, Guay B, et al. Randomized clinical trial of supervised tapering and cognitive behavior therapy to facilitate benzodiazepine discontinuation in older adults with chronic insomnia. Am J Psychiatry 2004;161(2):332-42 |
| 209 | **Morin 2005** | Morin CM, Bélanger L, Bastien C, Vallières A. Long-term outcome after discontinuation of benzodiazepines for insomnia: a survival analysis of relapse. Behav Res Ther 2005;43(1):1-14 |
| 210 | **Belanger 2005** | Belanger L, Morin CM, Bastien C. Benzodiazepine discontinuation among adults with GAD: a randomized trial of cognitive-behavioural therapy. Behav Res Ther 2005;43(1):1-14 |
| 211 | **Baillargeon 2003** | Baillargeon L, Landreville P, Verreault R, Beauchemin JP, Gregoire JP, Morin CM. Discontinuation of benzodiazepines among older insomniac adults treated with cognitive-behavioural therapy combined with gradual tapering: a randomized trial. CMAJ 2003;169(10):1015-20 |
| 212 | **Zitman 2002** | Zitman FG, Couvee JE. Chronic benzodiazepine use in general practice: psychometric evaluation. Addiction 2002;97(3):337-42 |
| 213 | **Rickels 1999** | Rickels K, Schweizer E, Case WG, Greenblatt DJ. Tapering benzodiazepine use with paroxetine. Psychopharmacology (Berl) 1999;141(1):1-5 |
| 214 | **Udelman 1990** | Udelman HD, Udelman DL. Concurrent use of buspirone in anxious patients during withdrawal from alprazolam therapy. J Clin Psychiatry 1990;51(9):46-52 |
| 215 | **Voshaar 2006** | Voshaar RC, Gorgels WJ, Mol AJ, et al. Tapering off long-term benzodiazepine use with or without group cognitive therapy. Pharm World Sci 2006;29(6):641-6 |
| 216 | **Voshaar 2007** | Mol AJ, Oude Voshaar RC, Gorgels WJ, et al. Benzodiazepine craving in a general practice trial. J Clin Psychiatry 2007;68(12):1894-900 |
| 217 | **Nardi 2010** | Nardi AE, Freire RC, Valença AM, et al. Tapering clonazepam in patients with panic disorder after at least 3 years of treatment. J Clin Psychopharmacol 2010;30(3):290-3. |
| 218 | **Tyrer 1983** | Tyrer P, Owen R, Dawling S. Gradual withdrawal of diazepam after long-term therapy. Lancet 1983;1(8339):1402-6. |
| 219 | **Busto 1986** | Busto U, Sellers EM, Naranjo CA, et al. Withdrawal reaction after long-term therapeutic use of benzodiazepines. N Engl J Med 1986;315(14):854-9. |
| 220 | **Couvee 2002** | Couvee JE, Zitman FG. The benzodiazepine withdrawal symptom questionnaire: psychometric evaluation during a discontinuation program in depressed chronic benzodiazepine users in general practice. Addiction 2002;97(3):337-45. |
| 221 | **Schweizer 1989** | Schweizer E, Case WG, Rickels K. Benzodiazepine dependence and withdrawal in elderly patients. Am J Psychiatry 1989;146(4):529-31. |
| 222 | **Gosselin 2006** | Gosselin P, Ladouceur R, Morin CM, et al. Benzodiazepine discontinuation among adults with GAD: a randomized trial of cognitive-behavioral therapy. J Consult Clin Psychol 2006;74(5):908-19. |
| 223 | **Vicens 2006** | Vicens C, Fiol F, Llobera J, et al. Withdrawal from long-term benzodiazepine use: randomised trial in family practice. Br J Gen Pract 2006;56(533):958-63. |
| 224 | **Voshaar 2003** | Voshaar RC, Gorgels WJ, Mol AJ, et al. Tapering off long-term benzodiazepine use with or without group cognitive-behavioural therapy: three-conditioned, randomised controlled trial. Br J Psychiatry 2003;182:498-504. |
| 225 | **Schweizer 1998** | Schweizer E, Rickels K, De Martinis N, et al. The effect of personality on withdrawal severity and taper outcome in benzodiazepine dependent patients. Psychol Med 1998;28(3):713-20. |
| 226 | **Voshaar 2006** | Voshaar RC, Gorgels WJ, Mol AJ, et al. Predictors of long-term benzodiazepine abstinence in participants of a randomized controlled benzodiazepine withdrawal program. Can J Psychiatry 2006;51(7):445-52. |
| 227 | **Mol 2006** | Mol AJ, Oude Voshaar RC, Gorgels WJ, et al. The absence of benzodiazepine craving in a general practice benzodiazepine discontinuation trial. Addict Behav 2006;31(2):211-22. |
| 228 | **Mol 2007** | Mol AJ, Oude Voshaar RC, Gorgels WJ, et al. The role of craving in relapse after discontinuation of long-term benzodiazepine use. J Clin Psychiatry 2007;68(12):1894-900. |
| 229 | **Couvee 2002** | Couvee JE, Timmermans MA, Zitman FG; Dutch Chronic Benzodiazepine Working Group. The long-term outcome of a benzodiazepine discontinuation programme in depressed outpatients. J Affect Disord 2002;70:133-41. |
| 230 | **Couvee 2002** | Couvee JE, Bakker A, Zitman FG. The relevance of psychiatric and somatic comorbidity in depressed chronic benzodiazepine users. Psychother Psychosom 2002;71(5):263-8. |
| 231 | **Curran 2003** | Curran HV, Collins R, Fletcher S, et al. Older adults and withdrawal from benzodiazepine hypnotics in general practice: effects on cognitive function, sleep, mood and quality of life. Psychol Med 2003;33(7):1223-37. |
| 232 | **Schweizer 1995** | Schweizer E, Case WG, Garcia-Espana F, et al. Progesterone co-administration in patients discontinuing long-term benzodiazepine therapy: effects on withdrawal severity and taper outcome. Psychopharmacology (Berl) 1995;117(4):424-9. |
| 233 | **Vissers 2007** | Vissers FH, Knipschild PG, Crebolder HF. Is melatonin helpful in stopping the long-term use of hypnotics? A discontinuation trial. Pharm World Sci 2007;29(6):641-6. |
| 234 | **Chen et al., 2010** | Chen DR, Sethi KB, Mucklow JC. Discontinuing Benzodiazepine Therapy: An Interdisciplinary Approach at a Geriatric Day Hospital. Can. Pharm. J. 2010, 143, 286–295.e3. |
| 235 | **Canham et al., 2014** | Canham, S.L.; Gallo, J.; Simoni-Wastila, L. Perceptions of Benzodiazepine Dependence Among Women Age 65 and Older. J. Gerontol. Soc. Work. 2014, 57, 872–888. |
| 236 | **Heser et al., 2018** | Heser, K.; Pohontsch, N.J.; Scherer, M.; Löffler, A.; Luck, T.; Riedel-Heller, S.G.; Maier, W.; Parker, D.; Haenisch, B.; Jessen, F. Perspective of elderly patients on chronic use of potentially inappropriate medication–Results of the qualitative CIM-TRIAD study. PLoS ONE 2018, 13, e0202068. |
| 237 | **Martin y Tannenbaum, 2017** | Martin, P.; Tannenbaum, C. A realist evaluation of patients’ decisions to deprescribe in the EMPOWER trial. BMJ Open 2017, 7, e015959. |
| 238 | **Williams et al., 2016** | Williams, F.; Mahfouz, C.; Bonney, A.; Pearson, R.; Seidel, B.; Dijkmans-Hadley, B.; Ivers, R. A circle of silence: The attitudes of patients older than 65 years of age to ceasing long-term sleeping tablets. Aust. Fam. Physician 2016, 45, 506–511. |
| 239 | **Cook et al., 2007** | Cook, J.M.; Marshall, R.; Masci, C.; Coyne, J.C. Physicians’ Perspectives on Prescribing Benzodiazepines for Older Adults: A Qualitative Study. J. Gen. Intern. Med. 2007, 22, 303–307. |
| 240 | **Šubelj et al., 2010** | Šubelj, M.; Vidmar, G.; Švab, V. Prescription of benzodiazepines in Slovenian family medicine: A qualitative study. Wien. Klin. Wochenschr. 2010, 122, 474–478. |
| 241 | **Anthierens et al., 2009** | Anthierens, S.; Grypdonck, M.; De Pauw, L.; Christiaens, T. Perceptions of nurses in nursing homes on the usage of benzodiazepines. J. Clin. Nurs. 2009, 18, 3098–3106. |
| 242 | **Pickering et al., 2020** | Pickering, A.N.; Hamm, M.E.; Bs, A.D.; Hanlon, J.T.; Thorpe, C.T.; Gellad, W.F.; Radomski, T.R. Older Patient and Caregiver Perspectives on Medication Value and Deprescribing: A Qualitative Study. J. Am. Geriatr. Soc. 2020, 68, 746–753. |
| 243 | **Cormack 1989** | Cormack MA, Owens RG, Dewey ME: The effect of minimal interventions by general practitioners on long-term benzodiazepine use. J R Coll Gen Pract. 1989, 39 (327): 408-411. |
| 244 | **Gorgels 2005** | Gorgels WJMJ, Oude Voshaar RC, Mol AJJ, Van De Lisdonk EH, Van Balkom AJLM, Van Den Hoogen HJM, Mulder J, Breteler MHM, Zitman FG: Discontinuation of long-term benzodiazepine use by sending a letter to users in family practice: A prospective controlled intervention study. Drug and Alcohol Dependence. 2005, 78 (1): 49-56. 10.1016/j.drugalcdep.2004.09.001. |
| 245 | **Morrison 1990** | Morrison JM: Audit and follow-up of chronic benzodiazepine tranquillizer use in one general practice. Family Practice. 1990, 7 (4): 253-257. 10.1093/fampra/7.4.253. |
| 246 | **Zwar 2000** | Zwar NA, Wolk J, Gordon JJ, Sanson-Fisher RW: Benzodiazepine prescribing by GP registrars. A trial of educational outreach. Aust Fam Physician. 2000, 29 (11): 1104-1107. |
| 247 | **Cormack 1999** | Cormack MA, Sweeney KG, Hughes-Jones H, Foot GA. Evaluation of an easy, cost-effective strategy for cutting benzodiazepine use in general practice. Br J Gen Pract. 1994;44(378):5-8. |
| 248 | **Midlov 2006** | Midlov P, Bondesson A, Eriksson T, Nerbrand C, Hoglund P: Effects of educational outreach visits on prescribing of benzodiazepines and antipsychotic drugs to elderly patients in primary health care in southern Sweden. Family Practice. 2006, 23 (1): 60-64. 10.1093/fampra/cmi105. |
| 249 | **Smith 1998** | Smith DH, Christensen DB, Stergachis A, Holmes G: A randomized controlled trial of a drug use review intervention for sedative hypnotic medications. Med Care. 1998, 36 (7): 1013-1021. 10.1097/00005650-199807000-00008. |
| 250 | **Dollman 2005** | Dollman WB, LeBlanc VT, Stevens L, O'Connor PJ, Roughead EE, Gilbert AL. Achieving a sustained reduction in benzodiazepine use through implementation of an area-wide multi-strategic approach. J Clin Pharm Ther. 2005;30(5):425-432. https://doi.org/10.1111/j.1365-2710.2005.00674.x. |
| 251 | **Towle 2006** | Towle I, Adams J. A novel, pharmacist-led strategy to reduce the prescribing of benzodiazepines in Paisley. Pharm J. 2006;276:136-138. |
| 252 | **Brymer 2000** | Brymer C, Rusnell I. Reducing substance dependence in elderly people: The side effects program. J Popul Ther Clin Pharmacol. 2000;7(3):161-166. |
| 253 | **Onyett 1988** | Onyett SR, Turpin G. Benzodiazepine withdrawal in primary care: A comparison of behavioural group training and individual sessions. Behav Psychother. 1988;16(4):297-312. https://doi.org/10.1017/S0141347300014154. |
| 254 | **de Burgh 1995** | de Burgh S, Mant A, Mattick RP, Donnelly N, Hall W, Bridges-Webb C. A controlled trial of educational visiting to improve benzodiazepine prescribing in general practice. Aust J Public Health. 1995;19(2):142-148. https://doi.org/10.1111/j.1753-6405.1995.tb00364.x. |
| 255 | **Smith 1998** | Smith DH, Christensen DB, Stergachis A, Holmes G. A randomized controlled trial of a drug use review intervention for sedative hypnotic medications. Med Care. 1998;36(7):1013-1021. https://doi.org/10.1097/00005650-199807000-00008. |
| 256 | **Berings 1994** | Berings D, Blondeel L, Habraken H. The effect of industry-independent drug information on the prescribing of benzodiazepines in general practice. Eur J Clin Pharmacol. 1994;46(6):501-505. https://doi.org/10.1007/BF00196105. |
| 257 | **Holm 1990** | Holm M. Intervention against long-term use of hypnotics/sedatives in general practice. Scand J Prim Health Care. 1990;8(2):113-117. https://doi.org/10.3109/02813439008994941. |
| 258 | **Eide 2001** | Eide E, Schjott J. Assessing the effects of an intervention by a pharmacist on prescribing and administration of hypnotics in nursing homes. Pharm World Sci. 2001;23(6):227-231. https://doi.org/10.1023/A:1015142211348. |
| 259 | **Batty 2001** | Batty GM, Oborne CA, Hooper R, Jackson SHD. Investigating intervention strategies to increase the appropriate use of benzodiazepines in elderly medical in-patients. Br J Clin Govern. 2001;6(4):252-258. https://doi.org/10.1108/14664100110408608. |
| 260 | **Gilbert 1993** | Gilbert A, Owen N, Innes JM, Sansom L. Trial of an intervention to reduce chronic benzodiazepine use among residents of aged-care accommodation. Aust N Z J Med. 1993;23(4):343-347. |
| 261 | **Elliott 2001** | Elliott RA, Woodward MC, Oborne CA. Improving benzodiazepine prescribing for elderly hospital inpatients using audit and multidisciplinary feedback. Intern Med J. 2001;31(9):529-535. https://doi.org/10.1046/j.1445-5994.2001.00139.x. |
| 262 | **Holden 1994** | Holden JD, Hughes IM, Tree A. Benzodiazepine prescribing and withdrawal for 3234 patients in 15 general practices. Fam Pract. 1994;11(4):358-362. https://doi.org/10.1093/fampra/11.4.358. |
| 263 | **Pimlott 2003** | Pimlott NJG, Hux JE, Wilson LM, Kahan M, Li C, Rosser WW. Educating physicians to reduce benzodiazepine use by elderly patients: A randomized controlled trial. CMAJ. 2003;168(7):835-839. |
| 264 | **Schmidt 1998** | Schmidt I, Claesson CB, Westerholm B, Nilsson LG, Svarstad BL: The impact of regular multidisciplinary team interventions on psychotropic prescribing in Swedish nursing homes. Journal of the American Geriatrics Society. 1998, 46 (1): 77-82. |
| 265 | **Cantopher 1990** | Cantopher T, Olivieri S, Cleave N, Edwards JG. Chronic benzodiazepine dependence. A comparative study of abrupt withdrawal under propranolol cover versus gradual withdrawal. Br J Psychiatry. 1990;156:406–411. |
| 266 | **Petrovic 1999** | Petrovic M, Pevernagie D, Van Den Noortgate N, Mariman A, Michielsen W, Afschrift M. A programme for short-term withdrawal from benzodiazepines in geriatric hospital inpatients: Success rate and effect on subjective sleep quality. Int J Geriatr Psychiatry. 1999;14:754–760. |
| 267 | **Delle Chiaie 1995** | Delle Chiaie R, Pancheri P, Casacchia M, Stratta P, Kotzalidis GD, Zibellini M. Assessment of the efficacy of buspirone in patients affected by generalized anxiety disorder, shifting to buspirone from prior treatment with lorazepam: a placebo-controlled, double-blind study. J Clin Psychopharmacol. 1995;15:12–19. |
| 268 | **Rickels 2000** | Rickels K, DeMartinis N, García-España F, Greenblatt DJ, Mandos LA, Rynn M. Imipramine and buspirone in treatment of patients with generalized anxiety disorder who are discontinuing long-term benzodiazepine therapy. Am J Psychiatry. 2000;157:1973–1979. |
| 269 | **Rynn 2003** | Rynn M, García-España F, Greenblatt DJ, Mandos LA, Schweizer E, Rickels K. Imipramine and buspirone in patients with panic disorder who are discontinuing long-term benzodiazepine therapy. J Clin Psychopharmacol. 2003;23:505–508. |
| 270 | **Croissant 2008** | Croissant B, Grosshans M, Diehl A, Mann K. Oxcarbazepine in rapid benzodiazepine detoxification. Am J Drug Alcohol Abuse. 2008;34:534–540. |
| 271 | **Ries 1989** | Ries RK, Roy-Byrne PP, Ward NG, Neppe V, Cullison S. Carbamazepine treatment for benzodiazepine withdrawal. Am J Psychiatry. 1989;146:536–537. |
| 272 | **Garcia-Borreguero 1991** | Garcia-Borreguero D, Bronisch T, Apelt S, Yassouridis A, Emrich HM. Treatment of benzodiazepine withdrawal symptoms with carbamazepine. Eur Arch Psychiatry Clin Neurosci. 1991;241:145–150. |
| 273 | **Bobes 2012** | Bobes J, Rubio G, Terán A, et al. Pregabalin for the discontinuation of long-term benzodiazepines use: an assessment of its effectiveness in daily clinical practice. Eur Psychiatry. 2012;27:301–307. |
| 274 | **Tyrer 1981** | Tyrer P, Rutherford D, Huggett T. Benzodiazepine withdrawal symptoms and propranolol. Lancet. 1981;1:520–522. |

Appendix 4 – Search strategy in line-by-line format

| Question component | # | Boolean strategy |
| --- | --- | --- |
| Population | #1 | (Disinvest* OR “decrease use” OR discontinu* OR abandon* OR reassess* OR obsole* OR “medical reversal” OR contradict OR re-invest OR withdraw* OR reduc* OR “decline in use” OR “health technology reassessment” OR “change in use” OR de-implement* OR de-list OR “Low value practice/intervention” OR “change in practice” OR de-adopt* OR de-commission OR “do not do” OR reallocation OR remov* OR replace OR refute OR over use OR stop* OR “inappropriate use” OR relinquish* OR ineffective OR misuse OR re-appraisal OR re-prioritization OR “substitutional re-investment” OR “evidence-based reassessment” OR “clinical redesign” OR disadoption OR defunding OR “resource release” OR “withdrawing from a service and redeploying resources” OR redeploy OR reversal OR “drop in use”) |
|  | #2 | (benzo* OR Benzodiazepines OR Alprazolam OR Benzodiazepinones OR Anthramycin OR Bromazepam OR Clonazepam OR Devazepide OR Diazepam OR Flumazenil OR Flunitrazepam OR Flurazepam OR Lorazepam OR Nitrazepam OR Oxazepam OR Pirenzepine OR Prazepam OR Temazepam OR Chlordiazepoxide OR Clobazam OR “Clorazepate Dipotassium” OR Estazolam OR Medazepam OR Midazolam OR Olanzapine OR Triazolam) |
|  | #3 | #1 AND #2 AND |
| Intervention | #4 | (Deprescription OR Deprescribing OR "Inappropriate Prescribing" OR reduce* OR reducing OR reduction OR “low-value care”)* |
|  | #5 | #4 AND #5 |
| Study design | #6 | “Systematic review” |
|  | #7 | "critical review" OR "electronic search" OR "evidence-based analysis" OR "evidence-based review" OR "literature search" OR "meta analysis" OR "meta synthesis" OR "meta-analyse" OR "meta-analytic review" OR "meta-study" OR "meta-synthesis" OR "metaanalysis" OR "metasynthesis" OR "meta-analysis" OR "pooled effect" OR "random-effects model" OR "systematic quantitative review" OR "systematically searched" OR "systemic review" OR (review AND randomized) OR (systematic AND review) OR MEDLINE OR "literature review" OR PubMed |
|  | #8 | #6 OR #7 |
| Combination of terms | #9 | #3 AND #5 AND #8 |

Appendix 5 – Strategy in block format

(Disinvest* OR “decrease use” OR discontinu* OR abandon* OR reassess* OR obsole* OR “medical reversal” OR contradict OR re-invest OR withdraw* OR reduc* OR “decline in use” OR “health technology reassessment” OR “change in use” OR de-implement* OR de-list OR “Low value practice/intervention” OR “change in practice” OR de-adopt* OR de-commission OR “do not do” OR reallocation OR remov* OR replace OR refute OR over use OR stop* OR “inappropriate use” OR relinquish* OR ineffective OR misuse OR re-appraisal OR re-prioritization OR “substitutional re-investment” OR “evidence-based reassessment” OR “clinical redesign” OR disadoption OR defunding OR “resource release” OR “withdrawing from a service and redeploying resources” OR redeploy OR reversal OR “drop in use”) AND (Deprescription OR Deprescribing OR "Inappropriate Prescribing" OR reduce* OR reducing OR reduction OR “low-value care”) AND (benzo* OR Benzodiazepines OR Alprazolam OR Benzodiazepinones OR Anthramycin OR Bromazepam OR Clonazepam OR Devazepide OR Diazepam OR Flumazenil OR Flunitrazepam OR Flurazepam OR Lorazepam OR Nitrazepam OR Oxazepam OR Pirenzepine OR Prazepam OR Temazepam OR Chlordiazepoxide OR Clobazam OR “Clorazepate Dipotassium” OR Estazolam OR Medazepam OR Midazolam OR Olanzapine OR Triazolam) AND ("critical review" OR "electronic search" OR "evidence-based analysis" OR "evidence-based review" OR "literature search" OR "meta analysis" OR "meta synthesis" OR "meta-analyse" OR "meta-analytic review" OR "meta-study" OR "meta-synthesis" OR "metaanalysis" OR "metasynthesis" OR "meta-analysis" OR "pooled effect" OR "random-effects model" OR "systematic quantitative review" OR "systematically searched" OR "systemic review" OR (review AND randomized) OR (systematic AND review) OR MEDLINE OR "literature review" OR PubMed)

**Strategy for Pubmed/MEDLINE**

#644

**Strategy for Cochrane Library**

#172

**Strategy for PsycInfo**

#3283

**Strategy for Epistemonikos** #968

# Appendix 6 – List of excluded studies

| **Nro** | **Author and year** | **Title** | **Reason** | **Justified Reason*** | **Comments** |
| --- | --- | --- | --- | --- | --- |
| 1 | Ashton 2009 | Review: brief interventions, gradual dose reduction and psychological interventions increase benzodiazepine cessation compared with routine care | Wrong Design | This is a summary of the article by Parr et al. (2009) | It is a summary of the article included |
| 2 | Capiau A et al., 2022 | Therapeutic dilemmas with benzodiazepines and Z-drugs: insomnia and anxiety disorders versus increased fall risk: a clinical review. | Wrong Intervention | This study does not align with the objective, as it focuses more on the treatment of abuse and dependence. | Focuses on adverse effects and safety rather than on reducing prescriptions. |
| 3 | Darker et al., 2015 | Psychosocial interventions for benzodiazepine harmful use, abuse or dependence | Wrong Intervention | This article focuses more on adverse effects and recommendations for appropriate use in older adults, rather than on reducing prescriptions. | Does not address strategies for reducing prescriptions in primary care. |
| 4 | Durán-Rivera et al., 2022 | Deprescribing medications for elderly patients: a narrative review | Wrong Design | Narrative Review | Not a systematic review. |
| 5 | Evrard P et al., 2022 | Barriers and enablers for deprescribing benzodiazepine receptor agonists in older adults: a systematic review of qualitative and quantitative studies using the theoretical domains framework. | Wrong Intervention | The objective is the analysis of barriers and facilitators for deprescription. | Does not directly evaluate strategies for reducing prescriptions in primary care. |
| 6 | Fenn NE and Plake KS, 2017 | Opioid and Benzodiazepine Weaning in Pediatric Patients: Review of Current Literature | Wrong Population | Pediatric patients | Does not apply to adults or primary care. |
| 7 | Guaiana G , Barbui C, 2016 | Discontinuing benzodiazepines: best practices | Wrong Design | Rapid literature summary | Not a systematic review. |
| 8 | Mokhar et al., 2018 | Patient-centered care interventions to reduce the inappropriate prescription and use of benzodiazepines and z-drugs: a systematic review | Wrong Population | Focuses on patients rather than primary care professionals. | Does not evaluate strategies directed at primary care physicians. |
| 9 | Ostini et al., 2011 | How is medication prescribing ceased? A systematic review. | Wrong Intervention | Does not focus exclusively on reducing benzodiazepines. Not restricted to studies in primary care. Includes a variety of medications and healthcare settings. | Excluded for including multiple medications and not focusing on primary care. |
| 10 | Pollmann et al., 2015 | Deprescribing benzodiazepines and Z-drugs in community-dwelling adults: a scoping review | Wrong Population | Not primary care. "We limited our target population to patients taking benzodiazepines and Z-drugs in the community or outpatient settings, as individuals receiving care in inpatient, long-term care, or residential aged care facilities can differ systematically with respect to numerous factors." | Does not focus on physicians or primary care teams. |
| 11 | Reeve et al., 2017 | A systematic review of interventions to deprescribe benzodiazepines and other hypnotics among older people | Wrong Population | Not primary care. "A systematic review of interventions to deprescribe benzodiazepines and other hypnotics among older people" | Focuses on the general older population without specifying primary care physicians. |
| 12 | Ribeiro and Schlindwein 2021 | Benzodiazepine deprescription strategies in chronic users: a systematic review. | Wrong Population | Focuses on chronic patients without specifying strategies directed at primary care physicians. | Does not meet the criteria for including interventions specific to primary care. |
| 13 | Sirdifield C et al., 2013 | General practitioners' experiences and perceptions of benzodiazepine prescribing: systematic review and meta-synthesis | Wrong Design | Does not evaluate strategies for reducing prescriptions, but rather perceptions. | Excluded for not focusing on specific deprescribing strategies in primary care. |
| 14 | Sirdifield C et al., 2017 | A Systematic Review and Meta-Synthesis of Patients' Experiences and Perceptions of Seeking and Using Benzodiazepines and Z-Drugs: Towards Safer Prescribing | Wrong Design | Not a review of strategies, but of patient perceptions. | Does not meet the inclusion criteria for evaluating prescription reduction strategies in primary care. |
| 15 | Swedish Council on Health Technology Assessment | How Can Drug Consumption among the Elderly be Improved?: A Systematic Review [Internet] | Wrong Intervention | Does not focus on specific strategies for reducing benzodiazepine prescriptions in primary care. | Does not meet the inclusion criteria. |
| 16 | Wang et al., 2023 | Deprescribing Strategies for Opioids and Benzodiazepines with Emphasis on Concurrent Use: A Scoping Review | Wrong Design | Scoping review, not a systematic review | Excluded due to study design. |

# Appendix 7– Data extraction form

| **Variable** | **Description** |
| --- | --- |
| **ID** | Unique identifier for each included systematic review. |
| **Author(s) and Year** | Names of the lead author(s) and the publication year of the systematic review. |
| **Title** | Full title of the systematic review. |
| **Country of Origin** | The country where the systematic review was conducted. |
| **Funding Source (if reported)** | Source of funding for the study (e.g., government, private sector, nonprofit). |
| **Last Search Date** | The date of the last bibliographic search reported in the systematic review. |
| **Year of Most Recent Study** | The year of the most recent study included in the systematic review. |
| **Number of Included Studies** | The total number of studies included in the systematic review. |
| **Number of RCTs** | The number of randomized controlled trials (RCTs) included in the systematic review. |
| **Study Designs Included** | Types of study designs included in the review (e.g., RCTs, observational studies, qualitative studies). |
| **Population** |  |
| **Age Group (Young Adults, Adults, Older Adults)** | Age range of participants in the included studies (e.g., 18-49 years, 50+ years). |
| **Sample Size** | The total number of participants analyzed across the included studies. |
| **Country/Region of Studies** | Countries or regions where the included studies were conducted. |
| **Health Professionals Involved** | Types of healthcare professionals involved in the intervention (e.g., general practitioners, pharmacists). |
| **Family Support Mentioned? (Yes/No)** | Whether the study considered the role of family members in supporting deprescribing. |
| **Intervention** |  |
| **Intervention Strategy** | Description of the deprescribing intervention(s) assessed (e.g., gradual dose reduction, cognitive behavioral therapy). |
| **Comparator (if applicable)** | If applicable, the comparator group used in the studies (e.g., usual care, alternative intervention). |
| **Theoretical Framework Used? (Yes/No)** | Whether the study reported using a theoretical model to guide the intervention. |
| **Adherence to Intervention Reported? (Yes/No)** | Whether the study measured and reported adherence to the intervention. |
| **Barriers and Facilitators** |  |
| **Barriers to De-adoption** | Identified challenges in implementing benzodiazepine deprescribing interventions (e.g., patient reluctance, withdrawal symptoms). |
| **Facilitators to De-adoption** | Identified factors that support successful deprescribing interventions (e.g., shared decision-making, education programs). |
| **Outcomes Assessed** |  |
| **Medication Outcomes** | Changes in benzodiazepine prescribing or consumption rates (e.g., complete cessation, dose reduction). |
| **Clinical Outcomes** | Health effects observed from the intervention (e.g., improvements in anxiety, sleep quality, withdrawal symptoms). |
| **System Outcomes** | System-level changes, such as prescription trends in healthcare facilities. |
| **Implementation Outcomes** | Metrics related to the implementation process (e.g., feasibility, acceptability, uptake by clinicians). |
| **Unexpected Outcomes** | Any unintended consequences or effects reported in the study. |
| **Effectiveness of the Intervention** |  |
| **Effectiveness Findings** | Summary of the effectiveness results reported in the systematic review. |
| **Reported Follow-up Duration** | Duration of follow-up periods in the included studies (e.g., 6 months, 12 months). |
| **Statistical Significance (if reported)** | Whether the intervention effect was statistically significant (e.g., p-value, confidence intervals). |
| **Risk of Bias and Quality Assessment** |  |
| **Risk of Bias Tool Used** | The tool used to assess the risk of bias in the included studies (e.g., Cochrane Risk of Bias, CONSORT). |
| **What is the risk? (description)** | A summary of the risk of bias findings reported in the systematic review. |
| **Meta-Analysis Conducted? (Yes/No)** | Whether the systematic review included a meta-analysis. |
| **Certainty of Evidence (GRADE)** | The certainty of the evidence based on the GRADE (Grading of Recommendations, Assessment, Development, and Evaluation) system. |
| **Limitations Reported by Authors** | Limitations mentioned by the authors of the systematic review (e.g., small sample sizes, lack of long-term follow-up). |

# Appendix 8 – Risk of Bias Assessment (Critical Appraisal of Systematic Reviews)

This is an example of using an Excel spreadsheet (version 10; Microsoft Inc.) for Risk of Bias Assessment (Critical Appraisal of Systematic Reviews) based on AMSTAR 2.

Reference:

Shea BJ, Reeves BC, Wells G, Thuku M, Hamel C, Moran J, Moher D, Tugwell P, Welch V, Kristjansson E, Henry DA. AMSTAR 2: a critical appraisal tool for systematic reviews that include randomised or non-randomised studies of healthcare interventions, or both. BMJ. 2017 Sep 21;358

|  | **A M S T A R 2** |  |  |  |  |
| --- | --- | --- | --- | --- | --- |
|  |  |  | **Author Year** | **Ashkanani 2023** | |
| **Criteria** | **Question** | **Description** | **Description** | **Vote** | **Quotes** |
| 1. | Did the research questions and inclusion criteria for the review include the components of PICO? | **For yes:   - Population  - Intervention  - Comparator group  - Outcome** | Optionnal (recommended)  - Timeframe for follow-up | YES |  |
| 2. | Did the report of the review contain an explicit statement that the review methods were established prior to the conduct of the review and did the report justify any significant deviations from the protocol? | **For Partial Yes: The authors state that they had written protocol or guide that included ALL the following:   - review question(s)  - a search strategy  - inclusion/exclusion criteria  - a risk of bias assessment** | **For Yes: As for partial yes, plus the protocol should be registered and should also have specfied:  - a meta-analysis/synthesis plan, if appropiate, and - a plan for investigating causes of heterogenity - justification for any deviations from the protocol** | YES |  |
| 3. | Did the review authors explain their selection of the study designs for inclusion in the review? | **For Yes, the review should satisfy ONE of the following:  - explanation for including only RCTs - OR explanation for including only NRSI - OR explanation for including both RCTs and NRSI** |  | YES |  |
| 4. | Did the review authors use a comprehensive literature search strategy? | **For Partial Yes (all the following):  - searched at least 2 databases (relevant to research question) - provided key word and/or search strategy - justified publication restrictions (e.g. language)** | **For Yes, also have (all the flowing):  - searched the reference list/bibliographies of included studies - searched trial/study registries - included/consulted content experts in the field - where relevant, searched for grey literature** | YES |  |
| 5. | Did the review authors perform study selection in duplicate? | **For Yes, either ONE of the following:  - at least two reviewers independently agreed on selection of elegible studies and achieved consensus on which studies to include - OR two reviewers selected a sample of eligible studies and achieved good agreement (at least 80 percent), with the remainder selected by one reviewer.** |  | YES |  |
| 6. | Did the review authors perform data extraction in duplicate? | **For Yes, either ONE of the following:  - at least two reviewers achieved consensus on which data to extract from included studies - OR two reviewers extracted data from a sample of eligible studies and achieved good agreement (at least 80 percent), with the remainder extracted by one reviewer** |  | YES |  |
| 7. | Did the review authors provide a list of excluded studies and justify the exclusions? | **For Partial Yes:  - provided a list of all potentially relevant studies that were read in full-text form but excluded from the review** | **For Yes, must also have:  - justified the exclusion from the review of each potentially relevant study** | YES |  |
| 8. | Did the review authors describe the included studies in adequate detail? | **For Partial Yes:  - described populations - described interventions - described comparators - described outcomes - described research designs** | **For Yes, must also have ALL the following:  - described population in detail - described intervention in detail (including doses where relevant) - described comparator in detail (including doses where relevant) - described study's setting - timeframe for follow-up** | YES |  |
| 9. | Did the review authors use a satisfactory technique for assessing the risk of bias (RoB) in individual studies that were included in the review? | **RCTs For Partial Yes, must have assessed RoB from:  - unconcealed allocation, and - lack of blinding of patients and assessors when assessing outcomes (unnecessary for objective outcomes such as all- cause mortality)** | **RCTs For Yes, must also have assessed RoB from:  - allocation sequence that was not truly random, and - selection of the reported result from among multiple measurements or analyses of a specified outcome** | YES |  |
|  |  | **NRSI For Partial Yes, must have assessed RoB:  - from confounding, and - from selection bias** | **NRSI For Yes, must also have assessed RoB:  - methods used to ascertain exposures and outcomes, and  - selection of the reported result from among multiple measurements or analyses of a specified outcome** |  |  |
| 10. | Did the review authors report on the sources of funding for the studies included in the review? | **For Yes:  - Must have reported on the resources of funding of individual studies included in the review. Note: Reporting that the reviewers looked for this information but it was not reported by study authors also qualifies** |  | NO |  |
| 11. | If meta-analysis was performed did the review authors use appropriate methods for statistical combination of results? | **RCTs For Yes:  - The authors justified combining the data in a meta-analysis - AND they used an appropriate weighted technique to combine study results and adjusted for heterogeneity** |  | YES |  |
|  |  | **NRSI For Yes:  - The authors justified combining the data in a meta-analysis - AND they used an appropriate weighted technique to combine study results, adjusting for heterogeneity if present - AND they statistically combined effect estimates from NRSI that were adjusted for confounding, rather than combining raw data, or justified combining raw data when adjusted effect estimates were not available - AND they reported separate summary estimates for RCTs and NRSI separately when both were included in the review** |  |  |  |
| 12. | If meta-analysis was performed, did the review authors assess the potential impact of RoB in individual studies on the results of the meta-analysis or other evidence synthesis? | **For Yes:  - Included only low risk of bias RCTs - OR, if the pooled estimate was based on RCTs and/or NRSI at variable RoB, the authors performed analyses to investigate possible impact of RoB on summary estimates of effect** |  | YES |  |
| 13. | Did the review authors account for RoB in individual studies when interpreting/ discussing the results of the review? | **For Yes:  - included only low risk of bias RCTs - OR, if RCTs with moderate or high RoB, or NRSI were included the review provided a discussion of the likely impact of RoB on the results** |  | YES |  |
| 14. | Did the review authors provide a satisfactory explanation for, and discussion of, any heterogeneity observed in the results of the review? | **For Yes:  - There was no significant heterogeneity in the results - OR if heterogeneity was present the authors performed an investigation of sources of any heterogeneity in the results and discussed the impact of this on the results of the review** |  | YES |  |
| 15. | If they performed quantitative synthesis did the review authors carry out an adequate investigation of publication bias (small study bias) and discuss its likely impact on the results of the review? | **For Yes:  - Performed graphical or statistical tests for publication bias and discussed the likelihood and magnitude of impact of publication bias** |  | YES |  |
| 16. | Did the review authors report any potential sources of conflict of interest, including any funding they received for conducting the review? | **For Yes:  - The authors reported no competing interests OR - The authors described their funding sources and how they managed potential conflicts of interest** |  | YES |  |
|  |  |  | **Overall Confidence** | **High** |  |

# Appendix 9 – Characteristics of Systematic Reviews

Characteristics SR

| **Appendix 13 – Characteristics of Systematic Reviews** | | | | | | | | | | | |
| --- | --- | --- | --- | --- | --- | --- | --- | --- | --- | --- | --- |
| **Author and year** | **Title** | **Aim of the SR** | **Number of studies included** | **Participants (total number)** | **Year of the most recent study** | **search period or date** | **Place (country -geographic location),** | **Only RCT** | **Only non-RCT** | **RCT and non RCT** | **Number_RCT** |
| Bailey 2021 | Interactive digital interventions for prevention of sexually transmitted HIV. | assesses the effectiveness of interactive digital interventions (IDIs) for prevention of sexually transmitted HIV. | 31 | 11293 | 2017 | from 2014 to June 2017 | USA (27/31),Netherlands (1/31), Uganda (1/31), Zambia (1/31) and Sweden (1/31) | Yes | No | No | 31 |
| Berendes 2021 | Sexual health interventions delivered to participants by mobile technology: a systematic review and meta-analysis of randomised controlled trials. | To assess the effectiveness of mobile health interventions delivered to participants for preventing STIs and promoting preventive behaviour. | 22 | 19 551 | 2020 | 1 January 2010–19 February 2020 | 12 trials had been conducted in HICs (USA: n=6; Europe: n=3; Australia: n=3) and 10 in LMICs (Africa: n=7; China: n=2; India: n=1); | Yes | No | No | 22 |
| Burns 2016 | A systematic review of randomised control trials of sexual health interventions delivered by mobile technologies. | The purpose of this systematic review is to update our knowledge of and assess all mHealth interventions for clinic attendance for sexual health and safer sex behaviours (including STI testing, partner notification, condom use number of partners) for all populations, interventions, comparisons, outcomes and studies globally. | 10 | 16773 | 2014 | January 2010 and July 2014 | Irlanda 1/10, Sudafrica 1/10, australia 3/10, USA 3/10, Kanya 2/10 | Yes | No | No | 10 |
| Clarke 2022 | Increasing attendance at pre-booked sexual health consultations: a systematic review. | to identify the range and effectiveness of interventions implemented to improve attendance at pre-booked sexual health consultations | 13 | Not report | 2021 | 1 January 2000 to 1 September 2021. | 5 Australia, 5 America and 3 the United Kingdom. | No | No | Yes | 5 |
| Conserve 2017 | Systematic review of mobile health behavioural interventions to improve uptake of HIV testing for vulnerable and key populations. | This systematic narrative review examined the empirical evidence on the effectiveness of mobile health (mHealth) behavioral interventions designed to increase uptake of HIV testing among vulnerable and key populations. | 7 | Not report | 2015 | January 1, 2005 and August 1, 2015 | India, Australia, UK, Sudafrica, Kenia, USA y China | No | No | Yes | 2 |
| Ilskens 2022 | An Evidence Map on Serious Games in Preventing Sexually Transmitted Infections Among Adolescents: Systematic Review About Outcome Categories Investigated in Primary Studies. | The aim of this systematic review was to identify and systematically summarize the dimensions that have been investigated in primary studies on serious games targeting STI prevention among adolescents. | 18 | Nor report (from incluided table =15286) | 2021 | from 2009 to 2021 | India, Perú, Hong Kong, Puerto Rico, 10 USA, Tanzania, Brazil, Kenya, | No | No | Yes | 8 |
| Jones 2014 | The impact of health education transmitted via social media or text messaging on adolescent and young adult risky sexual behavior: a systematic review of the literature. | to examine the effectiveness of social media and text messaging interventions designed to increase sexually transmitted disease (STD) knowledge, increase screening/testing, decrease risky sexual behaviors, and reduce the incidence of STDs among young adults aged 15 through 24 years. | 11 | not report | 2014 | no especifica | no especifica | No | No | Yes | 5 |
| Kamitani 2024 | A Community Guide Systematic Review: Digital HIV Pre-exposure Prophylaxis Interventions. | to present the characteristics and effectiveness of digital PrEP adherence interventions. | 9 | not report | 2022 | from 2000 to 2022 | 8/9 USA, 1 netherlands | No | No | Yes | 5 |
| Khuwaja 2022 | Increasing HPV Vaccination Rates Using Text Reminders: An Integrative Review of the Literature. | The purpose of this review is to consider text message reminder system efficacy to improve HPV vaccination rates in eligible children. | 7 | not report | 2021 | between 2011 and 2021 | 6 USA y 1 Australia. | No | No | Yes | 4 |
| Knight 2017 | Online interventions to address HIV and other sexually transmitted and blood-borne infections among young gay, bisexual and other men who have sex with men: a systematic review. | To assess the status of published research (e.g. effectiveness; acceptability; differential effects across subgroups) involving online interventions that address HIV/STBBIs among young gbMSM. | 17 | 4669 | 2016 | from inception to November 2016 | 12 USA, Hong Kong, 2 Peru, China, and Thailand | No | No | Yes | 12 |
| Manby 2022 | Effectiveness of eHealth Interventions for HIV Prevention and Management in Sub-Saharan Africa: Systematic Review and Meta-analyses. | To systematically evaluate the effectiveness of eHealth interventions for sexually transmitted HIV prevention in SSA. (Sub‑Saharan Africa) | 25 | 15343 | 2020 | from 2000 to 2020 | all Sudafrica | Yes | No | No | 25 |
| Nguyen 2019 | A Systematic Review of eHealth Interventions Addressing HIV/STI Prevention Among Men Who Have Sex With Men. | The aim of this review was to summarize and appraise the existing eHealth interventions related to HIV/STI prevention among MSM. In addition, this study identified and summarized considerations of eHealth intervention implementation in order to sustain effectiveness over time among MSM populations. | 55 | not report | 2019 | through 1 June 2019 | United States (n = 36 studies) and Asia and Australia regions (n = 13 studies), Canada and Latin America (n=3) Europa (n=3) | No | No | Yes | 37 |
| Ou 2023 | The Effectiveness of mHealth Interventions Targeting Parents and Youth in Human Papillomavirus Vaccination: Systematic Review. | to conduct a systematic review to assess the effectiveness of mHealth interventions on parental intent to vaccinate youth against HPV and youth’s vaccine uptake. | 17 | not report | 2022 | January 2011 and December 2022 | 14 Usa, Netherlands, Australia, and Japan, | No | No | Yes | 12 |
| Palmer 2020 | Targeted client communication via mobile devices for improving sexual and reproductive health. | We assessed the effect of sending targeted messages by mobile devices to young people and adults about their sexual and reproductive health (SRH). Sexually transmitted infections (STIs) and unintended pregnancies are important causes of illness and early death worldwide. | 40 | 26854 | 2019 | enero de 2010 a julio de 2017), se exctulizó en 2019 | All the trials conducted among adolescent populations were carried out in high‐income countries, with the exception of one conducted in Ghana, a lower‐middle‐income country | Yes | No | No | 40 |
| Saragih 2021 | Effects of telehealth interventions for adolescent sexual health: A systematic review and meta-analysis of randomized controlled studies. | This study aimed to explore the meta-effects of telehealth interventions on self-efficacy of using condoms, condom use practices, and sexually transmitted infection testing behaviors among adolescents. | 15 | 5499 | 2021 | 1 January 2002 to 8 May 2021 | 10/15 USA 1 de cada Kenya, Hong Kong, Netherlands, Bolivia, and Palestine. | Yes | No | No | 15 |
| Schnall 2014 | eHealth interventions for HIV prevention in high-risk men who have sex with men: a systematic review. | to examine the use of eHealth interventions for HIV prevention in high-risk MSM. | 13 | not report | 2014 | from January 2000 to April 2014 | 9 USA, and the remaining studies were conducted in Peru (n=1), Australia (n=1), Taiwan (n=1), and Hong Kong (n=1). | No | Yes | No | 8 |
| Sewak 2023 | The effectiveness of digital sexual health interventions for young adults: a systematic literature review (2010-2020). | The aims of this systematic review study are threefold. First, to build on previous reviews (Salam et al., 2016) and reflect the current trends and practices of the safe-sexual health promotion sector when digitizing interventions. | 61 | not report | 2020 | 2010-2020 | USA (n = 32), the UK, Australia and Nigeria, with three studies from each country. Mexico, Sweden and South Africa contributed two studies each. Only one study was found for Argentina(1) y Chile (1), Colombia, Ghana, Hong Kong, Iran, the Netherlands , Portugal, Senegal, South Korea , Spain , Tajikistan, Tanzania and Uganda | No | No | Yes | 40 |
| Veronese 2020 | Using Digital Communication Technology to Increase HIV Testing Among Men Who Have Sex With Men and Transgender Women: Systematic Review and Meta-Analysis. | We undertook a systematic review and meta-analysis to assess the impact of digital communication technology on HIV testing uptake among MSM and transgender women (TW). Subanalyses aimed to identify the features and characteristics of digital interventions associated with greater impact. | 13 | 8875 | 2018 | January 1, 2010, and May 1, 2018 | the majority of studies took place in high-income countries (Hong Kong, n=1; Taiwan, n=1; and United States, n=6), 4 occurred in upper-middle-income countries (China, n=1 and Peru, n=3), and 1 in a low-middle-income country (India) | No | Yes | No | 10 |
| Xin 2020 | The Effectiveness of Electronic Health Interventions for Promoting HIV-Preventive Behaviors Among Men Who Have Sex With Men: Meta-Analysis Based on an Integrative Framework of Design and Implementation Features. | This study aimed to conduct a meta-analysis of the effectiveness of eHealth technology–based interventions for promoting HIV-preventive behaviors among MSM and to determine effectiveness predictors within a framework integrating design and implementation features. | 44 | 27704 | 2019 | 2006-2019 | Over half of the eligible programs (23/44, 52%) were conducted in the United States, and 10 and 8 programs were conducted in Asia and Europe, respectively | No | No | Yes |  |

Characterists Poputation

# Appendix 10 – Appendix 10. Mapping of Frequently Used Behavior Change Techniques (BCTs) and TDF Domains in Included Reviews

| **Author** | **Most Frequent BCTs and Components** | **TDF Domains (inferred or explicit)** | **BCT codification level** |
| --- | --- | --- | --- |
| Ashkanani et al., 2023 | 5.1 Information about health consequences; 1.1 Goal setting (behavior); 9.1 Credible source | Knowledge; Beliefs about consequences; Environmental context and resources; Social influences; Behavioral regulation | Explicit/inferable |
| Brandt et al., 2024 | 5.1 Information about health consequences; 4.2 Information about antecedents | Knowledge; Beliefs about consequences; Environmental context and resources | Limited – general strategies only |
| Gould et al., 2014 | 5.1 Information about health consequences; 1.1 Goal setting (behavior); 2.2 Feedback on behavior | Knowledge; Skills; Beliefs about capabilities; Social influences; Behavioral regulation | Limited – general strategies only |
| Lynch et al., 2020 | 7.1 Prompts/cues; 10.4 Social reward; 11.2 Reduce negative emotions | Knowledge; Emotion; Beliefs about consequences; Beliefs about capabilities; Reinforcement; Environmental context and resources; Social influences; Memory, attention and decision processes; Behavioral regulation | Explicit/inferable |
| Rodrigues Melo et al., 2023 | 5.1 Information about health consequences; 9.1 Credible source | Knowledge; Environmental context and resources; Social influences | Limited – general strategies only |
| Mugunthan et al., 2011 | 2.2 Feedback on behavior; 5.2 Salience consequences | Knowledge; Memory, attention and decision processes | Explicit/inferable |
| Niznik et al., 2022 | 5.1 Information about health consequences; 9.1 Credible source; 1.1 Goal setting (behavior) | Knowledge; Social/professional role and identity; Social influences; Environmental context and resources | Explicit/inferable |
| Paquin et al., 2014 | 1.1 Goal setting (behavior); 4.2 Information about antecedents; 5.1 Information about health consequences | Knowledge; Beliefs about consequences; Goals; Behavioral regulation | Explicit/inferable |
| Parr et al., 2009 | 10.1 Material incentive (behavior); 10.4 Social reward; 10.5 Self-incentive | Knowledge; Beliefs about consequences; Social influences | Explicit/inferable |
| Smith and Tett, 2010 | 1.1 Goal setting (behavior); 2.2 Feedback on behavior; 5.1 Information about health consequences | Knowledge; Social influences; Environmental context and resources; Behavioral regulation | Explicit/inferable |
| Soni et al., 2022 | 1.1 Goal setting (behavior); 1.9 Commitment; 5.1 Information about health consequences | Knowledge; Beliefs about consequences; Skills; Behavioral regulation | Explicit/inferable |
| Baandrup et al., 2018 | Not reported (pharmacological only) | Not applicable | Not applicable |
| Rasmussen et al., 2021 | Not reported (contextual only) | Not applicable | Not applicable |
| Welsh et al., 2018 | 5.1 Information about health consequences | Knowledge; Beliefs about consequences | Explicit/inferable |

# Appendix 11 – Critical assessments based on AMSTAR 2

| **AMSTAR 2** Shea BJ, Reeves BC, Wells G, Thuku M, Hamel C, Moran J, Moher D, Tugwell P, Welch V, Kristjansson E, Henry DA. AMSTAR 2: a critical appraisal tool for systematic reviews that include randomised or non-randomised studies of healthcare interventions, or both. BMJ. 2017 Sep 21;358:j4008. | |
| --- | --- |
|  | |
| **Rating Overall Confidence in the results of the review** | **AMSTAR 2  CRITICAL DOMAINS** |
| **High** No or one non-critical weakness: the systematic review provides an accurate and comprehensive summary of the results of the available studies that address the question of interest  **Moderate** More than one non-critical weakness*: the systematic review has more than one weakness but no critical flaws. It may provide an accurate summary of the results of the available studies that were included in the review  **Low** One critical flaw with or without non-critical weaknesses: the review has a critical flaw and may not provide an accurate and comprehensive summary of the available studies that address the question of interest  **Critically low** More than one critical flaw with or without non-critical weaknesses: the review has more than one critical flaw and should not be relied on to provide an accurate and comprehensive summary of the available studies *Multiple non-critical weaknesses may diminish confidence in the review and it may be appropriate to move the overall appraisal down from moderate to low confidence | **2.** Protocol registered before commencement of the review   **4.** Adequacy of the literature search   **7.** Justification for excluding individual studies   **9.** Risk of bias from individual studies being included in the review   **11.** Appropriateness of meta-analytical methods   **13.** Consideration of risk of bias when interpreting the results of the review  **15.** Assessment of presence and likely impact of publication bias |

# Appendix 12 – Overlap in primary studies included in reviews


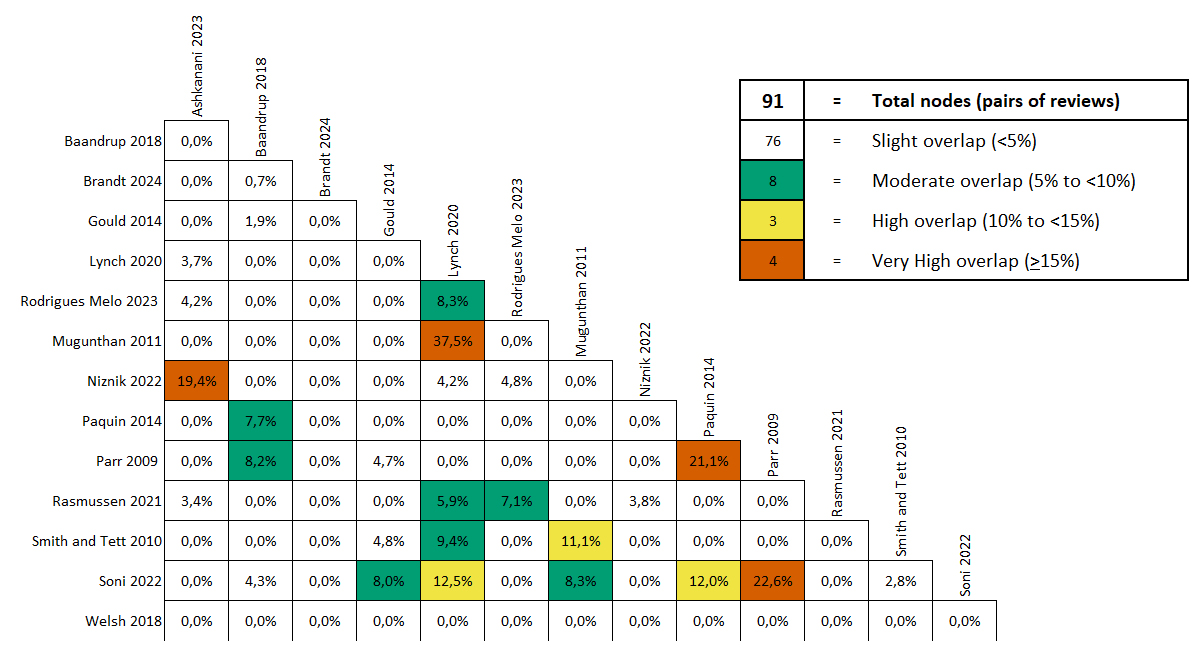


# Appendix 13. Matrix of evidence

| **Primary studies** | | | **Systematic Reviews** | | | | | | | | | | | | | |
| --- | --- | --- | --- | --- | --- | --- | --- | --- | --- | --- | --- | --- | --- | --- | --- | --- |
| **Nro** | **Author/year** | **Reference** | **Ashkanani 2023** | **Baandrup 2018** | **Brandt 2024** | **Gould 2014** | **Lynch 2020** | **Rodrigues Melo 2023** | **Mugunthan 2011** | **Niznik 2022** | **Paquin 2014** | **Parr 2009** | **Rasmussen 2021** | **Smith and Tett 2010** | **Soni 2022** | **Welsh 2018** |
| 1 | Aharaz et al., 2021 | Aharaz A, Rasmussen JH, McNulty HBO, et al. A collaborative deprescribing intervention in a subacute medical outpatient clinic: A pilot randomized controlled trial. Metabolites. 2021;11(4) | 1 |  |  |  |  |  |  |  |  |  |  |  |  |  |
| 2 | Ailabouni et al., 2019 | Ailabouni N, Mangin D, Nishtala PS. DEFEAT-polypharmacy: deprescribing anticholinergic and sedative medicines feasibility trial in residential aged care facilities. Int J Clin Pharm. 2019;41(1):167–178. | 1 |  |  |  |  |  |  |  |  |  |  |  |  |  |
| 3 | Monzón-Kenneke et al., 2021 | Monzón-Kenneke M, Chiang P, Yao NA, Greg M. Pharmacist medication review: an integrated team approach to serve home-based primary care patients. PLoS One. 2021;16(5), e0252151. | 1 |  |  |  |  |  |  |  |  |  |  |  |  |  |
| 4 | van der Meer et al., 2019 | van der Meer HG, Wouters H, Teichert M, et al. Feasibility, acceptability and potential effectiveness of an information technology-based, pharmacist-led intervention to prevent an increase in anticholinergic and sedative load among older community-dwelling individuals. Ther Adv Drug Safety. 2019;10:1–13. | 1 |  |  |  |  |  |  |  |  |  |  |  |  |  |
| 5 | Campbell et al., 2019 | Campbell NL, Perkins AJ, Khan BA, et al. Deprescribing in the pharmacologic Management of Delirium: a randomized trial in the intensive care unit. J Am Geriatr Soc. 2019;67(4):695–702. | 1 |  |  |  |  |  |  | 1 |  |  |  |  |  |  |
| 6 | Carr et al., 2019 | Carr F, Tian P, Chow J, et al. Deprescribing benzodiazepines among hospitalised older adults: quality improvement initiative. BMJ Open Qual. 2019;8(3), e000539. | 1 |  |  |  |  |  |  | 1 |  |  |  |  |  |  |
| 7 | Feldman et al., 2019 | Feldman EA, Noviasky J, Ulen KR, et al. Targeted medication Deprescribing in the elderly: results of a pharmacist-driven procedure in a transitional care unit. Sr Care Pharm. 2019;34(10):678–686. | 1 |  |  |  |  |  |  |  |  |  |  |  |  |  |
| 8 | Houlind et al., 2020 | Houlind MB, Andersen AL, Treldal C, et al. A collaborative medication review including deprescribing for older patients in an emergency department: A longitudinal feasibility study. J Clin Med. 2020;9(2) (no pagination). | 1 |  |  |  |  |  |  |  |  |  |  |  |  |  |
| 9 | Mudge et al., 2016 | Mudge A, Radnedge K, Kasper K, et al. Effects of a pilot multidisciplinary clinic for frequent attending elderly patients on deprescribing. Australian Health Rev A Publicat Australian Hosp Associat. 2016;40(1):86–91. | 1 |  |  |  |  |  |  |  |  |  |  |  |  |  |
| 10 | Shilpa et al., 2019 | Shilpa HSS, Kumar NN, Maheswari E, et al. Deprescribing of benzodiazepines and Z-drugs amongst the psychiatric patients of a tertiary care hospital. Asian J Psychiatr. 2019;44:189–194. | 1 |  |  |  |  |  |  |  |  |  |  |  |  |  |
| 11 | Wilson et al., 2018 | Wilson MG, Lee TC, Hass A, et al. EMPOWERing hospitalized older adults to deprescribe sedative hypnotics: a pilot study. J Am Geriatr Soc. 2018;66(6):1186–1189. | 1 |  |  |  |  |  |  | 1 |  |  |  |  |  |  |
| 12 | Lindsay et al., 2015 | Lindsay J, Dooley M, Martin J, et al. The development and evaluation of an oncological palliative care deprescribing guideline: the ‘OncPal deprescribing guideline’. Support Care Cancer. 2015;23(1):71–78. | 1 |  |  |  |  |  |  |  |  |  |  |  |  |  |
| 13 | Martin et al., 2018 | Martin P, Tamblyn R, Benedetti A, et al. Effect of a pharmacist-led educational intervention on inappropriate medication prescriptions in older adults: the D-PRESCRIBE randomized clinical trial. JAMA. 2018;320(18):1889–1898. | 1 |  |  |  |  |  |  | 1 |  |  |  |  |  |  |
| 14 | Tannenbaum et al., 2014 | Tannenbaum C, Martin P, Tamblyn R, et al. Reduction of inappropriate benzodiazepine prescriptions among older adults through direct patient education: the EMPOWER cluster randomized trial. JAMA Intern Med. 2014;174(6):890–898. | 1 |  |  |  | 1 | 1 |  | 1 |  |  | 1 |  |  |  |
| 15 | Turner et al., 2021 | Turner JP, Sanyal C, Martin P, et al. Economic evaluation of sedative deprescribing in older adults by community pharmacists. J Gerontol. 2021;76(6):1061–1067. | 1 |  |  |  |  |  |  |  |  |  |  |  |  |  |
| 16 | Lui et al., 2021 | Lui E, Wintemute K, Muraca M, et al. Pharmacist-led sedative-hypnotic deprescribing in team-based primary care practice. Canadian Pharmac J. 2021;154(4):278–284. | 1 |  |  |  |  |  |  |  |  |  |  |  |  |  |
| 17 | Westbury et al., 2018 | Westbury JL, Gee P, Ling T, et al. RedUSe: reducing antipsychotic and benzodiazepine prescribing in residential aged care facilities. Med J Aust. 2018;208(9):398–403. | 1 |  |  |  |  |  |  | 1 |  |  |  |  |  |  |
| 18 | Farrell et al., 2018 | Farrell B, Richardson L, Raman-Wilms L, et al. Self-efficacy for deprescribing: a survey for health care professionals using evidence-based deprescribing guidelines. Res Soc Administrat Pharm RSAP. 2018;14(1):18–25. | 1 |  |  |  |  |  |  |  |  |  |  |  |  |  |
| 19 | Blenke et al., 2018 | Blenke AA, Van Marum RJ, Windsant-Van Den Tweel AMV, et al. Deprescribing in newly admitted psychogeriatric nursing facility patients. Consult Pharm. 2018;33(6):331–338. | 1 |  |  |  |  |  |  |  |  |  |  |  |  |  |
| 20 | Whitman et al., 2018 | Whitman A, DeGregory K, Morris A, et al. Pharmacist-led medication assessment and deprescribing intervention for older adults with cancer and polypharmacy: a pilot study. Support Care Cancer. 2018;26(12):4105–4113. | 1 |  |  |  |  |  |  |  |  |  |  |  |  |  |
| 21 | Ashton 1990 | Ashton CH, Rawlins MD, Tyrer SP. A double-blind placebo-controlled study of buspirone in diazepam withdrawal in chronic benzodiazepine users. British Journal of Psychiatry 1990;157:232-8. |  | 1 |  |  |  |  |  |  |  |  |  |  |  |  |
| 22 | Baandrup 2016 | Baandrup L, Fagerlund B, Glenthoj B. Neurocognitive performance, subjective well-being, and psychosocial functioning after benzodiazepine withdrawal in patients with schizophrenia or bipolar disorder: a randomized clinical trial of add-on melatonin versus placebo. European Archives of Psychiatry and Clinical Neuroscience 2017;267(2):163-71. |  | 1 |  |  |  |  |  |  |  |  |  |  |  |  |
| 23 | Cassano 1996 | Cassano GB, Petracca A, Borghi C, Chiorri S, Didoni G, Garreau M. A randomized, double-blind study of alpidem vs placebo in the prevention and treatment of benzodiazepine withdrawal syndrome. European Psychiatry 1996;11(2):93-9. |  | 1 |  |  |  |  |  |  |  |  |  |  |  |  |
| 24 | Cialdella 2001 | Cialdella P, Boissel JP, Belon P, the ASTRHO group. Homeopathic specialties as a substitute for benzodiazepines: a double-blind vs. placebo study. Thérapie 2001;56:397-402. |  | 1 |  |  |  |  |  |  |  |  |  |  |  |  |
| 25 | Di Costanzo 1992 | Di Costanzo E, Rovea A. The prophylaxis of benzodiazepine withdrawal syndrome in the elderly: the effectiveness of carbamazepine. A double-blind study vs. placebo. Minerva Psichiatrica 1992;33:301-4. |  | 1 |  | 1 |  |  |  |  |  |  |  |  |  |  |
| 26 | Garfinkel 1999 | Garfinkel D, Zisapel N, Wainstein J, Laudon M. Facilitation of benzodiazepine discontinuation by melatonin: a new clinical approach. Archives of Internal Medicine 1999;159(20):2456-60. |  | 1 |  |  |  |  |  |  | 1 | 1 |  |  |  |  |
| 27 | Gerra 1993 | Gerra G, Marcato A, Caccavari R, Fontanan-QQin C, Fortunato A, Zaimovic A, et al. Effectiveness of flumazenil (Ro 15-1788) in the treatment of benzodiazepine withdrawal. Current Therapeutic Research - Clinical and Experimental 1993;54(5):580-7. |  | 1 |  |  |  |  |  |  |  |  |  |  |  |  |
| 28 | Gerra 2002 | Gerra G, Zaimovic A, Guisti M, Moi G, Brewer C. Intravenous flumazenil versus oxazepam tapering in the treatment of benzodiazepine withdrawal: a randomized, placebo-controlled study. Addiction Biology 2002;7:385-95. |  | 1 |  |  |  |  |  |  |  |  |  |  |  |  |
| 29 | GlaxoSmithKline 2002 | GlaxoSmithKline. The comparison of paroxetine and placebo on the symptoms emerging during the taper phase of a chronic benzodiazepine treatment: a randomised clinical trial of anxiety disorders. CSK - Clinical Study Register 2002. www.gsk-clinicalstudyregister.com. |  | 1 |  |  |  |  |  |  |  |  |  |  |  |  |
| 30 | Hadley 2012 | Hadley SJ, Mandel FS, Schweizer E. Switching from long-term benzodiazepine therapy to pregabalin in patients with generalized anxiety disorder: a double-blind, placebo-controlled trial. Journal of Psychopharmacology 2012;26(4):461-70. |  | 1 |  |  |  |  |  |  |  |  |  |  |  |  |
| 31 | Hantouche 1998 | Hantouche EG, Guelfi JD, Comet D. Discontinuation of long-term benzodiazepine use: double-blind controlled study of α-β L-aspartate magnesium versus placebo in 144 chronic users. L'Encéphale 1998;24:469-79. |  | 1 |  |  |  |  |  |  |  |  |  |  |  |  |
| 32 | Harrison-Read 1996 | Harrison-Read PE, Tyrer P, Lawson C, Kader I, File SE. Flumazenil-precipitated panic and dysphoria in patients dependent on benzodiazepines: a possible aid to abstinence. Journal of Psychopharmacology 1996;10:89-97. |  | 1 |  |  |  |  |  |  |  |  |  |  |  |  |
| 33 | Klein 1994 | Klein E, Colin V, Saksi K, Liron RH. Alprazolam withdrawal in patients with panic disorder and generalized anxiety disorder: a double-blind comparison. American Journal of Psychiatry 1994;151(12):1760-6. |  | 1 |  |  |  |  |  |  |  |  |  |  |  |  |
| 34 | Kornowski 2002 | Kornowski J. The comparison between tianeptine and carbamazepine in benzodiazepines withdrawal syndrome. Psychiatria Polska 2002;6(Suppl):311-8. |  | 1 |  |  |  |  |  |  |  |  |  |  |  |  |
| 35 | Lader 1987 | Lader M, Olajide D. A comparison of buspirone and placebo in relieving benzodiazepine withdrawal symptoms. Journal of Clinical Psychopharmacology 1987;7(1):11-5. |  | 1 |  |  |  |  |  |  |  |  |  |  |  |  |
| 36 | Lader 1993 | Lader M, Farr I, Morton S. A comparison of alpidem and placebo in relieving benzodiazepine withdrawal symptoms. International Clinical Psychopharmacology 1993;8(1):31-6. |  | 1 | 1 |  |  |  |  |  |  |  |  |  |  |  |
| 37 | Lecrubier 2005 | Lecrubier Y, Fessard N. Benzodiazepine discontinuation in chronic users: a double-blind trial of lithium gluconate vs placebo. Annales Medico Psychologiques 2005;163:24-9. |  | 1 |  |  |  |  |  |  |  |  |  |  |  |  |
| 38 | Lemoine 2006 | Lemoine P, Kermadi I, Garcia-Acosta S, Garay RP, Dib M. Double-blind, comparative study of cyamemazine vs. bromazepam in the benzodiazepine withdrawal syndrome. Progress in Neuro-Psychopharmacology & Biological Psychiatry 2006;30(1):131-7. |  | 1 |  |  |  |  |  |  |  |  |  |  |  |  |
| 39 | Mariani 2016 | Mariani JJ, Malcolm RJ, Mamczur AK, Choi JC, Brady R, Nunes E, et al. Pilot trial of gabapentin for the treatment of benzodiazepine abuse or dependence in methadone maintenance patients. American Journal of Drug and Alcohol Abuse 2016;42(3):333-40. [PUBMED: 26962719] |  | 1 |  |  |  |  |  |  |  |  |  |  |  |  |
| 40 | Mercier-Guyon 2004 | Mercier-Guyon C, Chabannes JP, Saviuc P. The role of captodiamine in the withdrawal from long-term benzodiazepine treatment. Current Medical Research and Opinion 2004;20(9):1347-55. |  | 1 |  |  |  |  |  |  |  |  |  |  |  |  |
| 41 | Morton 1995 | Morton S, Lader M. Buspirone treatment as an aid to benzodiazepine withdrawal. Journal of Psychopharmacology 1995;9(4):331-5. |  | 1 |  |  |  |  |  |  |  |  |  |  |  |  |
| 42 | Nakao 2006 | Nakao M, Takeuchi T, Nomura K, Teramoto T, Yano E. Clinical application of paroxetine for chronic benzodiazepine users at an internal medicine clinic. Therapeutic Research 2006;27(5):859-67. |  | 1 |  |  |  |  |  |  | 1 | 1 |  |  | 1 |  |
| 43 | Pat-Horenczyk 1998 | Pat-Horenczyk R, Hacohen D, Herer P, Lavie P. The eQects of substituting zopiclone in withdrawal from chronic use of benzodiazepine hypnotics. Psychopharmacology 1998;140(4):450-7. [DOI: 10.1007/s002130050789] |  | 1 |  |  |  |  |  |  |  |  |  |  |  |  |
| 44 | Peles 2007 | Peles E, Hetzroni T, Bar-Hamburger R, Adelson M, Schreiber S. Melatonin for perceived sleep disturbances associated with benzodiazepine withdrawal among patients in methadone maintenance treatment: a double-blind randomized clinical trial. Addiction 2007;102(12):1947-53. |  | 1 |  |  |  |  |  |  |  |  |  |  |  |  |
| 45 | Rickels 1999 | Rickels K, Schweizer E, Garcia-Espana F, Case G, DeMartinis N, Greenblatt D. Trazodone and valproate in patients discontinuing long-term benzodiazepine therapy: effects on withdrawal symptoms and taper outcome. Psychopharmacology 1999;141(1):1-5. |  | 1 |  |  |  |  |  |  | 1 | 1 |  |  |  |  |
| 46 | Rickels 2000 | Rickels K, Schweizer E, Garcia Espana F, Case, G, DeMartinis N, Greenblatt D. Trazodone and valproate in patients discontinuing long-term benzodiazepine therapy: eQects on withdrawal symptoms and taper outcome. Psychopharmacology 1999;141(1):1-5. |  | 1 |  |  |  |  |  |  |  |  |  |  |  |  |
| 47 | Romach 1998 | Romach MK, Kaplan HL, Busto UE, Somer G, Sellers EM. A controlled trial of ondansetron, a 5-HT3 antagonist, in benzodiazepine discontinuation. Journal of Clinical Psychopharmacology 1998;18(2):121-31. |  | 1 |  |  |  |  |  |  |  |  |  |  |  |  |
| 48 | Rynn 2003 | Rynn M, Garcia-Espana F, Greenblatt DJ, Mandos LA, Schweizer E, Rickels K. Imipramine and buspirone in patients with panic disorder who are discontinuing long-term benzodiazepine therapy. Journal of Clinical Psychopharmacology 2003;23(5):505-8. |  | 1 |  |  |  |  |  |  |  |  |  |  |  |  |
| 49 | Saul 1989 | Saul PA, Korlipara K, Presley P. A randomised, multicentre, double-blind, comparison of atenolol and placebo in the control of benzodiazepine withdrawal symptoms. Acta Therapeutica 1989;15(2):117-23. |  | 1 |  |  |  |  |  |  |  |  |  |  |  |  |
| 50 | Schweizer 1991 | Schweizer E, Rickels, K, Case WG, Greenblatt DJ. Carbamazepine treatment in patients discontinuing long-term benzodiazepine therapy. EQects on withdrawal severity and outcome. Archives of General Psychiatry 1991;48(5):448-52. |  | 1 |  |  |  |  |  |  |  |  |  |  |  |  |
| 51 | Schweizer 1995 | Schweizer E, Case WG, Garcia-Espana F, Greenblatt DJ, Rickels K. Progesterone co-administration in patients discontinuing long-term benzodiazepine therapy: effects on withdrawal severity and outcome. Psychopharmacology 1995;117(4):424-9. |  | 1 |  |  |  |  |  |  |  |  |  |  |  |  |
| 52 | Tyrer 1981 | Tyrer P, Rutherford D, Huggett T. Benzodiazepine withdrawal symptoms and propranolol. Lancet 1981;1(8221):520-2. |  | 1 |  |  |  |  |  |  |  |  |  |  |  |  |
| 53 | Tyrer 1996 | Tyrer P, Ferguson B, Hallstrom C, Michie M, Tyrer S, Cooper S, et al. A controlled trial of dothiepin and placebo in treating benzodiazepine withdrawal symptoms. British Journal of Psychiatry 1996;168(4):457-61. |  | 1 |  |  |  |  |  |  |  |  |  |  |  |  |
| 54 | Udelman 1990 | Udelman HD, Udelman DL. Concurrent use of buspirone in anxious patients during withdrawal from alprazolam therapy. Journal of Clinical Psychiatry 1990;51 Suppl:46-50. |  | 1 |  |  |  |  |  |  | 1 | 1 |  |  |  |  |
| 55 | Vissers 2007 | Vissers FH, Knipschild PG, Crebolder HF. Is melatonin helpful in stopping the long-term use of hypnotics? A discontinuation trial. Pharmacy World & Science 2007;29(6):641-6. [DOI: 10.1007/s11096-007-9118-y] |  | 1 |  |  |  |  |  |  |  |  |  |  |  |  |
| 56 | Vorma 2011 | Vorma H, Katila H. Effect of valproate on benzodiazepine withdrawal severity in opioid-dependent subjects: a pilot study. Heroin Addiction and Related Clinical Problems 2011;13(1):15-20. |  | 1 |  |  |  |  |  |  |  |  |  |  |  |  |
| 57 | Zhang 2013 | Zhang H, Jiang X, Ma M, Zhang J. A control study on treatment for benzodiazepine dependence with trazodone. Chinese Journal of Contemporary Neurology and Neurosurgery 2013;13(5):411-5. |  | 1 |  |  |  |  |  |  |  | 1 |  |  |  |  |
| 58 | Zitman 2001 | Zitman FG, Couvee JE. Chronic benzodiazepine use in general practice patients with depression: an evaluation of controlled treatment and taper-ex protocol on behalf of the Dutch Chronic Benzodiazepine Working Group. British Journal of Psychiatry 2001;178:317-24. |  | 1 |  |  |  |  |  |  |  |  |  |  | 1 |  |
| 59 | Eleftheriou et al., 2023 | Consensus panel recommendations for the pharmacological management of pregnant women with depressive disorders. Int J Environ Res Publ Health. 2023;20(16):6565. [DOI](https://doi.org/10.3390/ijerph2016 6565) |  |  | 1 |  |  |  |  |  |  |  |  |  |  |  |
| 60 | Ilješ et al., 2023 | Recommendations for treatment of unipolar depressive disorder. Slov Med J. 2023:1–6. DOI |  |  | 1 |  |  |  |  |  |  |  |  |  |  |  |
| 61 | Miller et al., 2023 | Treatment and management of mental health conditions during pregnancy and postpartum: ACOG clinical practice guideline No. 5. Obstet Gynecol. 2023;141(6):1262–1288. DOI |  |  | 1 |  |  |  |  |  |  |  |  |  |  |  |
| 62 | Mula et al., 2022 | ILAE clinical practice recommendations for the medical treatment of depression in adults with epilepsy. Epilepsia. 2022;63(2):316–334. DOI |  |  | 1 |  |  |  |  |  |  |  |  |  |  |  |
| 63 | Voytenko et al., 2018 | Evidence-Based practice guideline for the treatment of adult patients with depressive disorders. Part I: psychiatric management. Psychiatr i Psychol Klin. 2018;18(3):234–241. DOI |  |  | 1 |  |  |  |  |  |  |  |  |  |  |  |
| 64 | Piotrowski et al., 2017 | Guidelines of the polish psychiatric association–wroclaw division, the polish society of family medicine and the college of family physicians in Poland for diagnosis and treatment of depressive disorders in primary health care. Fam Med Prim Care Rev. 2017;(3):335–346. DOI |  |  | 1 |  |  |  |  |  |  |  |  |  |  |  |
| 65 | Kennedy et al., 2016 | CANMAT 2016 clinical guidelines for the management of adults with major depressive disorder: section 3. Pharmacological treatments. Can J Psychiatr. 2016;61(9):540–560. DOI |  |  | 1 |  |  |  |  |  |  |  |  |  |  |  |
| 66 | Bauer et al., 2013 | World Federation of Societies of Biological Psychiatry (WFSBP) guidelines for biological treatment of unipolar depressive disorders, part 1: update 2013 on the acute and continuation treatment of unipolar depressive disorders. World J Biol Psychiatr. 2013;14(5):334–385. DOI |  |  | 1 |  |  |  |  |  |  |  |  |  |  |  |
| 67 | Bauer et al., 2015 | WFSBP guidelines for biological treatment of unipolar depressive disorders. Part 2: maintenance treatment of major depressive disorder-update 2015. World J Biol Psychiatr. 2015;16(2):76–95. DOI |  |  | 1 |  |  |  |  |  |  |  |  |  |  |  |
| 68 | Austin et al., 2013 | Detection and management of mood disorders in the maternity setting: the Australian Clinical Practice Guidelines. Women Birth. 2013;26(1):29. DOI |  |  | 1 |  |  |  |  |  |  |  |  |  |  |  |
| 69 | Chua et al., 2012 | Ministry of Health clinical practice guidelines: depression. Singap Med J. 2012;53(2):137–144. |  |  | 1 |  |  |  |  |  |  |  |  |  |  |  |
| 70 | Malhi et al., 2009 | Clinical practice recommendations for depression. Acta Psychiatr Scand. 2009;119:8–26. DOI |  |  | 1 |  |  |  |  |  |  |  |  |  |  |  |
| 71 | Fleck et al., 2009 | Review of the guidelines of the Brazilian Medical Association for the treatment of depression (Full version). Br J Psychiatry. 2009;31:S7–S17. DOI |  |  | 1 |  |  |  |  |  |  |  |  |  |  |  |
| 72 | Anderson 2001 | Evidence-based guidelines for treating depressive disorders with antidepressants: a revision of the 1993 British Association for Psychopharmacology guidelines. J Psychopharmacol. 2000;14(1):3–20. DOI |  |  | 1 |  |  |  |  |  |  |  |  |  |  |  |
| 73 | Conn 2006 | National guidelines for seniors’ mental health: the assessment and treatment of mental health issues in long-term care homes (focus on mood and behaviour symptoms). Toronto, ON: Canadian Coalition for Seniors’ Mental Health; 2006:1–56. PDF |  |  | 1 |  |  |  |  |  |  |  |  |  |  |  |
| 74 | Rosenbluth 2012 | The Canadian Network for Mood and Anxiety Treatments (CANMAT) task force recommendations for the management of patients with mood disorders and comorbid personality disorders. Ann Clin Psychiatr. 2012;24(1):56–68 |  |  | 1 |  |  |  |  |  |  |  |  |  |  |  |
| 75 | Dodd 2011 | A consensus statement for safety monitoring guidelines of treatments for major depressive disorder. Aust N Z J Psychiatry. 2011;45(9):712–725. DOI |  |  | 1 |  |  |  |  |  |  |  |  |  |  |  |
| 76 | Motohashi 2008 | Revised psychopharmacological algorithms for the treatment of mood disorders in Japan. Int J Psychiatr Clin Pract. 2008;12(1):11–18. DOI |  |  | 1 |  |  |  |  |  |  |  |  |  |  |  |
| 77 | Romeijnders 2005 | [Summary of the standard ’depressive disorder’ (first revision) of the Dutch society of general practitioners]. Ned Tijdschr Geneeskd. 2005;149:523–527 |  |  | 1 |  |  |  |  |  |  |  |  |  |  |  |
| 78 | Crismon 1999 | The Texas medication algorithm project: report of the Texas consensus conference panel on medication treatment of major depressive disorder. JClin Psychiatr. 1999;60(3):142–156. DOI |  |  | 1 |  |  |  |  |  |  |  |  |  |  |  |
| 79 | Doctors of B.C 2013 | Major depressive disorder in adults: diagnosis & management; 2013:1–6. PDF (Accessed September 2023) |  |  | 1 |  |  |  |  |  |  |  |  |  |  |  |
| 80 | Ministry of Health Malaysia 2019 | Management of major depressive disorder. 2nd ed.; 2019:1–66. PDFCPG_Management_Major_Depressive_Disorder(Second_Edition).pdf) (Accessed September 2023) |  |  | 1 |  |  |  |  |  |  |  |  |  |  |  |
| 81 | Trangle 2016 | Institute for clinical system improvement. Adult depression in primary care: healthcare guideline. Bloomington, MN; 2016:1–131. PDF (Accessed September 2023) |  |  | 1 |  |  |  |  |  |  |  |  |  |  |  |
| 82 | Scottish Intercollegiate Guidelines Network 2012 | Sign 127: management of perinatal mood disorders; 2012:1–47. PDF (Accessed September 2023) |  |  | 1 |  |  |  |  |  |  |  |  |  |  |  |
| 83 | Claassen 2022 | [NHG-Standaard depressie (M44)]; 2022:1–113. Link (Accessed September 2023) |  |  | 1 |  |  |  |  |  |  |  |  |  |  |  |
| 84 | Finnish Medical Society Duocedim 2023 | Depression: good medical practice. Helsinki; 2023. Link (Accessed September 2023) |  |  | 1 |  |  |  |  |  |  |  |  |  |  |  |
| 85 | Schaffer 2012 | The Canadian Network for Mood and Anxiety Treatments (CANMAT) task force recommendations for the management of patients with mood disorders and comorbid anxiety disorders. Ann Clin Psychiatr. 2012;24(1):6–22 |  |  | 1 |  |  |  |  |  |  |  |  |  |  |  |
| 86 | Fuchs 2000 | Guidelines for the treatment of depression. Rev Med Liege. 2000;55(5):389–394 |  |  | 1 |  |  |  |  |  |  |  |  |  |  |  |
| 87 | Andrews 2018 | Royal Australian and New Zealand College of Psychiatrists clinical practice guidelines for the treatment of panic disorder, social anxiety disorder and generalised anxiety disorder. Aust N Z J Psychiatr. 2018;52(12):1109–1172. DOI |  |  | 1 |  |  |  |  |  |  |  |  |  |  |  |
| 88 | Subramanyam 2018 | Clinical practice guidelines for geriatric anxiety disorders. Indian J Psychiatr. 2018;60(Suppl 3):S371–S382. DOI |  |  | 1 |  |  |  |  |  |  |  |  |  |  |  |
| 89 | Gautam 2017 | Clinical practice guidelines for the management of generalised anxiety disorder (GAD) and panic disorder (PD). Indian J Psychiatr. 2017;59(Suppl 1):S67–S73. DOI |  |  | 1 |  |  |  |  |  |  |  |  |  |  |  |
| 90 | Bandelow 2022 | The German Guidelines for the treatment of anxiety disorders: first revision. Eur Arch Psychiatr Clin Neurosci. 2022;272(4):571–582. DOI |  |  | 1 |  |  |  |  |  |  |  |  |  |  |  |
| 91 | Katzman 2014 | Canadian clinical practice guidelines for the management of anxiety, posttraumatic stress and obsessive-compulsive disorders. BMC Psychiatry. 2014;14:1–83. DOI |  |  | 1 |  |  |  |  |  |  |  |  |  |  |  |
| 92 | Baldwin 2014 | Evidence-based pharmacological treatment of anxiety disorders, PTSD, and OCD: a revision of the 2005 guidelines from the British Association for Psychopharmacology. J Psychopharmacol. 2014;28(5):403–439. DOI |  |  | 1 |  |  |  |  |  |  |  |  |  |  |  |
| 93 | NICE 2011 | Generalised anxiety disorder and panic disorder in adults: management. NICE Clinical Guideline 113; 2011:1–47. Link (Accessed September 2023) |  |  | 1 |  |  |  |  |  |  |  |  |  |  |  |
| 94 | Guideline working group 2008 | Clinical Practice Guidelines in the NHS. UETS N◦ 2006/10. Link (Accessed September 2023) |  |  | 1 |  |  |  |  |  |  |  |  |  |  |  |
| 95 | Heggie 2018 | Magellan’s clinical practice guideline for the assessment and treatment of generalized anxiety disorder in adults; 2018:1–62. Link (Accessed September 2023) |  |  | 1 |  |  |  |  |  |  |  |  |  |  |  |
| 96 | Finnish Medical Society Duocedim 2019 | Anxiety disorders. Current care guidelines; 2019. Link (Accessed September 2023) |  |  | 1 |  |  |  |  |  |  |  |  |  |  |  |
| 97 | NHG-Working Group 2019 | [NHG-Standard anxiety (M62)]; 2019:1–73. Link (Accessed September 2023) |  |  | 1 |  |  |  |  |  |  |  |  |  |  |  |
| 98 | Haute Autorité de Santé 2017 | [Acts and services for long-term conditions: serious anxiety disorders]; 2017. Link (Accessed September 2023) |  |  | 1 |  |  |  |  |  |  |  |  |  |  |  |
| 99 | Brackett 2019 | Dartmouth-hitchcock. Clinical practice guideline: management of anxiety in adults in primary care; 2019:1–7. Link (Accessed September 2023) |  |  | 1 |  |  |  |  |  |  |  |  |  |  |  |
| 100 | Linden 2013 | The best next drug in the course of generalized anxiety disorders: the “PN-GAD-algorithm”. Int J Psychiatr Clin Pract. 2013;17(2):78–89. DOI |  |  | 1 |  |  |  |  |  |  |  |  |  |  |  |
| 101 | Abejuela 2016 | The psychopharmacology algorithm project at the Harvard South Shore Program: an algorithm for generalized anxiety disorder. Harv Rev Psychiatr. 2016;24(4):243-256. DOI |  |  | 1 |  |  |  |  |  |  |  |  |  |  |  |
| 102 | Davidson 2010 | A psychopharmacological treatment algorithm for generalised anxiety disorder (GAD). J Psychopharmacol. 2010;24(1):3–26. DOI |  |  | 1 |  |  |  |  |  |  |  |  |  |  |  |
| 103 | Allgulander 2003 | WCA recommendations for the long-term treatment of generalized anxiety disorder. CNS Spectr. 2003;8(S1):53–61. |  |  | 1 |  |  |  |  |  |  |  |  |  |  |  |
| 104 | Bandelow 2023 | World Federation of Societies of Biological Psychiatry (WFSBP) guidelines for treatment of anxiety, obsessive-compulsive, and posttraumatic stress disorders–Version 3. Part I: anxiety disorders. World J Biol Psychiatr. 2023;24(2):79–117. DOI |  |  | 1 |  |  |  |  |  |  |  |  |  |  |  |
| 105 | Yoon 2018 | Korean guidelines for the pharmacological treatment of social anxiety disorder: initial treatment strategies. Psychiatry Investig. 2018;15(2):147–155. DOI |  |  | 1 |  |  |  |  |  |  |  |  |  |  |  |
| 106 | Levitan 2011 | Guidelines of the Brazilian Medical Association for the treatment of social anxiety disorder. Br J Psychiatry. 2011;33:292–302. DOI |  |  | 1 |  |  |  |  |  |  |  |  |  |  |  |
| 107 | Stein 2010 | A 2010 evidence-based algorithm for the pharmacotherapy of social anxiety disorder. Curr Psychiatr Rep. 2010;12:471–477. DOI |  |  | 1 |  |  |  |  |  |  |  |  |  |  |  |
| 108 | Van Ameringen 2003 | WCA recommendations for the long-term treatment of social phobia. CNS Spectr. 2003;8(S1):40–52. |  |  | 1 |  |  |  |  |  |  |  |  |  |  |  |
| 109 | Ballenger 1998 | Consensus statement on social anxiety disorder from the international consensus group on depression and anxiety. J Clin Psychiatr. 1998;59:54–60. |  |  | 1 |  |  |  |  |  |  |  |  |  |  |  |
| 110 | Stein 2001 | Pharmacotherapy of social anxiety disorder: an algorithm for primary care–2001. Prim Care Psychiatr. 2001;7(3):107–110. DOI |  |  | 1 |  |  |  |  |  |  |  |  |  |  |  |
| 111 | National Collaborating Centre 2013 | Social anxiety disorder: the NICE guideline on recognition, assessment and treatment. British Psychological Society & Royal College of Psychiatrists; 2013:1–320. ISBN-: 978-1-909726-03-1. PDF |  |  | 1 |  |  |  |  |  |  |  |  |  |  |  |
| 112 | Stein 2009 | Practice guideline for the treatment of patients with panic disorder (2nd ed.). Am Psychiatr Assoc. 2009;166(2):1–90. DOI |  |  | 1 |  |  |  |  |  |  |  |  |  |  |  |
| 113 | Pollack 2003 | WCA recommendations for the long-term treatment of panic disorder. CNS Spectr. 2003;8(S1):17–30. |  |  | 1 |  |  |  |  |  |  |  |  |  |  |  |
| 114 | Roy-Byrne 1998 | Pharmacotherapy of panic disorder: proposed guidelines for the family physician. J Am Board Fam Pract. 1998;11(4):282–290. |  |  | 1 |  |  |  |  |  |  |  |  |  |  |  |
| 115 | Ballenger 1998 | Consensus statement on panic disorder from the international consensus group on depression and anxiety. J Clin Psychiatr. 1998;59:47–54. |  |  | 1 |  |  |  |  |  |  |  |  |  |  |  |
| 116 | Bandelow 2023 | World Federation of Societies of Biological Psychiatry (WFSBP) guidelines for treatment of anxiety, obsessive-compulsive and posttraumatic stress disorders–Version 3. Part II: OCD and PTSD. World J Biol Psychiatr. 2023;24(2):118–134. DOI |  |  | 1 |  |  |  |  |  |  |  |  |  |  |  |
| 117 | Reddy 2017 | Clinical practice guidelines for obsessive-compulsive disorder. Indian J Psychiatr. 2017;59(S1):S74–S90. DOI |  |  | 1 |  |  |  |  |  |  |  |  |  |  |  |
| 118 | de Oliveira 2023 | Brazilian Research Consortium on Obsessive-Compulsive Spectrum Disorders guidelines for the treatment of adult obsessive-compulsive disorder. Part I: pharmacological treatment. Br J Psychiatry. 2023;45:146–161. DOI |  |  | 1 |  |  |  |  |  |  |  |  |  |  |  |
| 119 | Koran 2013 | Guideline watch (March 2013): practice guideline for the treatment of patients with obsessive-compulsive disorder. Arlington, TX, USA: American Psychiatric Association Practice Guidelines; 2013:1–22. |  |  | 1 |  |  |  |  |  |  |  |  |  |  |  |
| 120 | Greist 2003 | WCA recommendations for the long-term treatment of obsessive-compulsive disorder in adults. CNS Spectr. 2003;8(S1):7–16. DOI |  |  | 1 |  |  |  |  |  |  |  |  |  |  |  |
| 121 | Stein 2012 | A 2012 evidence-based algorithm for the pharmacotherapy for obsessive-compulsive disorder. Curr Psychiatr Rep. 2012;14:211–219. DOI |  |  | 1 |  |  |  |  |  |  |  |  |  |  |  |
| 122 | Forbes 2007 | Australian guidelines for the treatment of adults with acute stress disorder and post-traumatic stress disorder. Aust N Z J Psychiatr. 2007;41(8):637–648. DOI |  |  | 1 |  |  |  |  |  |  |  |  |  |  |  |
| 123 | Ursano 2010 | Practice guideline for the treatment of patients with acute stress disorder and posttraumatic stress disorder. Washington, DC. USA: American Psychiatric Association Practice Guidelines; 2010:1–95. |  |  | 1 |  |  |  |  |  |  |  |  |  |  |  |
| 124 | Foa 2000 | Guidelines for treatment of PTSD. J Trauma Stress. 2000;13(4):539–588. DOI |  |  | 1 |  |  |  |  |  |  |  |  |  |  |  |
| 125 | Stein 2003 | WCA Recommendations for the long-term treatment of posttraumatic stress disorder. CNS Spectr. 2003;8(S1):31–39. DOI |  |  | 1 |  |  |  |  |  |  |  |  |  |  |  |
| 126 | National Center for PTSD 2013 | Helping patients taper from benzodiazepines; 2013:1–2. PDF (Accessed September 2023) |  |  | 1 |  |  |  |  |  |  |  |  |  |  |  |
| 127 | Watson 2023 | Alliance for sleep clinical practice guideline on switching or deprescribing hypnotic medications for insomnia. J Clin Med. 2023;12(7):1–22. DOI |  |  | 1 |  |  |  |  |  |  |  |  |  |  |  |
| 128 | Wichniak 2023 | Treatment of insomnia in older adults. Recommendations of the polish sleep research society, polish society of family medicine and the polish psychiatric association. Psychiatr Pol. 2023;57(3). DOI |  |  | 1 |  |  |  |  |  |  |  |  |  |  |  |
| 129 | Mysliwiec 2020 | The management of chronic insomnia disorder and obstructive sleep apnea: synopsis of the 2019 US Department of Veterans Affairs and US Department of Defense clinical practice guidelines. Ann Intern Med. 2020;172(5):325–336. DOI |  |  | 1 |  |  |  |  |  |  |  |  |  |  |  |
| 130 | Palagini 2020 | Expert opinions and consensus recommendations for the evaluation and management of insomnia in clinical practice: joint statements of five Italian scientific societies. Front Psychiatr. 2020;11:558. DOI |  |  | 1 |  |  |  |  |  |  |  |  |  |  |  |
| 131 | Choi 2020 | Korean clinical practice guideline for the diagnosis and treatment of insomnia in adults. Psychiatry Investig. 2020;17(11):1048–1059. DOI |  |  | 1 |  |  |  |  |  |  |  |  |  |  |  |
| 132 | Wilson 2019 | British Association for Psychopharmacology consensus statement on evidence-based treatment of insomnia, parasomnias and circadian rhythm disorders: an update. J Psychopharmacol. 2019;33(8):923–947. DOI |  |  | 1 |  |  |  |  |  |  |  |  |  |  |  |
| 133 | Praharaj 2018 | Clinical practice guideline on management of sleep disorders in the elderly. Indian J Psychiatr. 2018;60(S3):S383–S396. DOI |  |  | 1 |  |  |  |  |  |  |  |  |  |  |  |
| 134 | Pottie 2018 | Deprescribing benzodiazepine receptor agonists: evidence-based clinical practice guideline. Can Fam Physician. 2018;64(5):339–351. Full Text |  |  | 1 |  |  |  |  |  |  |  |  |  |  |  |
| 135 | Sateia 2017 | Clinical practice guideline for the pharmacologic treatment of chronic insomnia in adults: an American Academy of Sleep Medicine clinical practice guideline. J Clin Sleep Med. 2017;13(2):307–349. DOI |  |  | 1 |  |  |  |  |  |  |  |  |  |  |  |
| 136 | Riemann 2017 | European guideline for the diagnosis and treatment of insomnia. J Sleep Res. 2017;26(6):675–700. DOI |  |  | 1 |  |  |  |  |  |  |  |  |  |  |  |
| 137 | Gupta 2017 | Clinical practice guidelines for sleep disorders. Indian J Psychiatr. 2017;59(S1):S116–S138. DOI |  |  | 1 |  |  |  |  |  |  |  |  |  |  |  |
| 138 | Qaseem 2016 | Clinical Guidelines Committee of the American College of Physicians. Management of chronic insomnia disorder in adults: a clinical practice guideline from the American College of Physicians. Ann Intern Med. 2016;165(2):125–133. DOI |  |  | 1 |  |  |  |  |  |  |  |  |  |  |  |
| 139 | Medina-Chávez 2014 | Clinical practice guideline. Diagnosis and treatment of insomnia in the elderly. Rev Méd Inst Mex Seguro Soc. 2014;52(1):108–119. |  |  | 1 |  |  |  |  |  |  |  |  |  |  |  |
| 140 | Pinto 2010 | New guidelines for diagnosis and treatment of insomnia. Arq Neuropsiquiatr. 2010;68(4):666–675. |  |  | 1 |  |  |  |  |  |  |  |  |  |  |  |
| 141 | Mayer 2009 | S3-Guideline. [Non restorative sleep⁄ sleep disorders. German Sleep Society]. Somnologie. 2009;13(S1):4–160. DOI |  |  | 1 |  |  |  |  |  |  |  |  |  |  |  |
| 142 | Bloom 2009 | Evidence-based recommendations for the assessment and management of sleep disorders in older persons. J Am Geriatr Soc. 2009;57(5):761–789. DOI |  |  | 1 |  |  |  |  |  |  |  |  |  |  |  |
| 143 | Terzano 2005 | Insomnia in general practice: a consensus report produced by sleep specialists and primary-care physicians in Italy. Clin Drug Invest. 2005;25:745–764. DOI |  |  | 1 |  |  |  |  |  |  |  |  |  |  |  |
| 144 | Estivill 2003 | Consensus on drug treatment, definition and diagnosis for insomnia. Clin Drug Invest. 2003;23(6):351–385. DOI |  |  | 1 |  |  |  |  |  |  |  |  |  |  |  |
| 145 | Roth 2001 | Consensus for the pharmacological management of insomnia in the new millennium. Int J Clin Pract. 2001;55(1):42–52. |  |  | 1 |  |  |  |  |  |  |  |  |  |  |  |
| 146 | Yakabowich 1992 | Hypnotics in the elderly: appropriate usage guidelines. J Geriatr Drug Ther. 1992;6(3):5–21. DOI |  |  | 1 |  |  |  |  |  |  |  |  |  |  |  |
| 147 | Alberta Doctors 2015 | Towards optimized practice: assessment to management of adult insomnia; 2015:1–35. Link |  |  | 1 |  |  |  |  |  |  |  |  |  |  |  |
| 148 | Damen-van Beek 2015 | [The NHG guideline ’Sleep problems and sleeping pills’]. Ned Tijdschr Geneeskd. 2015; 159:A8679. |  |  | 1 |  |  |  |  |  |  |  |  |  |  |  |
| 149 | Finnish Medical Society Duocedim 2023 | Insomnia. Valid treatment recommendation; 2023. Enlace. Accessed September 2023. |  |  | 1 |  |  |  |  |  |  |  |  |  |  |  |
| 150 | Cloetens 2018 | First-line treatment of sleep disorders and insomnia in adults; 2018:1–10. [Enlace](https://cdn.nimbu.io/s/yba55wt/assets/WG Sleep disorders.pdf). Accessed September 2023. |  |  | 1 |  |  |  |  |  |  |  |  |  |  |  |
| 151 | Agoritsas 2023 | BE-SAFE: deprescription of benzodiazepine and sedative hypnotics (BSHs) in insomnia disorder. MAGIC Evidence Ecosystem Foundation; 2023. Enlace. Accessed December 29, 2023. |  |  | 1 |  |  |  |  |  |  |  |  |  |  |  |
| 152 | Benzodiazepine action working group 2022 | Benzodiazepine Deprescribing Guidance; 2022. Enlace. |  |  | 1 |  |  |  |  |  |  |  |  |  |  |  |
| 153 | National Institute for Health and Care Excellence 2022 | Medicines associated with dependence or withdrawal symptoms: safe prescribing and withdrawal management for adults [NG215]; 2022:1–41. Enlace. Accessed September 2023. |  |  | 1 |  |  |  |  |  |  |  |  |  |  |  |
| 154 | Sparks 2022 | Benzodiazepine and Z-drug safety guideline; 2022:1–26. Enlace. Accessed September 2023. |  |  | 1 |  |  |  |  |  |  |  |  |  |  |  |
| 155 | Barry 2021 | Guidance on appropriate prescribing of benzodiazepines and Z-drugs (BZRA) for the treatment of anxiety and insomnia; 2021:1–48. Enlace. Accessed 2023. |  |  | 1 |  |  |  |  |  |  |  |  |  |  |  |
| 156 | Conn 2020 | Canadian guidelines on benzodiazepine receptor agonist use disorder among older adults. Can Geriatr J. 2020;23(1):116–122. DOI. |  |  | 1 |  |  |  |  |  |  |  |  |  |  |  |
| 157 | Amanti 2018 | Benzodiazepine taper guidelines for older adults in an inpatient geiatric/psychiatric unit. Dissertation. University of Arizona; 2018:1–97. Enlace. Accessed 2023. |  |  | 1 |  |  |  |  |  |  |  |  |  |  |  |
| 158 | New Mexico Overdose Prevention and Pain Management Advisory Council 2018 | Guidelines for the use of benzodiazepines in the state of New Mexico; 2018:1–7. Enlace. Accessed September 2023. |  |  | 1 |  |  |  |  |  |  |  |  |  |  |  |
| 159 | Royal Australian College of General Practitioners 2015 | Prescribing drugs of dependence in general practice, Part B—benzodiazepines. Melbourne; 2015:1–85. Enlace. Accessed September 2023. |  |  | 1 |  |  |  |  |  |  |  |  |  |  |  |
| 160 | Haute Autorite de Sante 2015 | Guidance leaflet–discontinuation of benzodiazepines and related medicinal products: procedure for the doctor providing outpatient treatment; 2015:1–5. Enlace. Accessed September 2023. |  |  | 1 |  |  |  |  |  |  |  |  |  |  |  |
| 161 | JPS Health Network 2014 | Guidelines for prescribing and tapering benzodiazepines in an outpatient setting; 2014:1–11. Enlace. Accessed September 2023. |  |  | 1 |  |  |  |  |  |  |  |  |  |  |  |
| 162 | College of Psychiatry of Ireland 2012 | A consensus statement on the use of benzodiazepines in specialist mental health services. EAP Position Paper; 2012:1–9. Enlace. Accessed September 2023. |  |  | 1 |  |  |  |  |  |  |  |  |  |  |  |
| 163 | Lee 2008 | For Ministry of Health Singapore. Prescribing of benzodiazepines; 2008:1–57. Enlace. Accessed September 2023. |  |  | 1 |  |  |  |  |  |  |  |  |  |  |  |
| 164 | Crowley 2002 | Benzodiazepines: good practice guidelines for clinicians; 2002:1–28. Enlace. Accessed September 2023. |  |  | 1 |  |  |  |  |  |  |  |  |  |  |  |
| 165 | Baillargeon 2003 | Discontinuation of benzodiazepines among older insomniac adults treated with cognitive-behavioural therapy combined with gradual tapering: a randomized trial. CMAJ 2003; 169: 1015–20. |  |  |  | 1 |  |  |  |  |  | 1 |  |  | 1 |  |
| 166 | Cardinali 2002 | A double blind–placebo controlled study on melatonin efficacy to reduce anxiolytic benzodiazepine use in the elderly. Neuro Endocrinol Lett 2002; 23: 55–60. |  |  |  | 1 |  |  |  |  |  |  |  |  |  |  |
| 167 | Giblin 1983 | Sleep without drugs. J R Coll Gen Pract 1983; 33: 628–33. |  |  |  | 1 |  |  |  |  |  |  |  |  |  |  |
| 168 | Habraken 1997 | Gradual withdrawal from benzodiazepines in residents of homes for the elderly: experience and suggestions for future research. Eur J Clin Pharmacol 1997; 51: 355–8. |  |  |  | 1 |  |  |  |  |  |  |  |  |  |  |
| 169 | Morin 2004 | Randomized clinical trial of supervised tapering and cognitive behavior therapy to facilitate benzodiazepine discontinuation in older adults with chronic insomnia. Am J Psychiatry 2004; 161: 332–42. |  |  |  | 1 |  |  |  |  |  | 1 |  |  | 1 |  |
| 170 | Petrovic 2002 | Fast withdrawal from benzodiazepines in geriatric inpatients: a randomised double-blind, placebo-controlled trial. Eur J Clin Pharmacol 2002; 57: 759–64. |  |  |  | 1 |  |  |  |  |  |  |  |  |  |  |
| 171 | Salonoja 2010 | One-time counselling decreases the use of benzodiazepines and related drugs among community-dwelling older persons. Age Ageing 2010; 39: 313–9. |  |  |  | 1 |  |  |  |  |  |  |  |  |  |  |
| 172 | Tham 1989 | Temazepam withdrawal in elderly hospitalized patients – a double-blind randomized trial comparing abrupt versus gradual withdrawal. Ir J Med Sci 1989; 158: 294–9. |  |  |  | 1 |  |  |  |  |  |  |  |  |  |  |
| 173 | Velert Vila 2011 | Intervención Farmacéutica para la Adecuación de la Prescripción de Benzodiazepinas en Pacientes Mayores. Cardinal Herrera University, 2011. |  |  |  | 1 |  |  |  |  |  |  |  |  |  |  |
| 174 | Velert Vila 2012 | Adecuación de la utilización de benzodiazepinas en ancianos desde la oficina de farmacia. Un estudio de colaboración médicofarmacéutico. Aten Primaria 2012; 44: 402–10. |  |  |  | 1 |  |  |  |  |  |  |  |  |  |  |
| 175 | Avorn 1992 | A randomized trial of a program to reduce the use of psychoactive drugs in nursing homes. N Engl J Med 1992; 327: 168–73. |  |  |  | 1 |  |  |  |  |  |  |  |  |  |  |
| 176 | Crotty 2004 | An outreach intervention to implement evidence based practice in residential care: a randomized controlled trial. BMC Health Serv Res 2004; 4:6. |  |  |  | 1 |  |  |  |  |  |  |  | 1 |  |  |
| 177 | Pit 2007 | A Quality Use of Medicines program for general practitioners and older people: a cluster randomised controlled trial. Med J Aust 2007; 187: 23–30. |  |  |  | 1 |  |  |  |  |  |  |  |  |  |  |
| 178 | Rikala 2011 | The effects of medication assessment on psychotropic drug use in the community-dwelling elderly. Int Psychogeriatr 2011; 23: 473–84. |  |  |  | 1 |  |  |  |  |  |  |  |  |  |  |
| 179 | Roberts 2001 | Outcomes of a randomized controlled trial of a clinical pharmacy intervention in 52 nursing homes. Br J Clin Pharmacol 2001; 51: 257–65. |  |  |  | 1 |  |  |  |  |  |  |  | 1 |  |  |
| 180 | Strikwerda 1994 | Drug therapy in a nursing home; favorable effect of feedback by the pharmacist on family physician’s prescribing behavior. Ned Tijdschr Geneeskd 1994; 138: 1770–4. |  |  |  | 1 |  |  |  |  |  |  |  |  |  |  |
| 181 | Heather N., 2004 | Heather N., Bowie A., Ashton H., McAvoy B., Spencer I., Brodie J. et al. Randomised controlled trial of two brief interventions against long-term benzodiazepine use: outcome of intervention. Addict Res Theory 2004; 12:141–54. |  |  |  |  | 1 |  | 1 |  |  |  |  | 1 |  |  |
| 182 | Bashir K., 1994 | Bashir K., King M., Ashworth M. Controlled evaluation of brief intervention by general practitioners to reduce chronic use of benzodiazepines. Br J Gen Pract 1994; 44:408–12. |  |  |  |  | 1 |  | 1 |  |  |  |  | 1 |  |  |
| 183 | Cormack 1994 | Cormack M. A., Sweeney K. G., Hughes-Jones H., Foot G. A. Evaluation of an easy, cost-effective strategy for cutting benzodiazepine use in general practice. Br J Gen Pract 1994; 44: 5–8. |  |  |  |  | 1 |  | 1 |  |  |  |  | 1 | 1 |  |
| 184 | Vicens C., 2006 | Vicens C., Fiol F., Llobera J., Campoamor F., Mateu C., Alegret S. et al. Withdrawal from long-term benzodiazepine use: randomised trial in family practice. Br J Gen Pract 2006; 56: 958–63. |  |  |  |  | 1 |  |  |  |  |  |  |  |  |  |
| 185 | Kuntz J. L., 2019 | Kuntz J. L., Kouch L., Christian D., Hu W., Peterson P. L. Patient education and pharmacist consultation influence on nonbenzodiazepine sedative medication deprescribing success for older adults. Perm J 2019; 23:18–61. |  |  |  |  | 1 |  |  |  |  |  |  |  |  |  |
| 186 | Navy H. J., 2018 | Navy H. J., Weffald L., Delate T., Patel R. J., Dugan J. P. Clinical pharmacist intervention to engage older adults in reducing use of alprazolam. Consult Pharm 2018; 33:711–22. |  |  |  |  | 1 |  |  |  |  |  |  |  |  |  |
| 187 | Vicens C., 2014 | Vicens C., Bejarano F., Sempere E., Mateu C., Fiol F., Socias I. et al. Comparative efficacy of two interventions to discontinue long-term benzodiazepine use: cluster randomised controlled trial in primary care. Br J Psychiatry 2014; 204:471–9. |  |  |  |  | 1 |  |  |  |  |  |  |  | 1 |  |
| 188 | Ashworth 2021 | Ashworth N, Kain N, Wiebe D, et al. Reducing prescribing of benzodiazepines in older adults: a comparison of four physician-focused interventions by a medical regulatory authority. BMC Fam Pract. 2021;22(1):68. https://doi.org/10.1186/ s12875-021-01415-x. 24. |  |  |  |  |  | 1 |  |  |  |  |  |  |  |  |
| 189 | Evrard 2020 | Evrard P, Henrard S, Foulon V, et al. Benzodiazepine use and Deprescribing in Belgian nursing homes: results from the COMEON study. J Am Geriatr Soc. 2020;68(12):2768–77. https://doi. org/10.1111/jgs.16751. |  |  |  |  |  | 1 |  |  |  |  |  |  |  |  |
| 190 | Navy 2018 | Navy HJ, Weffald L, Delate T, et al. Clinical pharmacist intervention to engage older adults in reducing use of alprazolam. Consult Pharm. 2018;33(12):711–22. https://doi.org/10.4140/TCP.n.2018. 711. |  |  |  |  |  | 1 |  |  |  |  |  |  |  |  |
| 191 | Roberts 2001 | Roberts MS, Stokes JA, King MA, et al. Outcomes of a randomized controlled trial of a clinical pharmacy intervention in 52 nursing homes. Br J Clin Pharmacol. 2001;51(3):257–65. https:// doi.org/10.1046/j.1365-2125.2001.00347.x. |  |  |  |  |  | 1 |  |  |  |  |  |  |  |  |
| 192 | Martin 2017 | P. Martin, C. Tannenbaum. Use of the EMPOWER brochure to deprescribe sedative-hypnotic drugs in older adults with mild cognitive impairment. BMC Geriatr. 2017;17:37. |  |  |  |  |  |  |  | 1 |  |  |  |  |  |  |
| 193 | Gnjidic 2019 | D. Gnjidic, H. Ong, C. Leung, et al. The impact of in-hospital patient-education intervention on older people’s attitudes and intention to have their benzodiazepines deprescribed: a feasibility study. Ther Adv Drug Saf. 2019;10:2042098618816562. |  |  |  |  |  |  |  | 1 |  |  |  |  |  |  |
| 194 | Hagen 2005 | B.F. Hagen, C. Armstrong-Esther, et al. Neuroleptic and benzodiazepine use in nursing homes: characteristics and changes following pharmacist intervention. Int Psychogeriatr. 2005;17(4):631–652. |  |  |  |  |  |  |  | 1 |  |  |  |  |  |  |
| 195 | Morgan 2002 | J.D. Morgan, D.J. Wright, H. Chrystyn. Pharmacoeconomic evaluation of a patient education letter aimed at reducing long-term prescribing of benzodiazepines. Pharm World Sci. 2002;24(6):231–235. |  |  |  |  |  |  |  | 1 |  |  |  |  |  |  |
| 196 | Luchen 2019 | G.G. Luchen, E.S. Prohaska, J.F. Ruisinger, et al. Impact of community pharmacist intervention on concurrent benzodiazepine and opioid prescribing patterns. J Am Pharm Assoc. 2019;59(2):238–242. |  |  |  |  |  |  |  | 1 |  |  |  |  |  |  |
| 197 | Shayegani 2018 | R. Shayegani, P. Panneman, K. Van Dyke, et al. Reducing co-prescriptions of benzodiazepines and opioids in a veteran population. Am J Manag Care. 2018;24(8):e265–e269. |  |  |  |  |  |  |  | 1 |  |  |  |  |  |  |
| 198 | Harden 2015 | P. Harden, S. Ahmed, K. Ang, et al. Clinical implications of tapering chronic opioids in a veteran population. Pain Med. 2015;16(10):1975–1981. |  |  |  |  |  |  |  | 1 |  |  |  |  |  |  |
| 199 | Geka 2019 | M. Geka, K. Inoue, et al. Effectiveness of pharmacist-convened multidisciplinary clinical team meetings in promoting appropriate benzodiazepine receptor agonist use. Yakugaku Zasshi. 2019;139(6):931–937. |  |  |  |  |  |  |  | 1 |  |  |  |  |  |  |
| 200 | Furbish 2017 | S.M.L. Furbish, M.E. Kroehl, D.F. Loeb, et al. A pharmacist-physician intervention for reducing benzodiazepine prescribing among older adults. J Am Geriatr Soc. 2017;65(1):70–76. |  |  |  |  |  |  |  | 1 |  |  |  |  |  |  |
| 201 | Westbury 2010 | J. Westbury, S. Jackson, P. Gee, et al. An effective approach to decrease antipsychotic and benzodiazepine use in nursing homes: the RedUSe project. Int Psychogeriatr. 2010;22(1):26–36. |  |  |  |  |  |  |  | 1 |  |  |  |  |  |  |
| 202 | Darchuk 2010 | K.M. Darchuk, C.O. Townsend, J.D. Rome, et al. Longitudinal treatment outcomes for geriatric patients with chronic non-cancer pain at an interdisciplinary pain rehabilitation program. Pain Med. 2010;11(9):1352–1364. |  |  |  |  |  |  |  | 1 |  |  |  |  |  |  |
| 203 | Hopkins 1982 | Hopkins DR, Sethi KB, Mucklow JC. Benzodiazepine withdrawal in general practice. J R Coll Gen Pract 1982;32(245):758-62. |  |  |  |  |  |  |  |  | 1 | 1 |  |  |  |  |
| 204 | Schweizer 1998 | Schweizer E, Rickels K, Case WG, Greenblatt DJ. Long-term therapeutic use of benzodiazepines. Psychol Med 1998;28(3):713-20 |  |  |  |  |  |  |  |  | 1 |  |  |  |  |  |
| 205 | Tyrer 1983 | Tyrer P, Owen R, Dawling S. Gradual withdrawal of diazepam after long-term therapy. Lancet 1983;1(8339):1402-6 |  |  |  |  |  |  |  |  | 1 |  |  |  |  |  |
| 206 | Schweizer 1990 | Schweizer E, Rickels K, Case WG, Greenblatt DJ. Long-term therapeutic use of benzodiazepines. II. Effects of gradual taper. Arch Gen Psychiatry 1990;47(10):908-15. |  |  |  |  |  |  |  |  | 1 | 1 |  |  |  |  |
| 207 | Couvee 2002 | Couvee JE, Timmermans MA, Zitman FG; Dutch Chronic Benzodiazepine Working Group. The long-term outcome of a benzodiazepine discontinuation programme in depressed outpatients. J Affect Disord 2002;70:133-41 |  |  |  |  |  |  |  |  | 1 |  |  |  |  |  |
| 208 | Morin 2004 | Morin CM, Bastien C, Guay B, et al. Randomized clinical trial of supervised tapering and cognitive behavior therapy to facilitate benzodiazepine discontinuation in older adults with chronic insomnia. Am J Psychiatry 2004;161(2):332-42 |  |  |  |  |  |  |  |  | 1 |  |  |  |  |  |
| 209 | Morin 2005 | Morin CM, Bélanger L, Bastien C, Vallières A. Long-term outcome after discontinuation of benzodiazepines for insomnia: a survival analysis of relapse. Behav Res Ther 2005;43(1):1-14 |  |  |  |  |  |  |  |  | 1 | 1 |  |  | 1 |  |
| 210 | Belanger 2005 | Belanger L, Morin CM, Bastien C. Benzodiazepine discontinuation among adults with GAD: a randomized trial of cognitive-behavioural therapy. Behav Res Ther 2005;43(1):1-14 |  |  |  |  |  |  |  |  | 1 | 1 |  |  | 1 |  |
| 211 | Baillargeon 2003 | Baillargeon L, Landreville P, Verreault R, Beauchemin JP, Gregoire JP, Morin CM. Discontinuation of benzodiazepines among older insomniac adults treated with cognitive-behavioural therapy combined with gradual tapering: a randomized trial. CMAJ 2003;169(10):1015-20 |  |  |  |  |  |  |  |  | 1 |  |  |  |  |  |
| 212 | Zitman 2002 | Zitman FG, Couvee JE. Chronic benzodiazepine use in general practice: psychometric evaluation. Addiction 2002;97(3):337-42 |  |  |  |  |  |  |  |  | 1 |  |  |  |  |  |
| 213 | Rickels 1999 | Rickels K, Schweizer E, Case WG, Greenblatt DJ. Tapering benzodiazepine use with paroxetine. Psychopharmacology (Berl) 1999;141(1):1-5 |  |  |  |  |  |  |  |  | 1 |  |  |  |  |  |
| 214 | Udelman 1990 | Udelman HD, Udelman DL. Concurrent use of buspirone in anxious patients during withdrawal from alprazolam therapy. J Clin Psychiatry 1990;51(9):46-52 |  |  |  |  |  |  |  |  | 1 |  |  |  |  |  |
| 215 | Voshaar 2006 | Voshaar RC, Gorgels WJ, Mol AJ, et al. Tapering off long-term benzodiazepine use with or without group cognitive therapy. Pharm World Sci 2006;29(6):641-6 |  |  |  |  |  |  |  |  | 1 |  |  |  |  |  |
| 216 | Voshaar 2007 | Mol AJ, Oude Voshaar RC, Gorgels WJ, et al. Benzodiazepine craving in a general practice trial. J Clin Psychiatry 2007;68(12):1894-900 |  |  |  |  |  |  |  |  | 1 |  |  |  |  |  |
| 217 | Nardi 2010 | Nardi AE, Freire RC, Valença AM, et al. Tapering clonazepam in patients with panic disorder after at least 3 years of treatment. J Clin Psychopharmacol 2010;30(3):290-3. |  |  |  |  |  |  |  |  |  | 1 |  |  |  |  |
| 218 | Tyrer 1983 | Tyrer P, Owen R, Dawling S. Gradual withdrawal of diazepam after long-term therapy. Lancet 1983;1(8339):1402-6. |  |  |  |  |  |  |  |  |  | 1 |  |  |  |  |
| 219 | Busto 1986 | Busto U, Sellers EM, Naranjo CA, et al. Withdrawal reaction after long-term therapeutic use of benzodiazepines. N Engl J Med 1986;315(14):854-9. |  |  |  |  |  |  |  |  |  | 1 |  |  |  |  |
| 220 | Couvee 2002 | Couvee JE, Zitman FG. The benzodiazepine withdrawal symptom questionnaire: psychometric evaluation during a discontinuation program in depressed chronic benzodiazepine users in general practice. Addiction 2002;97(3):337-45. |  |  |  |  |  |  |  |  |  | 1 |  |  |  |  |
| 221 | Schweizer 1989 | Schweizer E, Case WG, Rickels K. Benzodiazepine dependence and withdrawal in elderly patients. Am J Psychiatry 1989;146(4):529-31. |  |  |  |  |  |  |  |  |  | 1 |  |  |  |  |
| 222 | Gosselin 2006 | Gosselin P, Ladouceur R, Morin CM, et al. Benzodiazepine discontinuation among adults with GAD: a randomized trial of cognitive-behavioral therapy. J Consult Clin Psychol 2006;74(5):908-19. |  |  |  |  |  |  |  |  |  | 1 |  |  | 1 |  |
| 223 | Vicens 2006 | Vicens C, Fiol F, Llobera J, et al. Withdrawal from long-term benzodiazepine use: randomised trial in family practice. Br J Gen Pract 2006;56(533):958-63. |  |  |  |  |  |  |  |  |  | 1 |  |  |  |  |
| 224 | Voshaar 2003 | Voshaar RC, Gorgels WJ, Mol AJ, et al. Tapering off long-term benzodiazepine use with or without group cognitive-behavioural therapy: three-conditioned, randomised controlled trial. Br J Psychiatry 2003;182:498-504. |  |  |  |  |  |  |  |  |  | 1 |  |  | 1 |  |
| 225 | Schweizer 1998 | Schweizer E, Rickels K, De Martinis N, et al. The effect of personality on withdrawal severity and taper outcome in benzodiazepine dependent patients. Psychol Med 1998;28(3):713-20. |  |  |  |  |  |  |  |  |  | 1 |  |  |  |  |
| 226 | Voshaar 2006 | Voshaar RC, Gorgels WJ, Mol AJ, et al. Predictors of long-term benzodiazepine abstinence in participants of a randomized controlled benzodiazepine withdrawal program. Can J Psychiatry 2006;51(7):445-52. |  |  |  |  |  |  |  |  |  | 1 |  |  |  |  |
| 227 | Mol 2006 | Mol AJ, Oude Voshaar RC, Gorgels WJ, et al. The absence of benzodiazepine craving in a general practice benzodiazepine discontinuation trial. Addict Behav 2006;31(2):211-22. |  |  |  |  |  |  |  |  |  | 1 |  |  |  |  |
| 228 | Mol 2007 | Mol AJ, Oude Voshaar RC, Gorgels WJ, et al. The role of craving in relapse after discontinuation of long-term benzodiazepine use. J Clin Psychiatry 2007;68(12):1894-900. |  |  |  |  |  |  |  |  |  | 1 |  |  |  |  |
| 229 | Couvee 2002 | Couvee JE, Timmermans MA, Zitman FG; Dutch Chronic Benzodiazepine Working Group. The long-term outcome of a benzodiazepine discontinuation programme in depressed outpatients. J Affect Disord 2002;70:133-41. |  |  |  |  |  |  |  |  |  | 1 |  |  |  |  |
| 230 | Couvee 2002 | Couvee JE, Bakker A, Zitman FG. The relevance of psychiatric and somatic comorbidity in depressed chronic benzodiazepine users. Psychother Psychosom 2002;71(5):263-8. |  |  |  |  |  |  |  |  |  | 1 |  |  |  |  |
| 231 | Curran 2003 | Curran HV, Collins R, Fletcher S, et al. Older adults and withdrawal from benzodiazepine hypnotics in general practice: effects on cognitive function, sleep, mood and quality of life. Psychol Med 2003;33(7):1223-37. |  |  |  |  |  |  |  |  |  | 1 |  |  |  |  |
| 232 | Schweizer 1995 | Schweizer E, Case WG, Garcia-Espana F, et al. Progesterone co-administration in patients discontinuing long-term benzodiazepine therapy: effects on withdrawal severity and taper outcome. Psychopharmacology (Berl) 1995;117(4):424-9. |  |  |  |  |  |  |  |  |  | 1 |  |  |  |  |
| 233 | Vissers 2007 | Vissers FH, Knipschild PG, Crebolder HF. Is melatonin helpful in stopping the long-term use of hypnotics? A discontinuation trial. Pharm World Sci 2007;29(6):641-6. |  |  |  |  |  |  |  |  |  | 1 |  |  |  |  |
| 234 | Chen et al., 2010 | Chen DR, Sethi KB, Mucklow JC. Discontinuing Benzodiazepine Therapy: An Interdisciplinary Approach at a Geriatric Day Hospital. Can. Pharm. J. 2010, 143, 286–295.e3. |  |  |  |  |  |  |  |  |  |  | 1 |  |  |  |
| 235 | Canham et al., 2014 | Canham, S.L.; Gallo, J.; Simoni-Wastila, L. Perceptions of Benzodiazepine Dependence Among Women Age 65 and Older. J. Gerontol. Soc. Work. 2014, 57, 872–888. |  |  |  |  |  |  |  |  |  |  | 1 |  |  |  |
| 236 | Heser et al., 2018 | Heser, K.; Pohontsch, N.J.; Scherer, M.; Löffler, A.; Luck, T.; Riedel-Heller, S.G.; Maier, W.; Parker, D.; Haenisch, B.; Jessen, F. Perspective of elderly patients on chronic use of potentially inappropriate medication–Results of the qualitative CIM-TRIAD study. PLoS ONE 2018, 13, e0202068. |  |  |  |  |  |  |  |  |  |  | 1 |  |  |  |
| 237 | Martin y Tannenbaum, 2017 | Martin, P.; Tannenbaum, C. A realist evaluation of patients’ decisions to deprescribe in the EMPOWER trial. BMJ Open 2017, 7, e015959. |  |  |  |  |  |  |  |  |  |  | 1 |  |  |  |
| 238 | Williams et al., 2016 | Williams, F.; Mahfouz, C.; Bonney, A.; Pearson, R.; Seidel, B.; Dijkmans-Hadley, B.; Ivers, R. A circle of silence: The attitudes of patients older than 65 years of age to ceasing long-term sleeping tablets. Aust. Fam. Physician 2016, 45, 506–511. |  |  |  |  |  |  |  |  |  |  | 1 |  |  |  |
| 239 | Cook et al., 2007 | Cook, J.M.; Marshall, R.; Masci, C.; Coyne, J.C. Physicians’ Perspectives on Prescribing Benzodiazepines for Older Adults: A Qualitative Study. J. Gen. Intern. Med. 2007, 22, 303–307. |  |  |  |  |  |  |  |  |  |  | 1 |  |  |  |
| 240 | Šubelj et al., 2010 | Šubelj, M.; Vidmar, G.; Švab, V. Prescription of benzodiazepines in Slovenian family medicine: A qualitative study. Wien. Klin. Wochenschr. 2010, 122, 474–478. |  |  |  |  |  |  |  |  |  |  | 1 |  |  |  |
| 241 | Anthierens et al., 2009 | Anthierens, S.; Grypdonck, M.; De Pauw, L.; Christiaens, T. Perceptions of nurses in nursing homes on the usage of benzodiazepines. J. Clin. Nurs. 2009, 18, 3098–3106. |  |  |  |  |  |  |  |  |  |  | 1 |  |  |  |
| 242 | Pickering et al., 2020 | Pickering, A.N.; Hamm, M.E.; Bs, A.D.; Hanlon, J.T.; Thorpe, C.T.; Gellad, W.F.; Radomski, T.R. Older Patient and Caregiver Perspectives on Medication Value and Deprescribing: A Qualitative Study. J. Am. Geriatr. Soc. 2020, 68, 746–753. |  |  |  |  |  |  |  |  |  |  | 1 |  |  |  |
| 243 | Cormack 1989 | Cormack MA, Owens RG, Dewey ME: The effect of minimal interventions by general practitioners on long-term benzodiazepine use. J R Coll Gen Pract. 1989, 39 (327): 408-411. |  |  |  |  |  |  |  |  |  |  |  | 1 |  |  |
| 244 | Gorgels 2005 | Gorgels WJMJ, Oude Voshaar RC, Mol AJJ, Van De Lisdonk EH, Van Balkom AJLM, Van Den Hoogen HJM, Mulder J, Breteler MHM, Zitman FG: Discontinuation of long-term benzodiazepine use by sending a letter to users in family practice: A prospective controlled intervention study. Drug and Alcohol Dependence. 2005, 78 (1): 49-56. 10.1016/j.drugalcdep.2004.09.001. |  |  |  |  |  |  |  |  |  |  |  | 1 |  |  |
| 245 | Morrison 1990 | Morrison JM: Audit and follow-up of chronic benzodiazepine tranquillizer use in one general practice. Family Practice. 1990, 7 (4): 253-257. 10.1093/fampra/7.4.253. |  |  |  |  |  |  |  |  |  |  |  | 1 |  |  |
| 246 | Zwar 2000 | Zwar NA, Wolk J, Gordon JJ, Sanson-Fisher RW: Benzodiazepine prescribing by GP registrars. A trial of educational outreach. Aust Fam Physician. 2000, 29 (11): 1104-1107. |  |  |  |  |  |  |  |  |  |  |  | 1 |  |  |
| 247 | Cormack 1999 | Cormack MA, Sweeney KG, Hughes-Jones H, Foot GA. Evaluation of an easy, cost-effective strategy for cutting benzodiazepine use in general practice. Br J Gen Pract. 1994;44(378):5-8. |  |  |  |  |  |  |  |  |  |  |  | 1 |  |  |
| 248 | Midlov 2006 | Midlov P, Bondesson A, Eriksson T, Nerbrand C, Hoglund P: Effects of educational outreach visits on prescribing of benzodiazepines and antipsychotic drugs to elderly patients in primary health care in southern Sweden. Family Practice. 2006, 23 (1): 60-64. 10.1093/fampra/cmi105. |  |  |  |  |  |  |  |  |  |  |  | 1 |  |  |
| 249 | Smith 1998 | Smith DH, Christensen DB, Stergachis A, Holmes G: A randomized controlled trial of a drug use review intervention for sedative hypnotic medications. Med Care. 1998, 36 (7): 1013-1021. 10.1097/00005650-199807000-00008. |  |  |  |  |  |  |  |  |  |  |  | 1 |  |  |
| 250 | Dollman 2005 | Dollman WB, LeBlanc VT, Stevens L, O'Connor PJ, Roughead EE, Gilbert AL. Achieving a sustained reduction in benzodiazepine use through implementation of an area-wide multi-strategic approach. J Clin Pharm Ther. 2005;30(5):425-432. https://doi.org/10.1111/j.1365-2710.2005.00674.x. |  |  |  |  |  |  |  |  |  |  |  | 1 |  |  |
| 251 | Towle 2006 | Towle I, Adams J. A novel, pharmacist-led strategy to reduce the prescribing of benzodiazepines in Paisley. Pharm J. 2006;276:136-138. |  |  |  |  |  |  |  |  |  |  |  | 1 |  |  |
| 252 | Brymer 2000 | Brymer C, Rusnell I. Reducing substance dependence in elderly people: The side effects program. J Popul Ther Clin Pharmacol. 2000;7(3):161-166. |  |  |  |  |  |  |  |  |  |  |  | 1 |  |  |
| 253 | Onyett 1988 | Onyett SR, Turpin G. Benzodiazepine withdrawal in primary care: A comparison of behavioural group training and individual sessions. Behav Psychother. 1988;16(4):297-312. https://doi.org/10.1017/S0141347300014154. |  |  |  |  |  |  |  |  |  |  |  | 1 |  |  |
| 254 | de Burgh 1995 | de Burgh S, Mant A, Mattick RP, Donnelly N, Hall W, Bridges-Webb C. A controlled trial of educational visiting to improve benzodiazepine prescribing in general practice. Aust J Public Health. 1995;19(2):142-148. https://doi.org/10.1111/j.1753-6405.1995.tb00364.x. |  |  |  |  |  |  |  |  |  |  |  | 1 |  |  |
| 255 | Smith 1998 | Smith DH, Christensen DB, Stergachis A, Holmes G. A randomized controlled trial of a drug use review intervention for sedative hypnotic medications. Med Care. 1998;36(7):1013-1021. https://doi.org/10.1097/00005650-199807000-00008. |  |  |  |  |  |  |  |  |  |  |  | 1 |  |  |
| 256 | Berings 1994 | Berings D, Blondeel L, Habraken H. The effect of industry-independent drug information on the prescribing of benzodiazepines in general practice. Eur J Clin Pharmacol. 1994;46(6):501-505. https://doi.org/10.1007/BF00196105. |  |  |  |  |  |  |  |  |  |  |  | 1 |  |  |
| 257 | Holm 1990 | Holm M. Intervention against long-term use of hypnotics/sedatives in general practice. Scand J Prim Health Care. 1990;8(2):113-117. https://doi.org/10.3109/02813439008994941. |  |  |  |  |  |  |  |  |  |  |  | 1 |  |  |
| 258 | Eide 2001 | Eide E, Schjott J. Assessing the effects of an intervention by a pharmacist on prescribing and administration of hypnotics in nursing homes. Pharm World Sci. 2001;23(6):227-231. https://doi.org/10.1023/A:1015142211348. |  |  |  |  |  |  |  |  |  |  |  | 1 |  |  |
| 259 | Batty 2001 | Batty GM, Oborne CA, Hooper R, Jackson SHD. Investigating intervention strategies to increase the appropriate use of benzodiazepines in elderly medical in-patients. Br J Clin Govern. 2001;6(4):252-258. https://doi.org/10.1108/14664100110408608. |  |  |  |  |  |  |  |  |  |  |  | 1 |  |  |
| 260 | Gilbert 1993 | Gilbert A, Owen N, Innes JM, Sansom L. Trial of an intervention to reduce chronic benzodiazepine use among residents of aged-care accommodation. Aust N Z J Med. 1993;23(4):343-347. |  |  |  |  |  |  |  |  |  |  |  | 1 |  |  |
| 261 | Elliott 2001 | Elliott RA, Woodward MC, Oborne CA. Improving benzodiazepine prescribing for elderly hospital inpatients using audit and multidisciplinary feedback. Intern Med J. 2001;31(9):529-535. https://doi.org/10.1046/j.1445-5994.2001.00139.x. |  |  |  |  |  |  |  |  |  |  |  | 1 |  |  |
| 262 | Holden 1994 | Holden JD, Hughes IM, Tree A. Benzodiazepine prescribing and withdrawal for 3234 patients in 15 general practices. Fam Pract. 1994;11(4):358-362. https://doi.org/10.1093/fampra/11.4.358. |  |  |  |  |  |  |  |  |  |  |  | 1 |  |  |
| 263 | Pimlott 2003 | Pimlott NJG, Hux JE, Wilson LM, Kahan M, Li C, Rosser WW. Educating physicians to reduce benzodiazepine use by elderly patients: A randomized controlled trial. CMAJ. 2003;168(7):835-839. |  |  |  |  |  |  |  |  |  |  |  | 1 |  |  |
| 264 | Schmidt 1998 | Schmidt I, Claesson CB, Westerholm B, Nilsson LG, Svarstad BL: The impact of regular multidisciplinary team interventions on psychotropic prescribing in Swedish nursing homes. Journal of the American Geriatrics Society. 1998, 46 (1): 77-82. |  |  |  |  |  |  |  |  |  |  |  | 1 |  |  |
| 265 | Cantopher 1990 | Cantopher T, Olivieri S, Cleave N, Edwards JG. Chronic benzodiazepine dependence. A comparative study of abrupt withdrawal under propranolol cover versus gradual withdrawal. Br J Psychiatry. 1990;156:406–411. |  |  |  |  |  |  |  |  |  |  |  |  |  | 1 |
| 266 | Petrovic 1999 | Petrovic M, Pevernagie D, Van Den Noortgate N, Mariman A, Michielsen W, Afschrift M. A programme for short-term withdrawal from benzodiazepines in geriatric hospital inpatients: Success rate and effect on subjective sleep quality. Int J Geriatr Psychiatry. 1999;14:754–760. |  |  |  |  |  |  |  |  |  |  |  |  |  | 1 |
| 267 | Delle Chiaie 1995 | Delle Chiaie R, Pancheri P, Casacchia M, Stratta P, Kotzalidis GD, Zibellini M. Assessment of the efficacy of buspirone in patients affected by generalized anxiety disorder, shifting to buspirone from prior treatment with lorazepam: a placebo-controlled, double-blind study. J Clin Psychopharmacol. 1995;15:12–19. |  |  |  |  |  |  |  |  |  |  |  |  |  | 1 |
| 268 | Rickels 2000 | Rickels K, DeMartinis N, García-España F, Greenblatt DJ, Mandos LA, Rynn M. Imipramine and buspirone in treatment of patients with generalized anxiety disorder who are discontinuing long-term benzodiazepine therapy. Am J Psychiatry. 2000;157:1973–1979. |  |  |  |  |  |  |  |  |  |  |  |  |  | 1 |
| 269 | Rynn 2003 | Rynn M, García-España F, Greenblatt DJ, Mandos LA, Schweizer E, Rickels K. Imipramine and buspirone in patients with panic disorder who are discontinuing long-term benzodiazepine therapy. J Clin Psychopharmacol. 2003;23:505–508. |  |  |  |  |  |  |  |  |  |  |  |  |  | 1 |
| 270 | Croissant 2008 | Croissant B, Grosshans M, Diehl A, Mann K. Oxcarbazepine in rapid benzodiazepine detoxification. Am J Drug Alcohol Abuse. 2008;34:534–540. |  |  |  |  |  |  |  |  |  |  |  |  |  | 1 |
| 271 | Ries 1989 | Ries RK, Roy-Byrne PP, Ward NG, Neppe V, Cullison S. Carbamazepine treatment for benzodiazepine withdrawal. Am J Psychiatry. 1989;146:536–537. |  |  |  |  |  |  |  |  |  |  |  |  |  | 1 |
| 272 | Garcia-Borreguero 1991 | Garcia-Borreguero D, Bronisch T, Apelt S, Yassouridis A, Emrich HM. Treatment of benzodiazepine withdrawal symptoms with carbamazepine. Eur Arch Psychiatry Clin Neurosci. 1991;241:145–150. |  |  |  |  |  |  |  |  |  |  |  |  |  | 1 |
| 273 | Bobes 2012 | Bobes J, Rubio G, Terán A, et al. Pregabalin for the discontinuation of long-term benzodiazepines use: an assessment of its effectiveness in daily clinical practice. Eur Psychiatry. 2012;27:301–307. |  |  |  |  |  |  |  |  |  |  |  |  |  | 1 |
| 274 | Tyrer 1981 | Tyrer P, Rutherford D, Huggett T. Benzodiazepine withdrawal symptoms and propranolol. Lancet. 1981;1:520–522. |  |  |  |  |  |  |  |  |  |  |  |  |  | 1 |
